# Antimicrobial use guidelines for canine pyoderma by the International Society for Companion Animal Infectious Diseases (ISCAID)

**DOI:** 10.1111/vde.13342

**Published:** 2025-05-07

**Authors:** Anette Loeffler, Christine L. Cain, Lluís Ferrer, Koji Nishifuji, Katarina Varjonen, Mark G. Papich, Luca Guardabassi, Siân M. Frosini, Emi N. Barker, J. Scott Weese

**Affiliations:** ^1^ Department of Clinical Science and Services Royal Veterinary College Hatfield UK; ^2^ Department of Clinical Sciences and Advanced Medicine University of Pennsylvania, School of Veterinary Medicine Philadelphia Pennsylvania USA; ^3^ Department of Animal Medicine and Surgery Universitat Autònoma de Barcelona Barcelona Spain; ^4^ Division of Animal Life Science Graduate School of Agriculture, Tokyo University of Agriculture and Technology Tokyo Japan; ^5^ Anicura Albano Animal Hospital Danderyd Sweden; ^6^ College of Veterinary Medicine North Carolina State University Raleigh North Carolina USA; ^7^ Department of Veterinary and Animal Sciences University of Copenhagen Frederiksberg Denmark; ^8^ Langford Vets and Bristol Veterinary School University of Bristol Bristol UK; ^9^ Ontario Veterinary College University of Guelph Guelph Ontario Canada

## Abstract

**Background:**

Canine pyoderma is one of the most common presentations in small animal practice, frequently leading to antimicrobial prescribing.

**Objectives:**

To provide clinicians with antimicrobial treatment guidelines for staphylococcal pyoderma, including those involving meticillin‐resistant staphylococci. Guidance on diagnosing surface, superficial and deep pyoderma and their underlying primary causes, is included. Recommendations aim to optimise treatment outcomes while promoting responsible antimicrobial use.

**Materials and Methods:**

Evidence was gathered from a systematic literature review of English‐language treatment studies for canine pyoderma up to 23 December 2023. Quality was assessed using SORT criteria and combined with authors' consensus evaluation. Recommendations were voted on in an iterative process, followed by a Delphi‐style feedback process before final agreement by the authors.

**Results:**

Cytology should be performed in all cases before antimicrobials are used. Topical antimicrobial therapy alone is the treatment‐of‐choice for surface and superficial pyodermas. Systemic antimicrobials should be reserved for deep pyoderma and for superficial pyoderma when topical therapy is not effective. Systemic therapy, with adjunctive topical treatment, is initially provided for 2 weeks in superficial and 3 weeks in deep pyoderma, followed by re‐examination to assess progress and manage primary causes. First‐choice drugs have expected efficacy against the majority of meticillin‐susceptible *Staphylococcus pseudintermedius;* for all other drugs, laboratory testing should confirm susceptibility and exclude suitability of safer alternatives. As culture and susceptibility testing are essential for rationalising systemic therapy, laboratories and practices should price them reasonably to encourage use. Proactive topical therapy using antiseptics may help prevent recurrences.

**Conclusions and Clinical Relevance:**

The accessibility of the skin offers excellent, achievable opportunities for antimicrobial stewardship.

## Graphical abstract

**Background** – Canine pyoderma is one of the most common presentations in small animal practice, frequently leading to antimicrobial prescribing. **Objectives** – To provide clinicians with antimicrobial treatment guidelines for staphylococcal pyoderma, including those involving meticillin‐resistant staphylococci. Guidance on diagnosing surface, superficial and deep pyoderma and their underlying primary causes, is included. Recommendations aim to optimise treatment outcomes while promoting responsible antimicrobial use. **Conclusions and Clinical Relevance** – The accessibility of the skin offers excellent, achievable opportunities for antimicrobial stewardship.

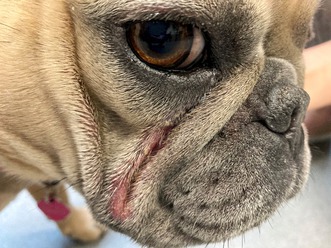

## Zusammenfassung

**Hintergrund:**

Die canine Pyodermie ist eine der häufigsten Vorstellungen in der Kleintierpraxis, was häufig zu einer Verschreibung von Antibiotika führt.

**Ziele:**

Das Ziel war es, KlinikerInnen mit antimikrobiellen Richtlinien für die Staphylokokken Pyodermie auszustatten, inklusive jener mit Methicillin‐resistenten Staphylokokken. Eine Anleitung zur Diagnose von Oberflächen‐, oberflächlicher und tiefer Pyodermie, sowie die zugrundeliegenden Primärursachen sind inkludiert. Die Empfehlungen zielen darauf ab, die Behandlungserfolge zu optimieren, während ein verantwortungsbewusster Einsatz von Antibiotika erfolgt.

**Materialien und Methoden:**

Die Evidenz wurde aus einer systematischen Literatur Review aus Behandlungsstudien in englischer Sprache für canine Pyodermie bis zum 23/12/2023 zusammengesucht. Die Qualität wurde mittels SORT‐Kriterien erfasst und mit der Konsensus Evaluierung der Autoren kombiniert. Über die Empfehlungen wurde in einem schrittweisen Prozess abgestimmt, gefolgt von einem Delphi‐Stil Feedback Prozess vor einer endgültigen Übereinstimmung durch die Autoren.

**Ergebnisse:**

Eine Zytologie sollte in allen Fällen, bei denen Antibiotika eingesetzt werden, durchgeführt werden. Eine alleinige topische Therapie ist die Behandlung der Wahl für Oberflächen‐ und oberflächliche Pyodermie. Systemische Antibiotika sollten für tiefe Pyodermien reserviert werden oder für oberflächliche Pyodermien, bei denen die topische Therapie alleine nicht wirksam ist. Eine systemische Therapie mit zusätzlicher topischer Therapie wird anfangs für 2 Wochen bei oberflächlicher und 3 Wochen bei tiefer Pyodermie eingesetzt, gefolgt von einer neuerlichen Untersuchung, um den Fortschritt zu erfassen und primäre Fälle zu managen. Mittel der ersten Wahl haben erwartungsgemäß eine Wirksamkeit gegenüber der Mehrheit der Methicillin‐empfindlichen *Staphylococcus pseudintermedius*; für alle anderen sollte ein Labortest die Empfindlichkeit bestätigen und die Einsatzmöglichkeit sicherer Alternativen dadurch ausgeschlossen sein. Da Bakterienkultur und Antibiogramm essenziell sind, um eine systemische Therapie zu vertreten, sollten diese von Labors und Praxen zu vernünftigen Preisen angeboten werden, um ihren Einsatz zu unterstützen. Eine proaktive topische Therapie mittels Antiseptika könnte helfen, ein Wiederauftreten zu verhindern.

**Schlussfolgerungen und klinische Bedeutung:**

Der einfache Zugang zur Haut ermöglicht exzellente, erreichbare Möglichkeiten für antimikrobielle Stewardship.

## 摘要

**背景:**

犬脓皮病是小动物临床中最常见的病症之一，经常导致抗菌药物的处方使用。

**目的:**

为临床医生提供犬葡萄球菌性脓皮病(包括耐甲氧西林葡萄球菌感染)的抗菌治疗指南。指南涵盖了表面、浅表和深层脓皮病的诊断及其潜在的原发性病因。推荐意见旨在优化治疗效果的同时，促进负责任的抗菌药物使用。

**材料与方法:**

通过系统回顾截至2023年12月23日英文文献中关于犬脓皮病治疗的研究来收集证据。质量评估采用SORT标准，并结合作者共识进行评价。推荐意见通过反复投票以及德尔菲式反馈流程完成，最终由作者达成一致。

**结果:**

在使用抗菌药物之前，应对所有病例进行细胞学检查。对于表面和浅表脓皮病，单独使用外部抗菌治疗是首选治疗方法。全身性抗菌药物应仅用于深层脓皮病以及浅表脓皮病经外部治疗无效时。对于浅表脓皮病，全身性治疗联合外部治疗应先持续2周，对于深层脓皮病则应持续 3 周，之后重新检查以评估疗效并管理原发因素。首选药物通常可对大多数对甲氧西林敏感的假中间型葡萄球菌有效；对于其他情况，应通过实验室检测确认药物敏感性，并排除更安全的替代药物。由于培养和药敏试验对于合理使用全身性抗菌药物至关重要，实验室和诊所应合理定价以鼓励使用。主动使用含有抗菌成分的外用制剂可能有助于预防复发。

**结论与临床意义:**

皮肤的易接近性为抗菌药物管理提供了极好的、可实现的机会。

## Résumé

**Contexte:**

La pyodermite canine est l'une des présentations les plus courantes dans la pratique des petits animaux, conduisant fréquemment à la prescription d'antimicrobiens

**Objectifs:**

Fournir aux cliniciens des lignes directrices pour le traitement antimicrobien des pyodermites staphylococciques, y compris celles impliquant des staphylocoques résistants à la méticilline. Des conseils sur le diagnostic des pyodermites de surface, superficielles et profondes, ainsi que sur leurs causes primaires sous‐jacentes, sont inclus. Les recommandations visent à optimiser les résultats du traitement tout en promouvant une utilisation responsable des antimicrobiens.

**Matériels et méthodes:**

Les données ont été recueillies à partir d'une analyse documentaire systématique des études en langue anglaise sur le traitement de la pyodermite canine jusqu'au 23/12/2023. La qualité a été évaluée à l'aide des critères SORT et combinée à l'évaluation consensuelle des auteurs. Les recommandations ont été votées dans le cadre d'un processus itératif, suivi d'un processus de rétroaction de type Delphi avant l'accord final des auteurs.

**Résultats:**

Une cytologie doit être effectuée dans tous les cas avant d'utiliser des antimicrobiens. La thérapie antimicrobienne topique seule est le traitement de choix pour les pyodermites de surface et superficielles. Les antimicrobiens systémiques doivent être réservés aux pyodermites profondes et aux pyodermites superficielles lorsque le traitement topique n'est pas efficace. Le traitement systémique, accompagné d'un traitement topique complémentaire, est administré initialement pendant deux semaines pour les pyodermites superficielles et trois semaines pour les pyodermites profondes, suivi d'un réexamen pour évaluer l'évolution et traiter les causes primaires. Les médicaments de premier choix ont une efficacité attendue contre la majorité des *Staphylococcus pseudintermedius* sensibles à la méticilline*;* pour tous les autres, les tests de laboratoire doivent confirmer la sensibilité et exclure la pertinence d'alternatives plus sûres. La culture et l'antibiogramme étant essentiels pour rationaliser le traitement systémique, les laboratoires et les cabinets médicaux doivent les facturer à un prix raisonnable afin d'encourager leur utilisation. Un traitement topique proactif utilisant des antiseptiques peut aider à prévenir les récidives.

**Conclusions et pertinence clinique:**

L'accessibilité de la peau offre d'excellentes possibilités, réalisables, de gestion des antimicrobiens.

## 要約

**背景:**

犬の膿皮症は小動物臨床において最も一般的な疾患の一つであり、しばしば抗菌薬の処方につながる。

**目的:**

本報告の目的は、メチシリン耐性ブドウ球菌を含むブドウ球菌性膿皮症に対する抗菌薬治療のガイドラインを臨床医に提供することである。表面性膿皮症、表在性膿皮症、深在性膿皮症、およびそれらの根本的な主原因の診断に関するガイダンスを含む。推奨事項は、責任ある抗菌薬使用を促進しながら治療成績を最適化することを目的としている。

**材料と方法:**

2023年12月23日までの犬の膿皮症に関する英語による治療研究の系統的文献レビューからエビデンスを収集した。SORT基準を用いて質を評価し、著者のコンセンサス評価と組み合わせた。推奨は、著者による最終的な合意の前に、デルフィ式フィードバックプロセスを経て、反復プロセスで投票された。

**結果:**

抗菌薬を使用する前に、すべての症例で細胞診を行うべきである。表面および表在性の膿皮症に対しては、局所抗菌薬療法のみを選択すべきである。抗菌薬の全身投与は、深在性膿皮症および局所療法が無効な場合の表在性膿皮症にのみ行う。全身療法と補助的な局所療法は、まず表在性膿皮症では2週間、深在性膿皮症では3週間行い、その後、経過を評価し、主原因を管理するために再検査を行う。第一選択薬は、メチシリン感受性*Staphylococcus pseudintermedius*の大部分に対して有効である。細菌培養検査および薬剤感受性検査は全身療法の合理化に不可欠であるため、検査施設および診療所は適正な価格を設定して使用を奨励すべきである。殺菌薬を用いた積極的な局所療法は再発予防に役立つであろう。

**結論と臨床的意義:**

皮膚へのアクセスが容易であるため、抗菌薬管理に対する、優れた、達成可能な機会を提供する。

## Resumo

**Contexto:**

A piodermite canina é uma das apresentações mais comuns na clínica de pequenos animais, frequentemente levando à prescrição de antimicrobianos.

**Objetivos:**

Fornecer aos clínicos diretrizes de tratamento antimicrobiano para piodermite estafilocócica, incluindo aquelas envolvendo estafilococos resistentes à meticilina. Orientações sobre o diagnóstico de piodermite de superfície, profunda e superficial, e suas causas primárias subjacentes, estão incluídas. As recomendações visam otimizar os resultados do tratamento, ao mesmo tempo em que promovem o uso responsável de antimicrobianos.

**Materiais e Métodos:**

As evidências foram coletadas de uma revisão sistemática da literatura de estudos de tratamento para piodermite canina escritos em inglês até 23/12/2023. A qualidade foi avaliada utilizando os critérios SORT e combinada com a avaliação de consenso dos autores. As recomendações foram votadas em um processo iterativo, seguido por um processo de *feedback* no estilo Delphi antes do acordo final pelos autores.

**Resultados:**

A citologia deve ser realizada em todos os casos antes que os antimicrobianos sejam utilizados. A terapia antimicrobiana tópica sozinha é o tratamento de escolha para piodermites de superfície e superficiais. Antimicrobianos sistêmicos devem ser reservados para piodermites profundas e para piodermites superficiais quando a terapia tópica não for eficaz. A terapia sistêmica, com tratamento tópico adjuvante, é inicialmente fornecida por 2 semanas em piodermites superficiais e 3 semanas em piodermites profundas, seguida de reexame para avaliar o progresso e controlar as causas primárias. Os medicamentos de primeira escolha têm eficácia esperada contra a maioria dos *Staphylococcus pseudintermedius* suscetíveis à meticilina; para todos os outros, os testes laboratoriais devem confirmar a suscetibilidade e excluir a possibilidade de uso de alternativas mais seguras. Como os testes de cultura e suscetibilidade são essenciais para o uso racional da terapia sistêmica, os laboratórios e as clínicas devem precificá‐los razoavelmente para incentivar o seu uso. A terapia tópica proativa usando antissépticos pode ajudar a prevenir recorrências.

**Conclusões e relevância clínica:**

A acessibilidade da pele oferece excelentes oportunidades viáveis para a administração antimicrobiana.

## RESUMEN

**Introducción:**

La pioderma canina es una de las presentaciones más comunes en la práctica clínica de pequeños animales, lo que frecuentemente conlleva la prescripción de antimicrobianos.

**Objetivos:**

Proporcionar a los profesionales clínicos pautas de tratamiento antimicrobiano para la pioderma estafilocócica, incluyendo aquellas que involucran estafilococos resistentes a la meticilina. Se incluye orientación sobre el diagnóstico de pioderma externa, superficial y profunda, y sus causas primarias subyacentes. Las recomendaciones buscan optimizar los resultados del tratamiento, promoviendo al mismo tiempo el uso responsible de antimicrobianos.

**Materiales y métodos:**

La evidencia se obtuvo de una revisión sistemática de la literatura sobre estudios de tratamiento de pioderma canina en inglés hasta el 23/12/2023. La calidad se evaluó mediante los criterios SORT y se combinó con la evaluación por consenso de los autores. Las recomendaciones se votaron mediante un proceso iterativo, seguido de un proceso de retroalimentación tipo Delphi antes de la aprobación final por parte de los autores.

**Resultados:**

Se debe realizar una citología en todos los casos antes de utilizar antimicrobianos. La terapia antimicrobiana tópica por sí sola es el tratamiento de elección para las piodermas externas y superficiales. Los antimicrobianos sistémicos deben reservarse para las piodermas profundas y para las piodermas superficiales cuando la terapia tópica no es efectiva. La terapia sistémica, con tratamiento tópico adyuvante, se administra inicialmente durante 2 semanas en las piodermas superficiales y 3 semanas en las profundas, seguida de una reevaluación para evaluar la evolución y abordar las causas primarias. Los fármacos de primera elección tienen la eficacia esperada contra la mayoría de los *Staphylococcus pseudintermedius* sensibles a la meticilina; para todos los demás, las pruebas de laboratorio deben confirmar la sensibilidad y descartar la idoneidad de alternativas más seguras. Dado que el cultivo y las pruebas de sensibilidad son esenciales para racionalizar la terapia sistémica, los laboratorios y las clínicas deben fijar un precio razonable para fomentar su uso. La terapia tópica proactiva con antisépticos puede ayudar a prevenir las recurrencias.

**Conclusiones y relevancia clínica:**

La accesibilidad de la piel ofrece excelentes oportunidades para la optimización del uso de antimicrobianos.

**Abbreviations**
ALD: acral lick dermatitis
BAN: British approved name
BC/AST: bacterial culture and antimicrobial susceptibility testing
BOG: bacterial overgrowth
CLSI: Clinical & Laboratory Standards Institute
DLE: discoid lupus erythematosus
DP: deep pyoderma
EMA: European Medicines Agency
ExP: exfoliative superficial pyoderma
FDA: U.S. Food & Drug Administration
FOI: freedom of information
GSP: German shepherd dog pyoderma
i.m.: intramuscularly
INN: International Nonproprietary Names
ISCAID: International Society for Companion Animal Infectious Diseases
i.v.: intravenously
LoE: level of evidence
MCLE: mucocutaneous lupus erythematosus
MDR: multidrug‐resistant
MSSP: meticillin‐susceptible *Staphylococcus pseudintermedius*
MRS: meticillin‐susceptible *staphylococci*
MRSA: meticillin‐susceptible *Staphylococcus aureus*
MRSC: meticillin‐susceptible *Staphylococcus coagulans*
MRSP: meticillin‐susceptible *Staphylococcus pseudintermedius*
PK‐PD: pharmacokinetics‐pharmacodynamics
PO: per os
q: every (Latin: quaque)
RCT: randomised controlled trial
SBF: superficial bacterial folliculitis
SC: subcutaneously
SOR: strength of recommendation
SORT: strength of recommendation taxonomy
SYREAF: Systematic Reviews for Animals & Food
TMPS: trimethoprim‐sulfonamide
USAN: United States Adopted Names
VCLE: vesicular cutaneous lupus erythematosus
WAVD: World Association for Veterinary Dermatology

1

**Table:** 

**Abstract**	**234**
**1 Background**	**236**
What is included?	236
Methods	236
How to use the guidelines	237
**2 Types of canine pyoderma**	**237**
Differentiating depth of infection	237
Bacterial pathogens typically involved in canine pyoderma	237
**3 Diagnostic approach and diagnostic tests**	**238**
Skin examination	238
Cytological examination	239
Investigations into underlying primary causes	240
Bacterial culture and antimicrobial susceptibility testing (BC/AST)	241
How to take representative skin samples	242
**4 Surface pyoderma**	**243**
Clinical presentations	243
Diagnosis	244
Treatment recommendations for surface pyoderma	245
**5 Superficial pyoderma**	**247**
Clinical presentations	247
Diagnosis	250
Treatment recommendations for superficial bacterial folliculitis (SBF), impetigo and exfoliative superficial pyoderma	250
Treatment recommendations for mucocutaneous pyoderma	253
**6 Deep pyoderma**	**253**
Clinical presentations	253
Diagnosis	256
Treatment recommendations for deep pyoderma	256
Clinical case scenario	259
**7 Topical antimicrobial therapy**	**260**
General comments	260
Which active ingredient?	260
Product formulations	263
Practical tips to promote good outcomes	263
**8 Systemic antimicrobial therapy**	**264**
General comments	264
Culture‐based treatment	264
Empirical choices	265
First‐choice drugs	265
Second‐choice drugs	265
Reserved antimicrobial drugs	266
Strongly discouraged antimicrobial drugs	268
**9 Preventing recurrences of pyoderma**	**270**
Undiagnosed underlying causes	270
Recurrent pyoderma in atopic dogs	271
What if recurrences keep happening?	271
Current or future alternative treatment options	271
**10 Meticillin‐resistant staphylococcal pyoderma**	**271**
General comments	271
Treatment of MRS infection	272
**Conclusions**	**273**
**References**	273

## 2 BACKGROUND

Pyoderma (bacterial skin infection) is common in dogs and is among the top four presentations that lead to antimicrobial prescribing.^1^ Given the urgent need to reduce inappropriate use of antimicrobials and limit the spread of antimicrobial resistance among human and animal pathogens, the skin offers clear opportunities for antimicrobial stewardship owing to its accessibility for diagnosis and topical antimicrobial treatment. These guidelines aim to provide clinicians with practical treatment recommendations for managing canine pyoderma, while at the same time promoting rational use of antimicrobials. Another objective was to revise outdated recommendations regarding treatment duration, choice and dosing of antimicrobial agents.

### What is included?

The guidelines deal with canine bacterial skin infections, primarily involving *Staphylococcus pseudintermedius* and other staphylococci, both meticillin‐susceptible and meticillin‐resistant. For information on wounds or abscesses and less common skin infections involving organisms such as *Mycobacterium* spp., *Mycoplasma* spp., *Nocardia* spp. or *Dermatophilus congolensis*, readers are referred to other sources.^2^, ^3^, ^4^

The document includes an update of the superficial bacterial folliculitis guidelines previously published on behalf of the International Society for Companion Animal Infectious Diseases (ISCAID)^5^ based on recent advances in the field. It also adds new recommendations for the diagnosis and antimicrobial therapy of surface and deep pyoderma in dogs and provides a brief overview specific to meticillin‐resistant staphylococci (MRS), expanding on previous MRS guidelines.^6^ Based on recent microbiological and immunological insights, the term ‘surface pyoderma’ has become controversial and may be more appropriately replaced with ‘dysbiosis’ (see Section ‘Clinical presentations’). However, the clinical presentations known as surface pyodermas remain included here in order to highlight the best treatment options.

### Methods

A major challenge for companion animal antimicrobial guidelines is the paucity of objective data, particularly from adequately designed and implemented randomised controlled trials.^7^, ^8^ As with the previous guidelines,^5^ and those pertaining to other diseases,^9^, ^10^ recommendations are based on data from published studies of varying strengths combined with general principles of infectious disease, antibacterial agent pharmacology, and consensus from an author panel with international clinical expertise in veterinary dermatology, internal medicine, microbiology and pharmacology.

Recommendations were developed using an iterative process during online meetings. Each author contributed to different parts of the document, yet all reviewed the entire document and voted on all recommendations. Recommendations that did not receive 100% approval were discussed and revised further; final consensus for a recommendation was unanimous from all authors unless stated otherwise.

Systematic literature searches were conducted for clinical treatment studies of dogs with pyoderma; publications meeting the search criteria are listed in the Tables [Link], [Link] in the Supporting information. Conference abstracts that were not available as peer‐reviewed full publications were excluded. The full methods and search strategy, including search terms, were deposited for public access within the SYREAF (Systematic reviews for animals and food) site.^11^ Databases were searched on 22 April 2022 and updated on 23 December 2023.^12^

The quality of evidence from published therapeutic trials was included in our treatment recommendations where appropriate using the Strength of Recommendation Taxonomy (SORT)^13^ as a system utilised in veterinary publications relating to skin disease (Table 1).^14^, ^15^

**TABLE 1:** Descriptors for level of evidence and strength of recommendation categories used based on the Strength of Recommendation Taxonomy (SORT) (modified from Ebell et al.^13^).

Level of evidence (LoE)		Definition for treatment studies
1	Good quality, patient orientated	‘High‐quality’ randomised controlled trial (RCT) *or* meta‐analysis of consistent RCTs with ≥10 dogs per group
2	Limited quality patient‐orientated	‘Low‐quality’ RCT downgraded owing either to <10 dogs per group, lack of separate assessment of groups, lack of specific clinical interpretation *or* prospective case series (cohort study) containing ≥10 dogs
3	Other evidence	Prospective case series containing <10 dogs *or* a retrospective case series (any size)

Strength of recommendation (SOR)		Definition
A	Strong	Based on good‐quality patient‐orientated evidence (one or more LoE 1 studies)
B	Moderate	Based on limited‐quality patient‐orientated evidence (one or more LoE 2 studies)
C	Weak	Based on consensus combined with disease‐orientated evidence or case series (LoE 3)

Following this internal process, 15 veterinarians, including general practitioners and specialists from relevant disciplines, from different continents, with an interest in skin disease and antimicrobial stewardship, were invited to critique the draft document. Their comments were evaluated and any modifications to recommendations were voted upon using a Delphi‐style consensus before submission for publication.

### How to use the guidelines

The guidelines should be interpreted as general recommendations appropriate for good clinical management in the majority of cases. They should be used as a basis for case‐specific decision‐making by the attending veterinarian, taking into account the dog's general health status and overall circumstances. Furthermore, this document is intended for an international readership. It remains the responsibility of any veterinarian prescribing antimicrobials to be familiar with their regional antimicrobial susceptibility trends, availability of antimicrobial drugs and national prescribing regulations applicable to antimicrobials. Additionally, the guidelines can be used by national veterinary organisations to develop or update national guidelines.

Given that bacterial skin infection always occurs secondary to a primary underlying cause, it is incumbent upon attending veterinarians to identify and address the primary aetiology to achieve resolution of an infection and avoid recurrence; this may require consideration of multiple recommendations beyond those for antimicrobial treatment.

## 3 TYPES OF CANINE PYODERMA AND BACTERIAL PATHOGENS

**FIGURE 1:**
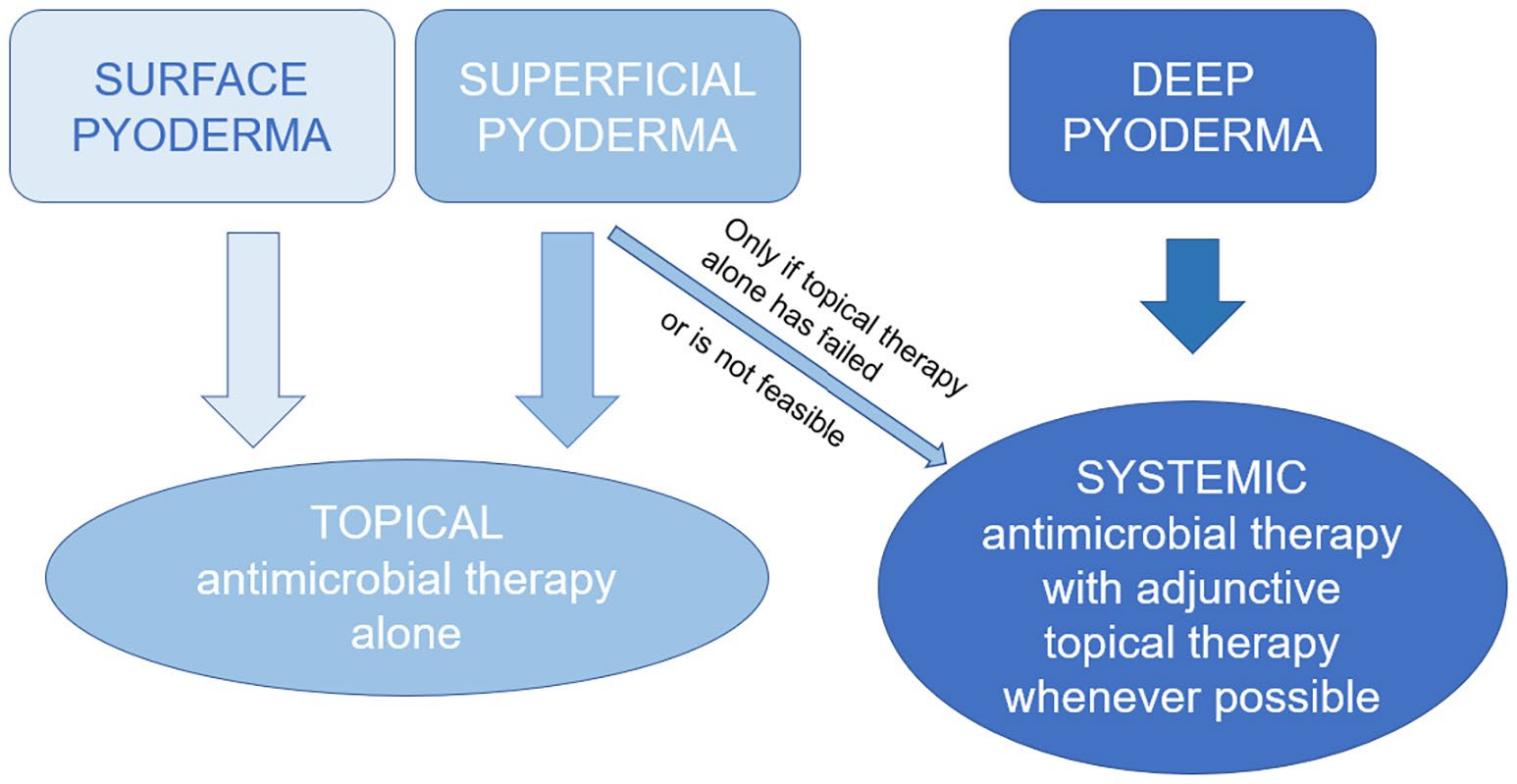
Histopathological classification of canine pyoderma by depth, and recommended treatment approaches (topical antimicrobial therapy including antiseptics and topical antibiotics).

Bacterial skin infections present in many different clinical forms, and a varied, inconsistent terminology for the different presentations has evolved. Within these guidelines, we have adopted the classification by histological depth of infection, which distinguishes surface, superficial and deep pyoderma.^16^ In contrast to a problem‐based classification of pyoderma, which has been proposed to help in diagnosis,^17^, ^18^ the classification by depth is most helpful in choosing the most appropriate treatment approach (Figure 1).

### Differentiating depth of infection

Surface, superficial and deep pyoderma are differentiated through a combination of lesion types and location on the body. No single lesion is pathognomonic for a specific depth of pyoderma (Table 2).

**TABLE 2:** Commonly recognised presentations of canine pyoderma by depth of infection as used in these guidelines and examples of typical lesions and findings (more details given in Tables 5, 7 and 9).

Depth/type of pyoderma	Clinical presentations	Most common lesions and frequent clinical findings
Surface	Pyotraumatic acute moist dermatitis (‘hot spot’) Intertrigo (skin‐fold dermatitis) Bacterial overgrowth syndrome (see Section ‘Clinical presentations’ for controversy over inclusion as an infection)	Erythema, exudation, alopecia, pruritus Erythema, malodour Erythema, surface exudate
Superficial	Superficial bacterial folliculitis (SBF) Exfoliative superficial (spreading) pyoderma Impetigo Mucocutaneous pyoderma	Papules, follicular pustules, epidermal collarettes, crusts Epidermal collarettes, scales, crust Papules, interfollicular pustules, epidermal collarettes Erythema, crusting, hypopigmentation
Deep	Widespread furunculosis/cellulitis Localised furunculosis/cellulitis, for example: Pyotraumatic folliculitis and furunculosis Acral lick dermatitis Infected interdigital nodules Callus pyoderma Chin pyoderma/chin ‘acne’ Postgrooming furunculosis	Can be present in any presentation of deep pyoderma: Haemorrhagic crusts, haemopurulent discharge, fistulae (sinuses), ulcers, nodules, plaque, ill‐defined swelling Pain, lymphadenomegaly

Differentiation based on clinical signs may be challenging in some cases,^19^ especially because not all pyoderma types have been clearly defined yet. For example, pyotraumatic dermatitis (surface pyoderma) can closely resemble pyotraumatic folliculitis/furunculosis (deep pyoderma) and controversy remains whether these are distinct diseases or a continuum with different severity.^20^ Likewise, the lesions associated with lip‐fold intertrigo (surface pyoderma), mucocutaneous pyoderma (superficial pyoderma) and mucocutaneous lupus erythematosus (sterile immune‐mediated disease) can closely resemble each other or overlap.^21^ Until a better understanding of these conditions is available, treatment decisions need to be made on a case‐by‐case basis and within the wider clinical context. A stepwise approach, which addresses the infectious component with topical antimicrobial therapy first, followed by re‐assessment, will be prudent and effective in many cases. Skin biopsy for histopathological evaluation may need to be considered if lesions remain and if other differential diagnoses need to be investigated.

### Bacterial pathogens typically involved in canine pyoderma

Staphylococci are the predominant bacterial pathogens in canine pyoderma. According to a retrospective study from Canada, staphylococci accounted for 76.8% of bacterial isolates from canine skin samples between 1994 and 2013, followed by streptococci (8.7%), *Pseudomonas aeruginosa* (4.4%) and *Escherichia coli* (4.2%).^22^ Recent studies based on 16S ribosomal RNA gene sequencing have confirmed that staphylococci are the most abundant bacteria in swabs collected from skin lesions in atopic dogs.^23^

Among the staphylococci, the coagulase‐positive *S. pseudintermedius* is found in >90% of all types of canine pyoderma, with even higher percentages from superficial pyoderma based on data from clinical studies and from laboratory submissions, irrespective of its meticillin‐susceptibility status.^6^, ^24^ Less information is available for surface pyoderma, where typically a more mixed microbial population, including other Gram‐positive bacteria (e.g. other staphylococci, enterococci, streptococci), Gram‐negative bacteria (e.g. *Pseudomonas* spp.) and yeasts (e.g. *Malassezia pachydermatis*) can be expected.^25^, ^26^ For deep pyoderma, our literature search in 2022 identified *S. pseudintermedius* as the main pathogen, with streptococci, Gram‐negative bacteria, and anaerobes found in approximately 40% of cases, highlighting the importance of bacterial culture and susceptibility testing in deep infections (Table 10).

Other coagulase‐positive staphylococci such as *S. aureus* and *S. coagulans* (formerly *S. schleiferi* subsp. *coagulans*) can colonise and infect dogs, yet typically account for <10% of laboratory submissions from canine pyoderma.^27^, ^28^, ^29^ *S. schleiferi* was originally divided into two subspecies based on their coagulase status: *S. schleiferi* subsp. *schleiferi* as the coagulase‐negative variant and *S. schleiferi* subsp. *coagulans* as the coagulase‐positive variant.^30^ They gained relevance owing to high rates of meticillin resistance among clinical isolates.^31^, ^32^ The subspecies were previously shown to be genotypically indistinct by commonly employed diagnostic methods, and not to differ in biological behaviour or pathogenic potential.^31^, ^33^, ^34^ Reclassification of *S. schleiferi* subspecies as separate species—the coagulase‐negative *S. schleiferi* and the coagulase‐positive *S. coagulans—*has been recommended based on genomic analysis.^34^, ^35^ Studies have suggested that *S. schleiferi* is more commonly isolated from humans, while *S. coagulans* is more commonly isolated from dogs.^34^, ^36^ Additional studies utilising larger numbers of isolates from wider geographical regions are necessary to confirm this proposed host affinity. Given the difficulty in distinguishing between the subspecies using common methods, and the lack of uniform adoption of the new species classification by diagnostic laboratories, this guideline document will employ *S. coagulans* to refer to both species. Both *S. coagulans* and *S. schleiferi* should be considered as potential pathogens when obtained from a properly collected skin culture sample.

## 4 DIAGNOSTIC APPROACH AND TESTS

**FIGURE 2:**
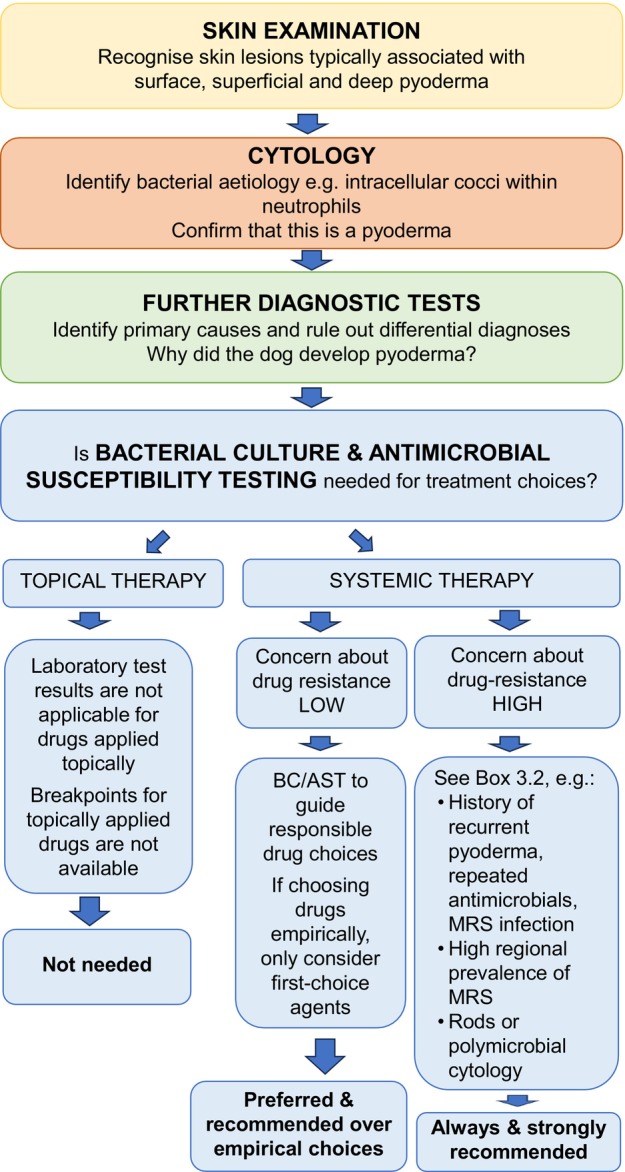
Stepwise diagnostic approach to a dog with pyoderma.

Routinely adopting a three‐step approach to every case of suspected pyoderma before prescribing antimicrobial treatment can reduce inappropriate use of antimicrobials, support a speedier successful treatment outcome and help to avoid relapses (Figure 2).

### Skin examination

The skin is uniquely accessible for inspection and sampling. It should be examined in its entirety to determine the extent and distribution of skin lesions. To accurately assess lesion types, long hair may need to be trimmed in affected areas; scissors are often preferred over clippers to avoid damage to lesions before sampling.

### Cytology

Cytology from affected skin should be performed in every case of suspected canine pyoderma to confirm a bacterial cause.

Cytological evaluation of skin lesions is a key tool in practicing responsible antimicrobial stewardship and one of the most valuable tests in the diagnosis, management and follow‐up of dogs with skin diseases. The diagnostic sensitivity of cytology results has been assessed as 93% in canine superficial pyoderma, based on the presence of intracellular (phagocytosed) cocci and neutrophils.^37^

Many skin lesions associated with pyoderma also occur with noninfectious disease, and it is therefore essential to confirm a bacterial cause before choosing antibacterial therapy. For example, pustules—the hallmark lesions for canine SBF—are also seen as the early lesions in pemphigus foliaceus, which is a sterile immune‐mediated disease. Likewise, papules and erythema are recognised as common lesions in canine atopic dermatitis.^38^

Microscopic examination of skin samples for cells (cytology) can be readily achieved in‐house. A rapid staining kit and a microscope with a bright light source, usually a × 100 objective lens, and ×10 ocular lens for ×1000 magnification of host cells, bacteria and fungi will be needed, similar to that required to examine blood smears.^39^ Cytology should be part of every skin examination and, luckily, is achievable in any practice set‐up, with very little time or funds required, and yields high‐value information (Box 1).

BOX 1 **Benefits of skin cytology**

1 Can be performed in‐house with immediate results.

Rapid staining methods such as a modified Wright–Giemsa stain (e.g. Diff‐Quik) are adequate; they do not require Gram stains or other complicated stains.

Can confirm the diagnosis of pyoderma and the need for topical or systemic antimicrobial therapy (typically finding cocci and/or rods within neutrophils).

Can differentiate pyoderma from diseases with similar clinical signs yet different aetiologies (e.g. *Malassezia* dermatitis, sterile granulomatous skin diseases, pemphigus foliaceus).

Is needed to interpret bacterial culture results by confirming that relevant organisms have been cultured, particularly when multiple pathogens are reported.

Can be used to monitor response to treatment and confirm resolution of infection.

**FIGURE 3:**
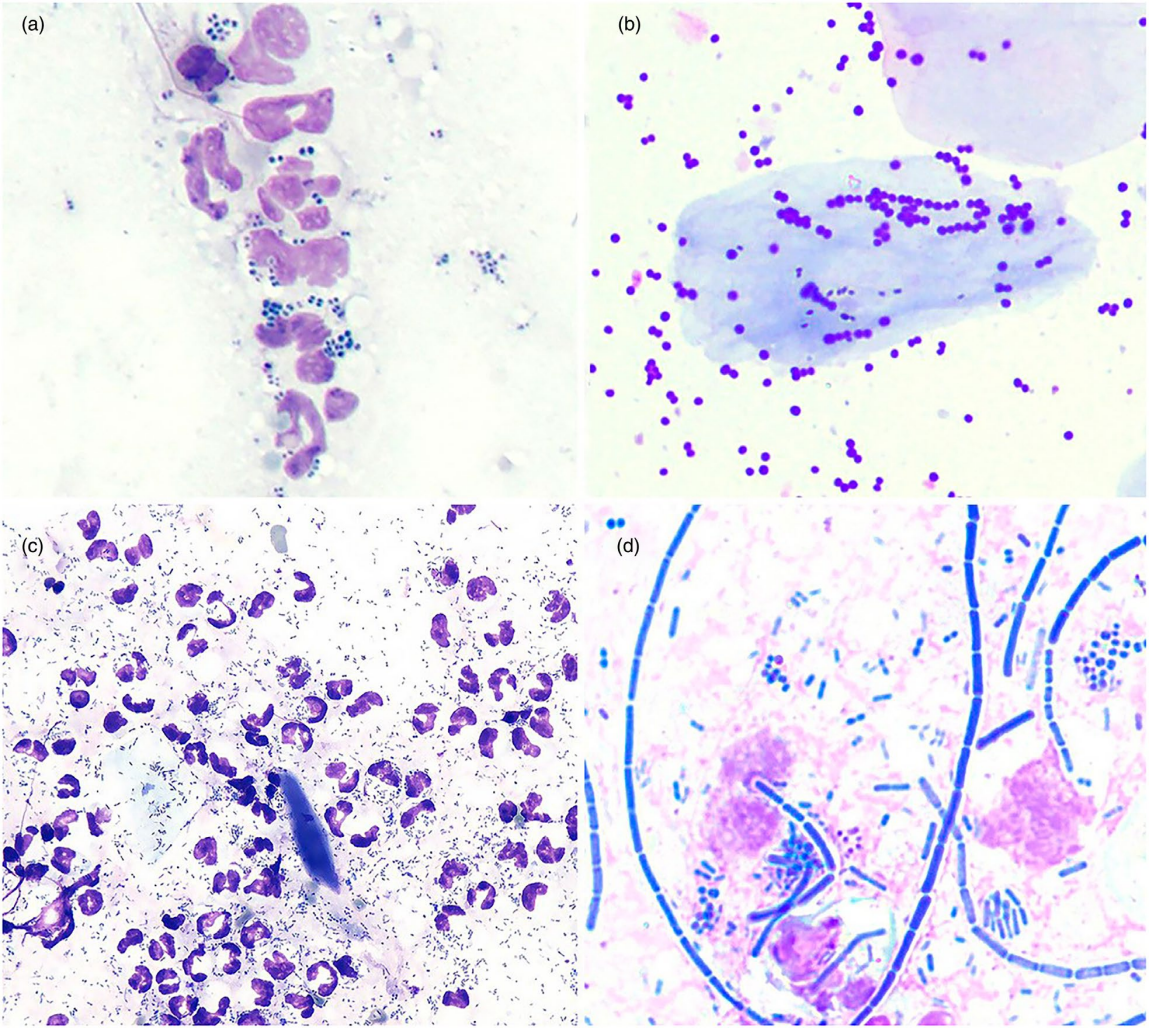
Examples of typical cytology findings from canine pyoderma. Samples taken from skin lesions and stained with modified Wright‐Giemsa stain (e.g. Diff‐Quick). ×1000. (a) Cocci, most likely staphylococci, some dividing into pairs and quadruplets, in and among neutrophils; impression smear of pus expressed from a pustule. (b) Large numbers of cocci without neutrophils from a dog with bacterial overgrowth syndrome; acetate tape sample from skin on the abdomen. (c) Large numbers of rods (bacilli) among degenerate neutrophils (some with nuclear streaming or streaking); impression smear of pus from a moist lesion on the neck (image courtesy S. Shaw). (d) Mixed microbial morphologies (rods and cocci); impression smear from a dog's lip fold.

Cytology samples are first assessed qualitatively for the presence of bacteria, their morphologies (cocci, rods), and for the presence of inflammatory cells. Samples from staphylococcal pyoderma lesions will typically show intracellular cocci (within neutrophils or macrophages, with or without some keratinocytes (squames or corneocytes; Figure 3)). Secondly, assessing the abundance of bacteria may point towards their relevance. Both semiquantitative and quantitative methods for evaluation of skin cytology have been described.^37^, ^40^ Individual bacteria in low numbers may be identified in samples from healthy skin depending on sample site. Thresholds for numbers of bacteria required to define infection have not been validated. Our group agreed that the following findings are supportive of pyoderma:

- intracellular bacteria *or*
- extracellular bacteria with nuclear streaking (also called streaming) *or*
- as in the case of bacterial overgrowth (see Section ‘Surface pyoderma’), large numbers of bacteria from lesional skin in the absence of inflammatory cells

However, with a sensitivity of 93% for superficial pyoderma, the absence of cocci does not entirely exclude a diagnosis. In cases of deep pyoderma, the absence or presence of bacteria from surface samples has little relevance for diagnosis. Recognition of other morphologies (rods [bacilli]; *Malassezia* yeast) or of mixed populations is important for treatment decisions (e.g. in determining the need for concurrent antifungal therapy). Estimating the predominant bacterial morphologies is also crucial to guide meaningful interpretation of bacterial culture and antimicrobial susceptibility testing (BC/AST) results (see Section ‘Bacterial culture and antimicrobial susceptibility testing (BC/AST)’).

### Investigations into underlying primary causes

Pyoderma is always secondary to underlying primary causes, and these must be considered at the first occurrence.

All dogs are thought to be colonised by staphylococci, but most do not develop pyoderma. Infection arises when the skin barrier is altered by predisposing factors. Although the pathogenesis of pyoderma is not fully understood, microbiological and molecular studies have shown that *S. pseudintermedius* isolates involved in skin infections are indistinguishable and likely originate from commensal *S. pseudintermedius* populations.^41^, ^42^ A primary underlying cause is always required to trigger or facilitate bacterial skin disease, even if such a cause is not immediately apparent. The concept of ‘idiopathic’ or ‘primary’ pyoderma is no longer tenable.

Trauma (e.g. abrasions, cuts, friction and chronic pressure) is probably the most common underlying primary cause that leads to bacterial skin infection, yet ectoparasitic infestation and atopic skin disease are likely to be the most troublesome in small animal practice, accounting for 80% of cases in one retrospective study.^43^ Additionally, altered cornification and/or systemic diseases such as endocrinopathies and neoplasia can lead to structural, immunological or chemical changes in the skin and, consequently, secondary pyoderma (Table 3).

**TABLE 3:** Typical underlying causes and commonly associated types of canine pyoderma.

Disease group	Disease	Most common type of pyoderma
Allergic skin disease	Atopic dermatitis	SBF, ExP Pyotraumatic dermatitis particularly frequent with flea bite hypersensitivity
	Adverse food reaction	
	Flea bite hypersensitivity	
Ectoparasitic diseases	Sarcoptic mange	SBF, rarely localised DP
	Demodicosis	DP, SBF
	Cheyletiellosis	SBF, ExP
Infectious diseases	Dermatophytosis	SBF, DP
	Leishmaniosis	DP, SBF
Endocrine disorders	Hypothyroidism	SBF, ExP, rarely DP
	Hyperadrenocorticism	
	Sex hormone imbalances	SBF, ExP
Autoimmune diseases	Pemphigus foliaceus	SBF, ExP
Follicular dysplasias	Colour dilution alopecia	SBF, ExP
Keratinisation/ cornification disorders	Ichthyoses	SBF, ExP
Others	Sebaceous adenitis	SBF, ExP
	Acne	DP

Abbreviations: DP, deep pyoderma; ExP, exfoliative superficial pyoderma; SBF, superficial bacterial folliculitis.

A comprehensive overview of the diagnosis and treatment of underlying primary causes for pyoderma is beyond the scope of these guidelines, but key information can be obtained from thorough history‐taking and a comprehensive physical examination. A minimum diagnostic dataset for all dogs with pyoderma should include microscopic examination of coat brushings (for evidence of fleas or *Cheyletiella* spp.), hair plucks (trichogram) for evidence of *Demodex* spp., dermatophyte hyphae and/or fungal spores and/or skin scrapings to investigate the presence of *Sarcoptes scabiei* and *Demodex* spp. and the confirmation of ongoing appropriate acaricide and insecticide prophylactic treatment. Additional tests may include those for dermatophytosis (e.g. Wood's lamp examination, trichogram, fungal culture and PCR)^44^ and histopathological evaluation of skin biopsies for immune‐mediated and neoplastic conditions. In particular, nodular skin lesions resulting from bacterial infection need to be differentiated from infected granulomas resulting from fungi or protozoa, sterile granulomatous disease (e.g. panniculitis), neoplasia and foreign body reactions by biopsy, special stains, macerated tissue culture and, in some cases, molecular techniques.

Blood sampling for haematological, serum biochemical and, depending on results, endocrine testing and urinalysis may be indicated if an underlying cause remains elusive. Nonthyroidal illness syndrome (formerly euthyroid sick syndrome) can be associated with skin infection and can complicate the interpretation of laboratory results. Pairing T4/free T4 testing with TSH measurement is recommended to support accurate diagnosis.

### Bacterial culture and antimicrobial susceptibility testing (BC/AST)

BC/AST is recommended to guide drug choices whenever systemic therapy is planned and is preferred over empirical choices.

BC/AST is always strongly recommended when there is an increased risk of resistance to common empirically chosen antimicrobials, for example, if there is a history of recent or frequent antimicrobial use, previous isolation of meticillin‐resistant *Staphylococcus pseudintermedius* (MRSP), *S. aureus* (MRSA) or *S. coagulans* (MRSC) (formerly *S. schleiferi*) or in regions or clinics with a high prevalence of meticillin‐resistance.

BC/AST should always be paired with cytology to guide correct interpretation of laboratory results.

BC/AST is never contraindicated, but its cost is often a financial barrier. In the interest of antimicrobial stewardship and patient care, laboratories and practices should be encouraged to price these valuable tests reasonably to improve uptake, prevent treatment failure and limit inappropriate antimicrobial use.

BC/AST is not needed to make or exclude a diagnosis of pyoderma. Samples taken even from healthy skin often yield bacteria, including *S. pseudintermedius*, as part of the normal colonising microbiota in the majority of dogs (Box 2).

BOX 2 **When is BC/AST needed in a dog with surface, superficial or deep pyoderma?**

1 Surface pyoderma

- Not needed (see Section ‘Surface pyoderma’)
- Treatment‐of‐choice is always topical antimicrobial therapy alone.
- BC/AST results are not meaningful for topical agents owing to the lack of breakpoints for topical therapy.
- Cytology will provide the required information to select active ingredients.

Superficial pyoderma

- Not needed if treated with topical antimicrobial therapy alone.
- Always strongly recommended when there is concern about drug resistance:
  - History of recurrent pyoderma
  - More than one course of systemic antimicrobials within the past 6 months (for any condition, not just skin disease)
  - History of MRS infection in the affected dog or their household
  - No improvement of lesions after 5–7 days of systemic antimicrobial therapy at an appropriate dose and with good compliance.
  - Emergence of new lesions during therapy
  - High regional prevalence of MRSP (refer to local surveillance data or contact local laboratories)
  - Rod bacteria and/or polymicrobial findings on cytology

Deep pyoderma

- Always strongly recommended (see Section ‘Deep pyoderma’)

Although ‘trial therapy’ with systemic antimicrobials chosen empirically is frequently prescribed, often effective and generally safe, BC/AST‐based prescribing remains preferable to reduce the risk of treatment failure and inappropriate antimicrobial use whenever systemic therapy is needed. Our recommendation to perform BC/AST is ‘strong’ for cases with well‐recognised risk factors for MRSP (Box 2). Important risk factors are previous MRSP infection, repeated courses of systemic antimicrobials, frequent visits to veterinary clinics or having previously been seen at referral hospitals. MRSP prevalences have been reported higher from referral centres.^6^, ^45^, ^46^

In addition to guiding drug choices for systemic therapy, laboratory testing is needed to identify multidrug‐resistant (MDR) pathogens such as MRSP, MRSA and MRSC (meticillin‐resistant *S. coagulans*) so that practice infection control measures can be implemented appropriately (see Section ‘Meticillin‐resistant staphylococcal pyoderma’).^6^ Failure to recognise an infection caused by MDR bacteria can result in inappropriate antimicrobial use, a protracted clinical course and associated patient discomfort, difficulty in assessing the need for other clinical interventions required for addressing the underlying primary disease, increased client dissatisfaction and expense and a prolonged risk of transmission.^6^

The majority of MRS can be accurately identified by diagnostic laboratories based on phenotypical susceptibility testing while molecular confirmation of important marker genes (e.g. *mecA*) may be required for research purposes.^6^ By contrast, molecular antibiograms that have recently been marketed in some countries for clinical purposes need to be interpreted carefully within the clinical and microbiological context to avoid overcalling drug‐resistance.^47^

#### How to interpret BC/AST reports from dogs with pyoderma?

Samples should be submitted to laboratories that follow recognised standards for BC/AST and use breakpoints for pathogens isolated from veterinary species, currently only published by the Clinical and Laboratory Standards Institute (CLSI).^48^

With the emergence of multidrug resistance among veterinary staphylococci, accurate bacterial species identification has become critical. This is, first, because the epidemiology and owner advice vary between MRSP, MRSA and MRSC, and, secondly, because laboratory clinical breakpoints are different for *S. aureus* and *S. pseudintermedius*. Differentiation between *S. aureus* and *S. pseudintermedius* is challenging based on phenotypic and biochemical tests alone.^49^ To date, the most accurate and rapid method for bacterial identification is by matrix‐assisted laser desorption/ionisation‐time of flight (MALDI‐TOF) mass spectrometry. MALDI‐TOF has been validated for *S. pseudintermedius* identification and is now considered the method of choice in veterinary microbiology worldwide.^50^

Antimicrobial susceptibility tests need to be performed according to approved standards to allow relevant conclusions to be gained from the results. Small laboratories or in‐clinic laboratories may not have the infrastructure and microbiological expertise required to accurately perform and interpret these diagnostic tests, which can lead to inappropriate treatment choices with negative consequences for patient care.

Where multiple bacterial isolates are reported, treatment should be prescribed to target the predominant pathogen—in most cases *S. pseudintermedius*. Cytology will allow identification of the most abundant morphologies so that unnecessary use of broad‐spectrum antimicrobials to cover the reported composite susceptibility profile of multiple isolates can be avoided (see Section ‘Culture‐based treatment’).

### How to take representative skin samples

The best sampling technique will vary between lesion type (moist versus dry), expected depth of infection and purpose of sample collection (Table 4).

**TABLE 4:** Tips for sampling skin lesions for cytological evaluation and bacterial culture.

Lesion type	Comment	For cytological evaluation	For bacterial culture
Pustule (or bulla)	Pustule content optimal owing to low risk of contamination with commensals	Lance pustule with sterile needle, transfer content directly onto glass slide (impression smear)	No surface disinfection. Lance with sterile needle and transfer pus (or haemopurulent material) to sterile swab
Pus (exudative lesions)	From skin surface or expressed from draining tracts/sinuses. Remove dried surface material before sampling	Impression smear directly onto slide or use swab to sample, then roll onto slide	Squeeze and discard surface pus using a single wipe with 70% alcohol. Allow alcohol to dry, then squeeze again and sample using a sterile swab
Crust, epidermal collarette with peripheral crusting	Underside of crust or skin below is expected to yield representative bacteria	Lift crust using sterile needle, sample exposed skin with swab, slide or tape	Lift crust using sterile forceps or needle. Sample the exposed exudate or skin or beneath the ‘leading edge’ of an epidermal collarette with a sterile swab
Papule	Papule content is preferrable to surrounding skin	Pinch skin to express fluid or blood or tape strip to lift material surrounding the papule	Pinch the skin to express fluid or blood onto a sterile swab; surface sampling may be unrewarding
Dry lesions (old epidermal collarettes)	Border of epidermal collarettes preferred over centre of lesion	Tape strip to lift diagnostic material or roll/rub swab two to three times over lesion, then smear on slide	No surface disinfection. Roll sterile swab beneath the leading edge/across the border of the collarette two to three times
Erythematous skin	When no other lesions are found or when surface pyoderma is suspected (e.g. intertrigo)	Tape strip sampling or rotate swab several times over affected skin (or within a fold), then roll onto slide	Rarely needed
Deep lesions	For example, interdigital nodules or plaques	Needle aspirates or tissue impression after biopsy	Provide sedation, local or general anaesthesia as appropriate. Clip hair, clean and disinfect surface (wipe with 70% alcohol, let dry before sampling). Consider wearing gloves. Obtain tissue by biopsy (or fine needle aspirate) and submit in a sterile container or bacterial transport medium.

In surface pyoderma, most sampling techniques should provide a good yield of diagnostic material.

For superficial pyoderma, it has been shown that sampling intact pustules or skin beneath crusts or the margins of epidermal collarettes is best.^51^, ^52^, ^53^, ^54^ One study showed that multiple pustules from the same dog with SBF always harboured the same strain of *S. pseudintermedius* (as determined by pulsed‐field gel electrophoresis), while epidermal collarettes and crusts harboured multiple genotypically distinct strains of *S. pseudintermedius* with divergent antimicrobial susceptibility profiles, supporting that intact pustules should be preferentially targeted for sampling.^55^

In cases of deep pyoderma, exudative lesions (fresh pus from ulcerated skin surface, material draining from sinuses or fistulae, pustule content) are most suitable for sampling, provided that the most superficial, less representative material can be removed before sampling (Table 4). If the nidus of infection is located below the skin surface (e.g. some interdigital nodules and acral lick dermatitis lesions), a tissue biopsy is best obtained under aseptic conditions with the surface part of the skin removed so that infected tissue is examined or submitted for BC/AST (tissue culture after maceration or mincing). Where this is not possible, fine needle aspiration may be chosen. Invasive sampling will require appropriate analgesia or anaesthesia (Table 4). Surface swabs in deep pyoderma will often miss relevant pathogens, as shown in a study of dogs with acral lick dermatitis where surface culture only predicted deep tissue isolates in 8 of 22 cases.^56^

Acetate tape preparations and glass slide impression smears have a fair‐to‐good agreement for detection of bacteria, yet impression smears may be superior for detection of neutrophils.^57^ Swabs and aspirates are submitted in transport medium (typically provided with the swab) for BC/AST. Cotton swabs are suitable, yet flocked swabs, coated in short nylon fibres, placed in the provided liquid‐based collection tubes, may improve bacterial yield.^58^ When sampling draining or nodular lesions, where involvement of nonstaphylococcal, potentially zoonotic pathogens is possible, gloves should be worn and extended culture for anaerobic or fastidious bacteria or fungi should be requested.

The impact of antimicrobial therapy on bacterial culture results remains unknown yet it seems intuitive that if cocci are seen on cytology, a sample submitted for BC/AST should yield bacterial growth regardless of current treatment. However, if practical, pausing treatment for ≥2 days before sampling for culture may be prudent, even though there is no scientific evidence to underpin this recommendation.

## 5 SURFACE PYODERMA

### Clinical presentations

**FIGURE 4:**
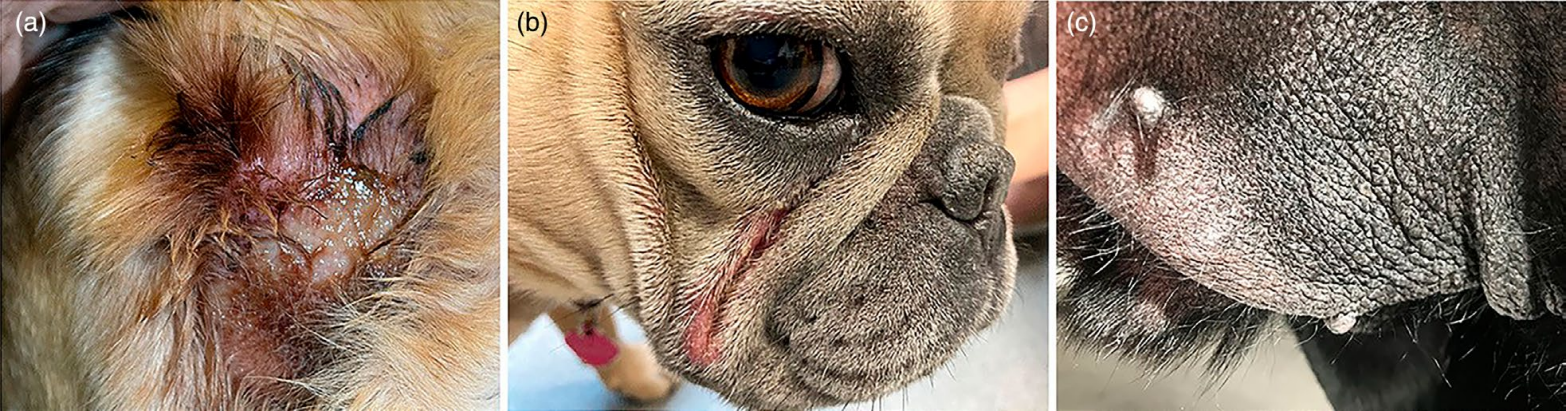
Examples of surface pyoderma in dogs. (a) Pyotraumatic dermatitis (‘hot spot’) on the lateral neck of a Newfoundland. (b) Intertrigo (skin fold dermatitis) affecting the facial folds of a bulldog. (c) Bacterial overgrowth syndrome on the palmar interdigital skin of a Labrador retriever.

Surface pyoderma encompasses three common, clinically distinct presentations where bacterial or mixed microbial overgrowth is confined to the upper layers of the epidermis. **Pyotraumatic dermatitis** (‘acute moist dermatitis, hot spot’) is characterised by a rapid onset, often within hours. It presents with an exudative, localised, pruritic or often painful lesion considered to be caused by self‐trauma promoted by pruritus, discomfort or pain affecting the area.^20^, ^59^ In **intertrigo** (fold dermatitis), moisture and skin secretions trapped in folds are likely to facilitate microbial dysbiosis of commensal bacteria and yeast.^26^, ^60^, ^61^, ^62^ Little is known about **bacterial overgrowth syndrome** (BOG) beyond its nonspecific skin lesions such as erythema, hyperpigmentation and lichenification^18^, ^63^, ^64^ and its unusual cytological findings; large numbers of bacteria are typically seen as evidence for overgrowth, but neutrophils and macrophages are lacking or almost absent (Table 5; Figure 4).

**TABLE 5:** Typical presentations and findings of surface pyoderma in dogs.

Presentation	Typical lesions & distribution	Differential diagnoses	Potential inciting causes	Cytological and histopathological findings
Pyotraumatic dermatitis (*acute* moist dermatitis, ‘hot spot’)	Localised area of erythema, exudate, swelling, matted hair Can rapidly progress to erosion, ulceration Cheek, neck, lateral thigh, rump	Pyotraumatic folliculitis and/or furunculosis (deep pyoderma)	Atopic dermatitis, other hypersensitivities, ectoparasites Ear disease, anal sac, disease, arthritis Subcutaneous injection site reactions, adverse topical drug reaction	Predominantly cocci, extracellular and within degenerative neutrophils Superficial neutrophilic and/or eosinophilic perivascular inflammation and dermal oedema, necrosis and erosion to ulceration, crusting^20^, ^67^
Intertrigo (skin‐fold dermatitis)	Erythema, swelling, alopecia, crusting, exudate, erosion, malodour, hyper‐ (rarely de‐)pigmentation Any skin folds, typically facial, lip, vulvar, tail folds	Demodicosis, mucocutaneous pyoderma (superficial pyoderma), DLE/MCLE	Friction from skin rubbing against skin Breed predispositions (e.g. bulldog, Shar Pei, bloodhound, spaniels) and folds developing with obesity Atopy, other hypersensitivities	Predominantly extra‐ and intracellular cocci but often mixed with rods and yeast, inflammatory cells^25^ Epidermal hyperplasia with neutrophilic exocytosis, lymphoplasmacytic infiltrate in superficial dermis (‘lichenoid band’)^68^
Bacterial overgrowth syndrome (BOG)	Erythema, exudate, lichenification, hyperpigmentation Ventral abdomen, interdigital, pinnae	*Malassezia* dermatitis or overgrowth, other chronic inflammatory or hyperplastic skin changes	Atopic dermatitis, other hypersensitivities Keratinisation disorders Endocrinopathies	High numbers of cocci yet no or few neutrophils Perivascular‐interstitial hyperplastic and hyperkeratotic dermatitis with lymphocytic exocytosis, focal or diffuse spongiosis and occasionally epidermal neutrophilic micro‐abscesses, pigmentary incontinence

Abbreviation: DLE/MCLE, discoid lupus erythematosus/mucocutaneous cutaneous lupus erythematosus.

The aetio‐pathogenesis of surface pyodermas is poorly studied yet all are secondary to inflammatory underlying primary conditions, such as atopic skin disease or friction from skin rubbing against adjacent fold skin. The term ‘dysbiosis’ has been proposed for the microbial changes, and overgrowth of *S. pseudintermedius* with a reduction in microbial diversity has been identified in microbiome studies in atopic dogs.^65^ The inclusion of such dysbioses among the pyodermas has become controversial because bacteria are not invading tissue as in the traditional sense of ‘infection’.^66^ For BOG in particular, the term ‘pyoderma’ is a misnomer as neither pus nor inflammatory cells are detectable on the skin.

### Diagnosis

The diagnosis of surface pyoderma is clinical, based on a compatible history and the presence of suggestive skin lesions in typical locations. Cytology is then needed to confirm bacterial involvement and the requirement for antimicrobial treatment. Cytology will also help with tailoring decisions on active ingredients for topical therapy in some cases (see Section ‘Topical antimicrobial therapy’). BC/AST is not needed as only topical treatment should be used. Diagnosis and correction of the underlying primary disease will be key to achieving long‐term treatment success (see Section ‘Diagnostic approach and diagnostic tests’).

Skin lesions of pyotraumatic dermatitis can resemble those of a form of deep pyoderma known as ‘pyotraumatic folliculitis and furunculosis’ (see Section ‘Deep pyoderma’). Hair around the lesion may need to be clipped and the skin carefully examined for the absence of signs suggestive of deep pyoderma. Papules, pustules, haemorrhagic crusts, swelling and furuncles in the periphery are termed ‘satellite’ lesions, and would indicate haematogenous spread and deep pyoderma requiring a different treatment approach (see Section ‘Deep pyoderma’). A study of 44 dogs showed that lesions of pyotraumatic dermatitis can be difficult to differentiate from pyotraumatic folliculitis/furunculosis clinically and even histopathologically.^20^ A similar uncertainty about depth of infection also may be encountered in intertrigo affecting the lip folds where clinical signs may resemble those of mucocutaneous pyoderma (see Section ‘Superficial pyoderma’).

### Treatment recommendations for surface pyoderma

#### Topical antimicrobial therapy

Although commonly seen in practice, few clinical treatment trials have been published for surface pyoderma (Table 6).

**TABLE 6:** Clinical trials on treatment of surface pyoderma in dogs (English language publications, full manuscripts).

	References	Agents tested	Study design	SORT level of evidence	Dogs (n) enrolled/completed	Duration	Outcome
Pyotraumatic dermatitis	Cobb et al., 2005^25^	0.5% fusidic acid and 0.1% betamethasone‐17‐valerate (*n* = 51) versus systemic dexamethasone once & amoxicillin‐clavulanate (*n* = 53)	RCT	1	104	7 days	69% versus 79% achieved ‘good’ or ‘complete’ response. No significant difference detected between topical and systemic
	Holm et al., 2004^20^	All dogs: once or twice daily saline wash, Elizabethan collar, aluminium acetate or boric acid astringent, 3 weeks cefalexin 20 mg/kg twice daily	Prospective case series	2	44	7–10 days	44/44 improved after 7–10 days, 5/44 given additional prednisolone after 7–10 days; some may have been pyotraumatic furunculosis
	Schroeder & Rème, 2011^74^	0.0584% hydrocortisone aceponate (HCA) spray once daily (*n* = 10) versus 0.1% prednisolone, 0.5% neomycin, 0.5% sulfur, 3% zinc oxide lotion twice daily (*n* = 10)	RCT	1	20	7 days	Pruritus and aggregate lesions scores improved in both groups; both scores were lower on Day 2 for HCA; Day 6, lesions resolved in 7/10 dogs with HCA versus 3/10 in neomycin group
	Schroeder et al., 1996^59^	Shave, wash, cream twice daily: (a) neomycin (*n* = 10) (b) 0.1% prednisolone (*n* = 10) (c) neomycin & prednisolone (*n* = 10) (d) methylparaben (*n* = 5) (e) zinc oxide (*n* = 5)	RCT	2	40	7 days	Full recovery in all groups. Quickest with neomycin & prednisolone combination. Lesion surface reduced in all groups. Reduction in inflammation most rapid for group (c)
Intertrigo	Brosseau et al., 2020^75^	Medical honey (*n* = 19) versus placebo (*n* = 16) in nasal intertrigo, once daily after washing and drying	RCT	1	30/35	21 days	Placebo better at reducing clinical lesion scores and cytological scores; honey better at reducing owner‐assessed pruritus
BOG syndrome	Gatellet et al., 2021^76^	Daily ophytrium and 3% chlorhexidine digluconate pad application to affected skin; cutaneous bacterial (*n* = 11) or *Malassezia* (*n* = 7) overgrowth	Prospective case series	2	18	14 days	Reduced bacterial counts, mean global scores and pruritus scores improved, 88.9% achieved >70% microbial decrease
	Pin et al., 2006^63^	Cefalexin 15 mg/kg per os twice daily (topical treatment not allowed)	Prospective case series	3	8	28 days	All improved and bacterial counts reduced on cytology and from culture. 5/8 had allergic skin disease
	Viaud et al., 2012^64^	3% chlorhexidine (*n* = 22) versus 2.5% benzoyl peroxide (*n* = 18) shampoo twice weekly	RCT	1	34/40	6 weeks	Reduction in bacterial and lesion scores in both groups and no difference in time to cytological cure between groups

Abbreviations: BOG, bacterial overgrowth; RCT, randomised, controlled trial.

Topical antimicrobial therapy is the treatment‐of‐choice for surface pyoderma (SOR A).

Topical antiseptics (e.g. chlorhexidine) should be prioritised over topically used antibiotics (e.g. fusidic acid, mupirocin) (see Section ‘Topical antimicrobial therapy’). Antiseptics are expected to have broad activity against most relevant skin pathogens and active ingredients can therefore be chosen empirically. If topical antibiotics are needed, active ingredients should be chosen based on cytology results (see Section ‘Which active ingredient?’). For localised lesions of pyotraumatic dermatitis, gels or sprays may be most suitable (following cleaning of discharge or crust). Wipes or gels will allow good penetration into deeper parts of folds in intertrigo, and frequent application of product, at least once daily and typically twice daily, will be needed. Shampoos or sprays may be most suited to widespread affected areas (see Section ‘Topical antimicrobial therapy’). Analgesia should be considered to facilitate topical therapy, particularly for pyotraumatic dermatitis lesions which are often painful.

Systemic antimicrobial therapy should be avoided in cases of surface pyoderma given the location and direct accessibility of microbial pathogens. The efficacy of topical therapy alone was equivalent to systemic therapy in dogs with pyotraumatic dermatitis^20^, ^25^ Topical treatment alone is effective in BOG and intertrigo (Table 6).

#### Anti‐inflammatory therapy

Combination therapy of topical antimicrobial therapy with topical glucocorticoids or with a short course (5–7 days) of systemic glucocorticoids at anti‐inflammatory doses (SOR A) or antipruritic medication may be helpful in cases of pyotraumatic dermatitis and of intertrigo where an inflammatory or pruritic primary cause is involved.

A major role for inflammation and hence the need for anti‐inflammatory treatment in surface pyodermas is widely accepted.^69^, ^70^ Of the eight clinical studies published for surface pyoderma, three RCTs showed good efficacy of the glucocorticoid products (Table 6; Box 3, 4).

BOX 3 **Example of approach to a dog with pyotraumatic dermatitis (‘hot spot’)**

1

- Approach the lesion carefully; it may be painful. Consider systemic or local pain relief.
- Clip, including the surrounding area
- Clean to remove debris and biofilm
- Apply antiseptic (e.g. 2%–4% chlorhexidine) or topical antibiotic with or without topical glucocorticoid preparation once or twice daily for 7 days.
- Consider 5–7 days of oral prednisolone (0.5–1.0 mg/kg body weight once daily) or dexamethasone (0.1 mg/kg once daily) or of a Janus kinase (JAK) inhibitor to reduce inflammation, pain and itch.
- Investigate and manage underlying primary causes (e.g. review ectoparasite prophylaxis, examine ears and anal sacs and consider underlying allergic disease)

BOX 4 **Example of approach to a dog with intertrigo (skin‐fold dermatitis)**

1

- Clean affected skin to remove debris and biofilm
- Apply antiseptic therapy, for example wipe once or twice daily with 2%–4% chlorhexidine wipes or apply as a solution or spray.
- If rods dominate on cytology, consider adding an agent with activity against Gram‐negative bacteria, for example silver sulfadiazine or gentamicin‐containing creams or drops.
- For facial‐fold dermatitis in proximity to the eyes, formulations with topical antibiotics suitable for ophthalmic use, for example fusidic acid‐ or gentamicin‐containing eye drops, can be considered.
- In cases that present with severe erythema, swelling and pain, consider the use of topical formulations combining antimicrobials (antiseptics or topically used antibiotics) with topical glucocorticoids. Apply twice daily for 7–10 days, then re‐examine.
- If erosions or ulcers are noticed, the use of hypochlorous acid products (safe in open wounds, eyes and mucous membranes) may be preferred until the defects have healed.^71^, ^72^
- Depending on the case, consider weight loss, dental treatment and management of urinary disease.
- In some dogs, surgical management of folds (cheiloplasty, vulvoplasty) or caudectomy for ingrown tails may be needed.

Clinical resolution of surface pyoderma can be assumed when pre‐treatment lesions have significantly improved or resolved and cytological results from previously affected areas are normal. This can be expected within 7–14 days. Re‐assessment of clinical signs by a veterinarian and repeat cytology are recommended if progress is deemed poor by the owner. Compliance and the diagnosis of underlying primary causes may need to be reviewed and corrected.

#### Long‐term management

Antiseptic treatment can be continued proactively on previously affected skin, potentially life‐long, where the primary underlying causes cannot be resolved (e.g. skin folds) and the risk of recurrence remains (SOR C).

Data on long‐term management of surface pyodermas are not published, but recurrences are well‐recognised when primary causes remain elusive or cannot easily be corrected. Weight loss or surgical management of skin folds may be helpful in some cases of intertrigo. Frequencies of proactive antiseptic applications will vary and need to be identified by tapering until recurrence is seen on a case‐by‐case basis. Although there have been intermittent reports of in vitro chlorhexidine tolerance in staphylococci, the concentrations of chlorhexidine achieved through appropriate clinical use far exceed the maximum minimum inhibitory concentrations (MICs) described in the literature, despite decades of use of this active ingredient. No true antimicrobial resistance resulting in clinical treatment failure of staphylococcal infection using chlorhexidine has yet been described.

If diagnostic assessment for underlying causes has raised suspicion for primary allergic causes, proactive therapy with topical anti‐inflammatory therapy may be considered to prevent relapses.^73^ Application of astringent powders, herbal products to heal pyotraumatic dermatitis or barrier creams to reduce friction and maceration in skin folds, and thereby provide a skin environment less prone to microbial dysbiosis, have been mentioned historically yet evidence for efficacy is not available.

## 6 SUPERFICIAL PYODERMA

### Clinical presentations

Superficial pyoderma is defined as a bacterial infection affecting the epidermis and the hair follicle. Four clinical presentations are described (Table 7).

**TABLE 7:** Typical presentations and characteristics of superficial pyoderma in dogs.

Presentation	Typical lesions	Distribution	Differential diagnoses	Cytological & histopathological findings	Comments
Superficial bacterial folliculitis (SBF)	Follicular papules, pustules, epidermal collarettes, multifocal areas of alopecia In short‐coated breeds, patchy alopecia (‘moth‐eaten’ coat) may predominate	Ventral abdomen, medial thighs, dorsal neck Can be generalised	Other follicular diseases: demodicosis, dermatophytosis, sebaceous adenitis, sterile pustular dermatitis, leishmaniosis Follicular papules may mimic urticaria in short‐coated breeds	Intra‐ or extracellular bacterial cocci and neutrophils Luminal folliculitis and subcorneal pustules within follicular openings containing Gram‐positive bacterial cocci	Most common type of superficial pyoderma in dogs. Often pruritic
Exfoliative superficial pyoderma (previously ‘superficial spreading’)	Large expansile epidermal collarettes (in the absence of papules, pustules), central alopecia and hyperpigmentation, erythematous rim	Ventrum, can be generalised	Dermatophytosis, erythema multiforme, drug eruptions (cutaneous adverse drug reactions), VCLE	Intra‐ or extracellular bacterial cocci and neutrophils Intracorneal clefts, which may contain basophilic cellular debris and bacterial cocci	Shetland sheepdogs and collies predisposed
Impetigo and bullous impetigo	Nonfollicular pustules, often fragile and with erythematous rim, yellow or green content, epidermal collarettes.	Groin, axillae. Can be generalised	Pemphigus foliaceus (also associated with large, fragile pustules and crusts)	Intra‐ or extracellular bacterial cocci and neutrophils Neutrophilic subcorneal pustules, often spanning multiple follicular units, and containing Gram‐positive bacterial cocci	Puppies and juvenile dogs. As ‘bullous impetigo’ in immunocompromised adults
Mucocutaneous pyoderma	Erythema, swelling, crusts, exudate, erosions, ulcers, pigmentary changes, peripheral alopecia	Mucocutaneous junctions, lips, perioral skin and also eyelids, around nose, perianal and peri‐genital	Intertrigo (lip‐/facial‐fold dermatitis), discoid or mucocutaneous lupus erythematosus (DLE/MCLE)	Neutrophils, nuclear streaming, and bacterial cocci (intra‐ or extracellular) Histological changes overlap with (DLE/MCLE) Superficial dermal infiltrates of lymphocytes, plasma cells, lichenoid band and basal cell apoptosis	German shepherd dogs predisposed

Abbreviations: DLE/MCLE, discoid lupus erythematosus/mucocutaneous cutaneous lupus erythematosus; VCLE, vesicular cutaneous lupus erythematosus.

**Superficial bacterial folliculitis** is by far the most common type of superficial pyoderma in the dog, with *S. pseudintermedius* isolated most often in pure culture.^5^ The dog's predisposition to folliculitis is thought to be facilitated by a missing sebum plug around the follicle opening; it is likely that there are other anatomical and physiological characteristics, too.^77^, ^78^

**FIGURE 5:**
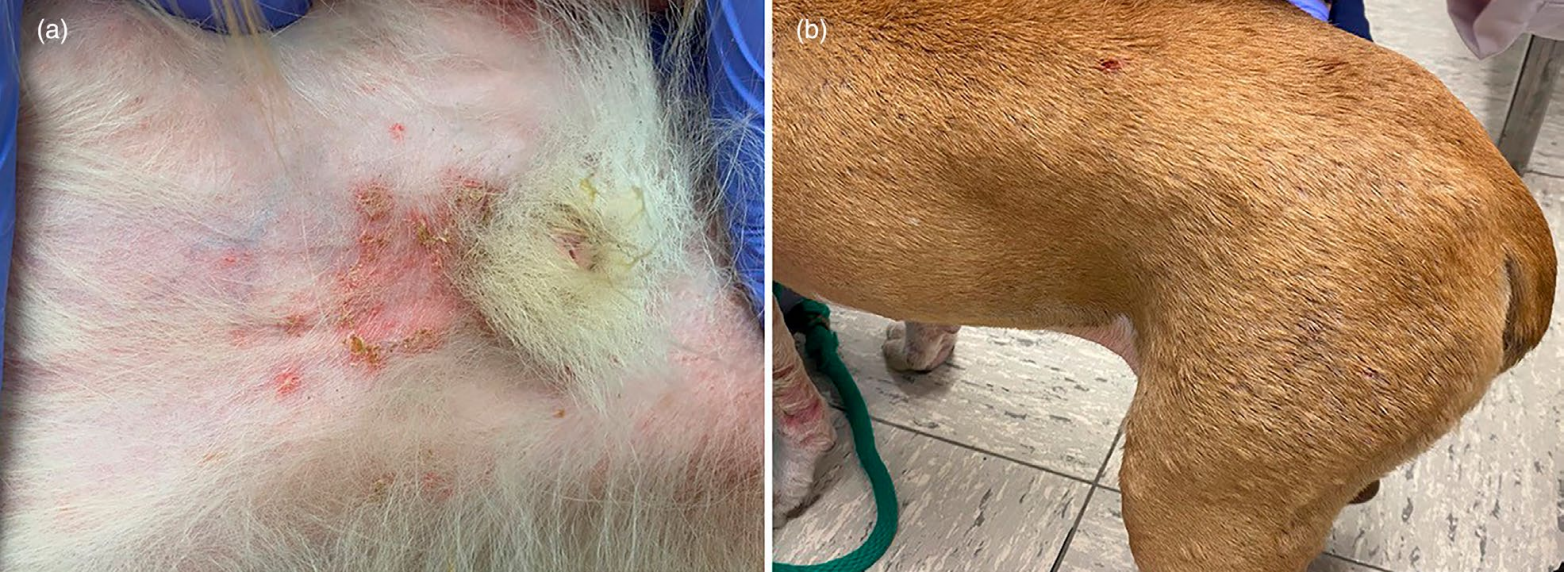
Examples of superficial pyoderma in dogs. (a) Superficial bacterial folliculitis affected the inguinal region of a Golden retriever. (b) On the trunk and lateral thigh of an American pitbull terrier.

Hallmark lesions of SBF are often found concurrently on the same dog, starting with erythematous follicular papules, progressing to pustules which will rupture to form epidermal collarettes with an erythematous peripheral ring and a healing, sometimes hyperpigmented centre; eventually leaving focal areas of alopecia. Any area of haired skin can be affected, yet predilection sites in medium‐ to long‐coated breeds are the ventral abdomen and medial thighs, while the distribution is often truncal in short‐haired breeds with focal areas of alopecia being most prominent, giving the dogs a ‘moth‐eaten’ appearance.^5^ In some cases of SBF, mainly in short‐coated dogs, follicular papules, pustules and crusts also may cause elevation of tufts of hairs, which may be mistaken for urticaria and are persistent (Figure 5). Occasionally, scaling, loss of coat lustre, increased shedding or malodour may be the presenting signs. In some breeds, such as the American cocker spaniel, crusts may be notably hyperkeratotic and plaque‐like. Postinflammatory hyperpigmentation is common and may persist following lesion resolution.^5^, ^18^, ^52^

**FIGURE 6:**
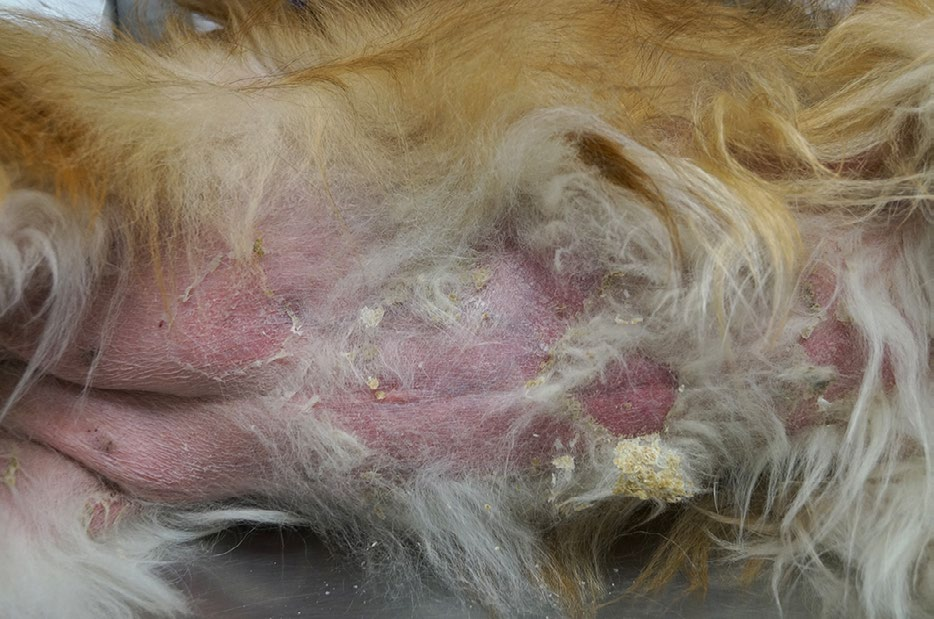
Exfoliative superficial pyoderma in a Shetland sheepdog.

**Exfoliative superficial pyoderma** (previously termed ‘superficial spreading pyoderma’) presents with strikingly large, expansile epidermal collarettes, resembling large target lesions (Figure 6). This phenotype of superficial pyoderma is particularly recognised in Shetland sheepdogs, Australian and German shepherd dogs, and collie breeds, although other breeds and mixed breeds can be affected.^18^, ^19^, ^54^, ^79^, ^80^ The histopathological findings of exfoliative superficial pyoderma are distinct from those for SBF owing to the presence of frequent intracorneal clefts which may contain basophilic cellular debris and bacterial cocci.^54^, ^81^ A less common form of exfoliative superficial pyoderma has been described in dogs, characterised by generalised erythema with large sheets of scale.^19^, ^54^, ^80^

**FIGURE 7:**
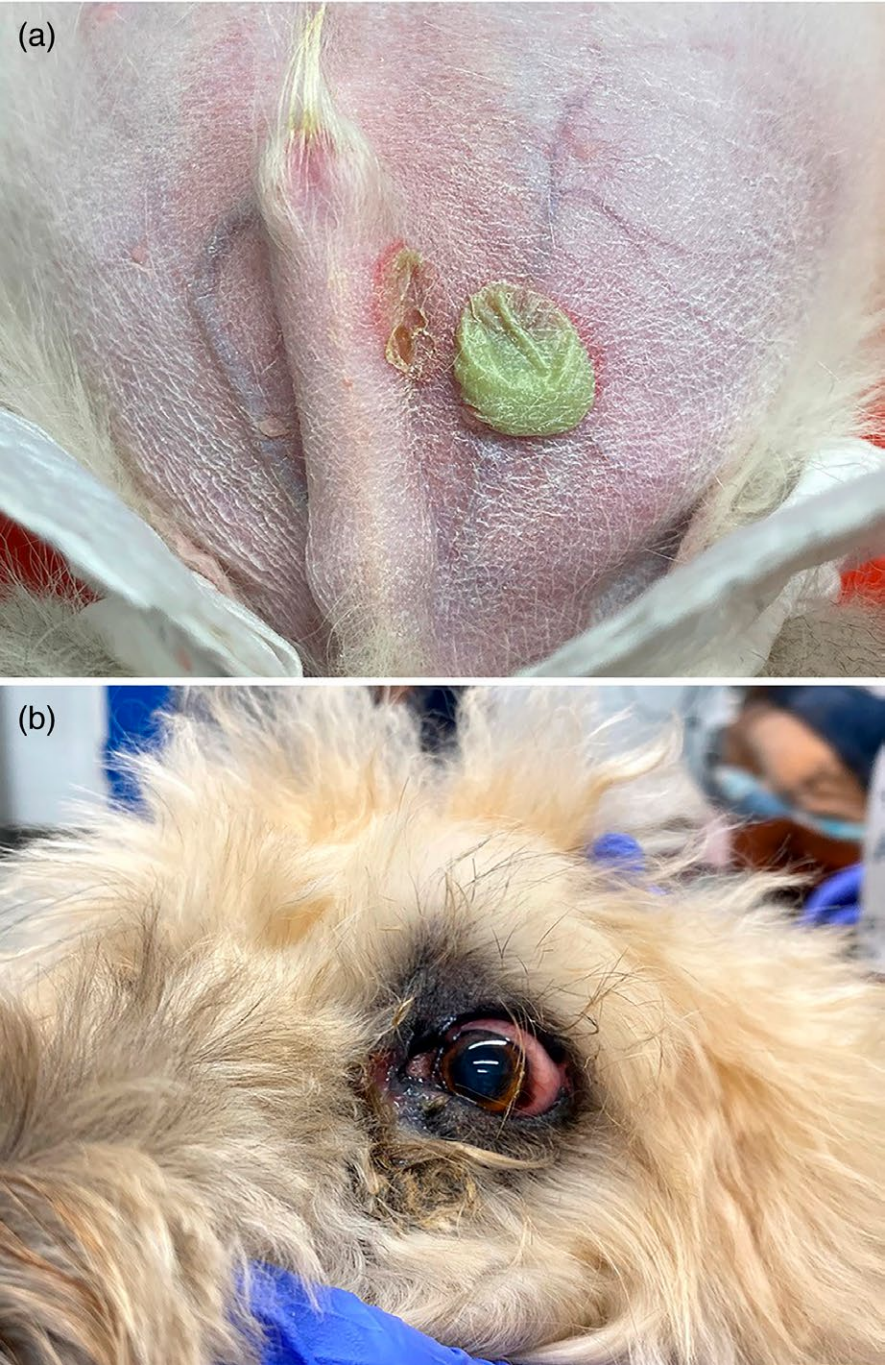
(a) Impetigo on the ventral abdomen of a Siberian husky puppy. (b) Mucocutaneous pyoderma of the eyelid margin and medial canthus in a soft‐coated Wheaten terrier.

**Impetigo** in juvenile dogs is often visually distinctive from SBF owing to the large size of the pustules, their nonfollicular orientation, and a lack of concurrent erythematous papules (Figure 7). Risk factors that have been suggested in young dogs include poor nutrition, endoparasitism, or concurrent viral infection.^18^, ^19^, ^80^, ^81^, ^82^ Bullous impetigo, with similarly large, nonfollicular pustules and also with bullae in glabrous areas, is recognised in immunocompromised adult dogs, particularly in association with iatrogenic or spontaneous hyperadrenocorticism.^2^ It is different from impetigo in humans, which is a highly contagious skin infection caused by exfoliative toxin‐producing staphylococci or group A streptococci.

**Mucocutaneous pyoderma** is a poorly characterised entity, recognised by erythema, erosions, ulcers, crusts and pigmentary changes in mucocutaneous locations that resolve with antimicrobial treatment and extend beyond the epidermis (Figure 7). Microbes involved are not well documented yet are likely to represent skin microflora and *Pseudomonas* spp., particularly in moist areas. When affecting the perioral or perivulvar areas, mucocutaneous pyoderma can be difficult to differentiate from intertrigo (see Section ‘Surface pyoderma’). Further characterisation of these two presentations is required to confirm whether they are distinct presentations or whether mucocutaneous pyoderma is a continuum of a dysbiosis at anatomically and immunologically distinct sites. For the purpose of these guidelines, mucocutaneous pyoderma is included as superficial pyoderma when lesions are similar to those found with intertrigo yet are more severe, occasionally extending to ulceration as a sign of deeper infection. Any breed can be affected, but German shepherd dogs are reported as predisposed (in contrast to intertrigo for which spaniels or any breed with prominent folds are reported as predisposed).^19^, ^83^ Mucocutaneous pyoderma will also need to be differentiated from mucocutaneous lupus erythematosus (MCLE). If skin lesions remain after infection or dysbiosis have been treated successfully, histopathological evaluation from biopsy specimens will be indicated.^84^

### Diagnosis

Diagnosis of **SBF** is based on clinical and cytological (cocci and neutrophils; see Section ‘Diagnostic approach and diagnostic tests’) findings and the exclusion of other follicular diseases.

It is important to exclude demodicosis (e.g. with hair plucks or deep skin scrapings) and dermatophytosis (Wood's lamp examination; direct examination of hairs for fungal hyphae or arthrospores; fungal culture and/or dermatophyte PCR).^44^ Pemphigus foliaceus can mimic SBF, and the suspicion for this condition should be increased if lesions are seen in areas not typical for SBF (e.g. pinnal surfaces, dorsal muzzle, paw pads) or if cytology reveals numerous acantholytic keratinocytes, in addition to neutrophils and eosinophils, and few‐to‐no bacteria.^85^ Biopsies for histopathology should be considered to identify immune‐mediated, sterile pustular diseases or sometimes leishmaniosis if lesions remain after the pyoderma has been treated adequately and cytology no longer shows evidence of bacterial infection.

The large, expansile epidermal collarettes of **exfoliative superficial pyoderma** are visually distinctive yet should always be confirmed cytologically, following exclusion of demodicosis and dermatophytosis. Histopathology is not typically necessary for diagnosis.

**Impetigo** is diagnosed based on signalment (young dog), characteristic interfollicular pustules, and a cytology that supports bacterial infection. Histopathological evaluation may be needed in rare cases to differentiate impetigo from pemphigus foliaceus. In impetigo, acantholysis is typically mild or absent, though it may be more prominent, possibly resulting from the expression of staphylococcal exotoxins capable of degrading desmosomal adhesion molecules.^80^, ^81^, ^86^, ^87^

The diagnosis of **mucocutaneous pyoderma** is clinical and based on recognising lesions at mucocutaneous junctions combined with cytology findings supporting infection (bacteria and neutrophils). Confirmation of the diagnosis is by observation of lesion resolution on antimicrobial treatment.^21^, ^84^, ^88^

### Treatment recommendations for superficial bacterial folliculitis (SBF), impetigo and exfoliative superficial pyoderma

#### Topical antimicrobial therapy

Topical antimicrobial therapy as the sole antibacterial treatment is the treatment‐of‐choice for canine superficial bacterial folliculitis (SOR A) and for other presentations of superficial pyoderma.

For **SBF**, topical antimicrobial therapy as the sole antibacterial treatment has been found effective in 12 of the 13 currently published clinical trials, including eight randomised controlled trials (Table 8, Box 5). Clinical efficacy was seen for 2–4% chlorhexidine products, a 2.5% benzoyl peroxide shampoo, a sodium hypochlorite/salicylic acid shampoo, carbonated water and for two polyherbal preparations and not for stannous fluoride. Product formulations containing these active ingredients are widely available (see Section ‘Topical antimicrobial therapy’). Topical antimicrobial therapy alone also was found to be equally effective as systemic antimicrobial therapy in an RCT comparing a combination of 4% chlorhexidine digluconate shampoo and solution versus amoxicillin‐clavulanate after 4 weeks of therapy.^89^

**TABLE 8:** Published clinical trials on topical antimicrobial therapy used as the sole antimicrobial treatment for canine superficial pyoderma, identified using the search criteria of Barker et al. 2022.^11^

References	Active ingredient and dogs (*n*) completed	Study design	LoE	Duration	Outcome
Aiemsaard et al., 2022^90^	Clove essential oil with 10% ethyl alcohol (*n* = 4) versus 2% chlorhexidine spray twice daily (*n* = 4)	Prospective case series	3	15 days	Improvement in staphylococcal counts and lesion scores in both groups; negative culture in all dogs by Day 10
Borio et al., 2015^89^	4% chlorhexidine shampoo twice weekly and daily spray (*n* = 31) versus amoxicillin‐clavulanate 25 mg/kg per os twice daily (*n* = 20)	RCT	1	28 days	All lesions resolved in both groups (8 dogs with MRSP)
Bryan et al., 2012^91^	Various (incompletely described)	Retrospective case series	3	Unknown	MSSP all resolved; MRSP (*n* = 24): 15 resolved, 4 improved, 5 did not improve
Duangkaew et al., 2017^92^	Polyherbal shampoo and spray (*n* = 16)	Prospective case series	3	56 days	15 dogs improved by 75%–100%, no improvement in one dog
Fadok and Irwin 2019^93^	Sodium hypochlorite & salicylic acid baths three times weekly (*n* = 17)	Prospective case series	2	28 days	Clinical severity scores decreased progressively and significantly
Hsiao et al., 2021^94^	1.5% olanexidine spray once daily (*n* = 14) versus 3% chlorhexidine shampoo once weekly (*n* = 14)	RCT	1	10 days	No significant difference between groups identified, clinical scores halved
Iyori et al., 2022^95^	Carbonated water (*n* = 10) versus noncarbonated water (placebo) (*n* = 9), both once weekly	RCT	2	21 days	Clinical scores significantly reduced in treated group
Loeffler et al., 2011^96^	3% chlorhexidine (*n* = 10) versus 2.5% benzoyl peroxide shampoos, twice weekly (*n* = 10)	RCT	1	21 days	70% major improvement in both groups. Resolution in 7/10 chlorhexidine versus 2/10 benzoyl peroxide
Murayama et al., 2010a^97^	(a) Chlorhexidine 2% or 4%, four washes on half of the body (*n* = 10) (b) Chlorhexidine 2% twice weekly versus cefalexin or minocycline	(a) RCT (b) prospective case series	(a) 1 (b) 3	(a) 7 days (b) 7–21 days	(a) No difference between the two chlorhexidine concentrations. Clinical resolution in all topically treated dogs within 14 days (*n* = 1) or within 21 days (b) 6 improved, 1 partially improved, 1 no change
Murayama et al., 2010b^98^	2% chlorhexidine versus 2% chlorhexidine/2% miconazole twice weekly on half the body (*n* = 10)	RCT	2†	7 days	Clinical signs improved in all dogs, 1 dog with clinical resolution Downgraded for lack of clinical and statistical detail^a^
Murayama et al., 2011^99^	Chlorhexidine 2% at three different doses/area (*n* = 9 in each group)	RCT	2	7 days	Majority of dogs had good response in all three groups
Seltzer et al., 2010^100^	Stannous fluoride 0.2% (with glycerin & zinc gluconate) (*n* = 12) versus vehicle only (*n* = 14), both once daily	RCT	2†	28 days	Investigator scores no different between Days 1 and 28 in both groups Downgraded for inconsistent detail for analysis^a^
Tochio et al., 2023^101^	Erythritol (5%)/0.1% l‐ascorbyl‐2‐phosphate sprayed three times daily onto affected areas	Prospective case series	2	28 days	Clinical scores reduced in all dogs

Abbreviations: LoE, SORT level of evidence; RCT, randomised controlled trial.

^a^ Study LoE amended for reasons given under ‘outcome’; MSSP, meticillin‐susceptible *Staphylococcus pseudintermedius*; MRSP, meticillin‐resistant *S. pseudintermedius*.

BOX 5 **Example approach to a dog with superficial bacterial folliculitis—topical antimicrobial therapy alone (treatment‐of‐choice)**

1

- For widespread lesions, 2%–4% chlorhexidine shampoo at least two to three times per week, 10–15 min contact time, then rinse.
- For localised lesions, 2%–4% chlorhexidine wipes, spray or foam once daily.
- Consider clipping affected areas in long‐coated or plush‐coated dogs.
- Ensure adequate ectoparasite prophylaxis is in place (avoid spot‐on preparations when using frequent shampoo therapy as efficacy may be reduced)
- Re‐assess after 2–3 weeks
- If progress is satisfactory, continue antiseptics until all lesions are resolved and until underlying primary causes have been identified and corrected.
- If progress is poor, review compliance, products and primary underlying disease, then consider systemic therapy.

For **impetigo** and **exfoliative superficial pyoderma**, very little information on treatment has been published. A single open noncontrolled trial showed good efficacy of 3% chlorhexidine shampoo when used twice weekly in 17 puppies with impetigo for 3 weeks.^102^ It is noted that some cases of impetigo resolve spontaneously, particularly when factors such as poor nutrition, poor sanitation and endoparasitism are corrected.^82^ Bullous impetigo in immunocompromised adult dogs is unlikely to resolve spontaneously, and it is critical that the underlying cause be found.^2^ Systemic antimicrobial therapy may need to be considered if lesions do not respond to topical therapy alone.

Response to topical therapy should be re‐assessed by a veterinarian after 2–3 weeks.

Clinical trials have demonstrated lesion resolution in most dogs after 3–4 weeks of therapy, with lesion improvement occurring within 1–2 weeks of starting therapy.^89^, ^93^, ^94^, ^96^, ^97^, ^98^, ^99^, ^103^ Topical antiseptics are then maintained until all lesions are resolved *and* underlying primary causes are identified and addressed.

#### When is systemic antimicrobial therapy indicated for SBF?

We found no clinical reasons or indications in published studies that would justify the selection of systemic over topical antimicrobial therapy alone for superficial pyoderma. However, if the dog's temperament, owner's ability or compliance or available facilities make topical treatment impossible, or if lesions have not improved sufficiently after 2 weeks of topical therapy alone, systemic antimicrobials will need to be considered. Nevertheless, before starting systemic antimicrobials, a re‐evaluation for persistent concurrent disease(s) that may impair resolution should be made.

Systemic antimicrobial therapy should be reserved for cases that have failed to respond to topical antimicrobial therapy alone or if topical therapy is not feasible due to client or patient limitations.

BC/AST and cytology should be used whenever possible to guide systemic drug choices.

Drugs should only be chosen empirically when the risk for meticillin resistance is deemed low, and only first‐choice agents (clindamycin, cefalexin, cefadroxil, amoxicillin‐clavulanate) should be considered for empirical selection.

Based on in vitro and in vivo studies, first‐choice agents should be effective against the majority of meticillin‐susceptible *S. pseudintermedius* (MSSP). However, minor variations in susceptibilities occur even in MSSP so that BC/AST is always justified to guide selection of the most appropriate drug for a particular dog.^8^, ^104^, ^105^, ^106^ BC/AST will be particularly helpful and is likely to be cost‐effective in areas with a high prevalence of MRS, as expenses resulting from ineffective treatment can be avoided. For recommendations on dosages see Section ‘Systemic antimicrobial therapy’ and Tables 14, 15, 16.

#### How long is it to treat superficial pyoderma when using systemic antimicrobial therapy?

An initial 2‐week course may be dispensed, and an appointment for re‐examination by a veterinarian should be scheduled before the end of the course to determine whether systemic treatment can be stopped or whether longer treatment is required (SOR C).

Published clinical trials available for assessing minimum treatment duration for SBF utilised varying outcome measures, methods of lesion assessment, re‐evaluation times and definitions of clinical resolution.^8^, ^107^ While some studies included dogs with SBF as well as dogs with deep pyoderma, others assessed treatment over a 2‐ to 4‐week period yet did not always include a 2‐week assessment (Tables S2 and S4).^108^, ^109^, ^110^, ^111^, ^112^, ^113^, ^114^, ^115^, ^116^, ^117^, ^118^, ^119^ Available data indicate that while not all cases of superficial pyoderma resolve within 2 weeks of systemic antimicrobials, a substantial proportion of dogs will show full clinical response at 2 weeks. Also in support of shorter treatment durations is the result from a field trial used for drug approval and now available through the Freedom of Information Summaries (FOI) from the US Food & Drug Administration (FDA) (www.FDA.gov). In an FDA‐approved trial, 94.8% (118 cases) and 91.2% (117 cases) of dogs receiving cefovecin and cefadroxil, respectively, for the treatment of SBF were considered cured, based on examination for clinical signs of infection, 14 days after beginning treatment.^120^ Our recommendation of shorter antimicrobial courses is further supported by a similar trend in human medicine where there is already good evidence for successful treatment outcomes with shorter treatment durations in the management of skin and soft tissue infections; similar veterinary studies are needed.^121^, ^122^

Where a re‐examination before the end of the initial 2‐week course is not feasible, treatment can be extended to avoid stopping treatment before veterinary assessment. The required treatment duration for systemic therapy in SBF will vary depending on contributing clinical factors and primary underlying disease. However, in view of the urgent need to reduce unnecessary antimicrobial use and following a review of the published literature specifically on treatment duration for SBF, the authors consider that the historically advocated 3–4 weeks duration can no longer be justified and should be replaced by an initial 2‐week course combined with re‐examination while still receiving systemic therapy to determine whether treatment extensions are indicated.^108^, ^109^, ^110^, ^111^ Clients should be carefully instructed to monitor for lesion improvement at home during the treatment period. Improvement of SBF lesions with systemic therapy should start within 5–7 days of instituting appropriate antimicrobial therapy; if lesions are not improving within this time frame, the dog should be reassessed earlier than planned by a veterinarian so that BC/AST and cytology can be implemented to re‐assess therapy. More complicated or chronic cases may require longer treatments, as, reportedly, might cases involving MRS.^91^

A desire for ‘just in case’ longer prescribing to maximise good outcomes may be understandable but can be counterbalanced and replaced by more frequent monitoring of progress in order to minimise unnecessary antimicrobial use. Scheduled and more frequent re‐examinations will provide opportunities for further diagnostics of underlying primary conditions, and for early detection of drug resistance if progress is poor. It is important to recognise that inflammation can persist beyond resolution of infection; if erythema remains, overlong antimicrobial therapy can be avoided if cytological findings no longer show evidence of bacterial infection.

Adjunctive topical antimicrobial therapy is recommended whenever possible (SOR C).

Adjunctive topical antimicrobial therapy may shorten the duration of systemic treatment required. In one open controlled trial with 20 dogs receiving cefalexin for 14 days, severity indices were lower for the 10 dogs that received adjunctive ethyl lactate bathing, as compared to those treated only systemically.^123^ In another study using cefalexin for superficial pyoderma, a notable reduction of clinical scores was seen after 21 days in dogs that received a polyherbal topical preparation concurrently compared to after 28 days in the control dogs.^103^ Fluorescent light therapy used alone has been shown to achieve shorter durations to resolution in a group of 12 dogs compared to eight receiving systemic cefadroxil for their superficial bacterial folliculitis.^124^

Clinical resolution of superficial pyoderma can be assumed, and systemic antimicrobials stopped when primary lesions of pyoderma (papules, pustules and erythematous epidermal collarettes) are no longer found.

The best way to determine the cure of superficial pyoderma (and to decide when to stop systemic antimicrobial therapy) is currently unknown, partly because ‘sterility’ of the skin cannot be used as a measure for treatment success. Following successful treatment, cytology from healed skin will show mainly squames (keratinocytes) while culture can still yield normal bacterial flora, including staphylococci. In cases without primary lesions, where, for example easily epilating hairs may be the only visible clinical signs, the evaluation of clinical resolution may be more difficult but can be supported by cytology. Furthermore, remaining clinical signs may be caused by underlying primary causes (e.g. erythema owing to allergic disease) or they may be secondary lesions related to pyoderma and slow to resolve (e.g. patchy alopecia remaining for ≤6 weeks after papules, pustules and epidermal collarettes have healed).

Topical antiseptic treatment can be continued longer than systemic therapy, and is potentially life‐long, where the primary causes cannot be resolved and the risk of recurrence remains.

There is no evidence to support extending systemic antimicrobial therapy beyond the resolution of clinical signs associated with infection; instead, underlying primary causes must be identified and addressed.

The frustrating tendency of canine SBF to frequently recur has previously led to the recommendation of extending treatment for 1 week beyond clinical cure with the intention to reduce the risk of recurrence.^107^ In the absence of supportive data, such prophylactic systemic therapy cannot be recommended and should be replaced with proactive topical antimicrobial therapy and intensified diagnostic searches for underlying causes (Section ‘Preventing recurrences of pyoderma’).

### Treatment recommendations for mucocutaneous pyoderma

Topical antimicrobial therapy alone is the treatment‐of‐choice for most cases of canine mucocutaneous pyoderma. Topical therapy in the form of gel, ointment or cream preparations is recommended (see Section ‘Product formulations’). For cases that present with extensive ulceration and pain, and for those that have failed to respond to topical antimicrobial therapy alone, systemic antimicrobials may be considered.^83^ Where systemic antimicrobial therapy is considered, drug selection should always be based on BC/AST results as the involved pathogens may be diverse and show variable susceptibilities (Box 6).

BOX 6 **Example approach to a dog with mucocutaneous pyoderma**

1

- Treat the bacterial infection topically, guided by cytological findings.
- Prioritise topical antimicrobial therapy with gel, ointment or cream preparations over more liquid solutions to avoid mechanical removal.
- If erosions or ulcers are noticed, the use of hypochlorous acid products should be considered owing to their safety on open wounds.
- Re‐assess after 2–3 weeks
- If lesions (e.g. erosions, ulcers, crusts) remain
  - and cytological evidence of infection persists, consider adding systemic antimicrobial therapy
  - following cytological clearance of infection, consider taking biopsies for histopathological evaluation to investigate other differentials such as leishmaniosis or discoid or mucocutaneous lupus erythematosus.
- Investigate, correct or manage underlying primary causes
- If lesions present as chronically relapsing, consider proactive therapy, for example daily 2–4% chlorhexidine wipes.

## 7 DEEP PYODERMA

### Clinical presentations

**FIGURE 8:**
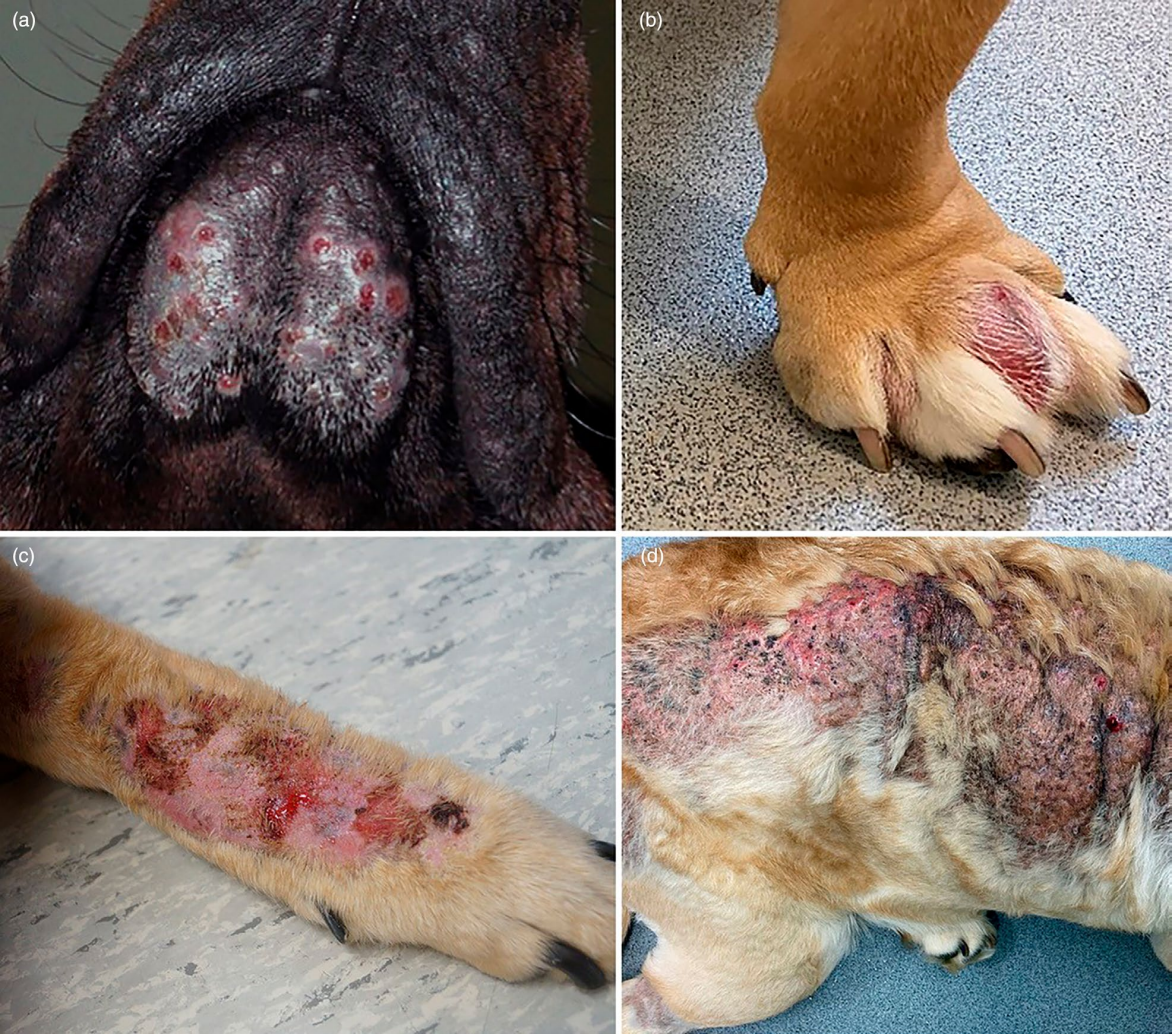
Examples of deep pyoderma in dogs. (a) Chin pyoderma (‘chin acne’). (b) Interdigital infected nodule. (c) Acral lick dermatitis. (d) Widespread deep folliculitis and furunculosis.

Deep pyoderma is a bacterial infection occurring in the dermis and sometimes extending into the subcutaneous tissue (panniculus). It includes a heterogeneous group of different clinical presentations which can be widespread or localised (Table 9, Figure 8). In localised presentations, the terminology for the disease typically relates to the site of the primary clinical entity.

**TABLE 9:** Clinical presentations of deep pyoderma and typical associated clinical findings.

Presentation		Typical skin lesions	Distribution	Differential diagnoses	Comments
Widespread/generalised	Deep folliculitis and furunculosis (cellulitis)	Papules, pustules, haemorrhagic crusts, tissue swelling, erythema, alopecia, draining sinuses, ulceration, matted hair in long‐coated dogs If severe: friable darkly discoloured skin, may slough	Trunk, limbs	Calcinosis cutis (especially if affecting dorsal neck and thorax in short‐haired breeds), demodicosis, dermatophytosis, eosinophilic furunculosis of the face, juvenile cellulitis, sterile immune‐mediated skin diseases	Extended diagnostic testing for underlying causes is indicated depending on clinical suspicion (see Section ‘Investigations into underlying primary causes’)
	Postgrooming furunculosis	Erythematous swollen areas, pustules, haemorrhagic crusts or bullae, erosions, ulcers, draining nodules	Dorsal neck and trunk	Typically distinct presentation when considering the history Immune‐mediated or neoplastic disease (epitheliotropic lymphoma)—usually not acute Back pain in acute phase	Skin lesions develop within 24–48 h after bathing or water immersion combined with trauma (grooming, clipping); fever, lethargy. Often involves *Pseudomonas* spp
Localised	Pyotraumatic folliculitis and furunculosis	Erosion or ulceration, thickening, plaque‐like lesion, often surrounded by satellite papules and pustules, erythema, alopecia	Cheek and neck region, lateral thighs, trauma points	Pyotraumatic dermatitis (‘hot spot’) which is typically acute in onset (see Section ‘Surface pyoderma’) All differential diagnoses listed above for generalised furunculosis	Labrador retrievers and other large breeds predisposed Often with an underlying primary pruritic condition
	Acral lick dermatitis/ furunculosis	Alopecic plaque, hyperpigmented, eroded or centrally ulcerated, sometimes with draining sinuses	Dorsal aspect of the lower forelimb(s), occasionally hind limb(s)	If single lesion: neoplasia, injury, foreign body, deep fungal infection, leishmaniosis	Consider primary triggers: foreign body (single lesion), allergy, endocrinopathy, joint or bone disease (if all ruled out, a psychogenic disorder should be considered)
	Infected interdigital nodules (pedal furunculosis, interdigital‘cysts’)	Erythematous nodule (furuncles), draining tracts (fistulae) ± serosanguinous discharge. Palmar/plantar surfaces may show comedones, ‘pseudopad’ formation	Typically at multiple interdigital spaces, often seen dorsally first and usually originating from palmar/plantar surface	If single lesion: foreign body (grass seed, thorns, splinter), deep fungal infection, neoplasia, injury, arteriovenous fistulae	Primary causes include foreign bodies, demodicosis, allergies, endocrinopathy, gait abnormality, excess body weight, weight bearing on haired skin leading to hair shaft foreign body reaction, cyst formation
	Pressure point/callus pyoderma	Erythema, draining sinus or fistula, swelling, haemorrhagic or yellow crust (complicating the callus‐associated lesions of hyperkeratosis, lichenification, hyperpigmentation)	Elbows, hocks, sternum	Noninfected callus, neoplasia, deep fungal infection, other deep bacterial (*Nocardia*, *Actinomyces*) infection	Large breeds, heavy weight, sleeping on hard surface; sternum of short‐coated, deep‐chested dogs
	Chin pyoderma (‘chin acne’)	Papules, ± ulceration, erythema, haemopurulent exudate (bleeding if vibrissae are affected)	Chin, muzzle, lips, ventral neck	Demodicosis	Likely primary causes aggravated by, for example trauma (rubbing). Mostly young dogs and short‐haired breeds

The proximity of bacterial infection to dermal blood vessels makes deep pyoderma a potentially serious and debilitating disease with a risk of haematogenous spread and progression to septicaemia.^125^ Infected areas that involve marked, diffuse inflammation (cellulitis) are frequently painful so that signs of self‐trauma may be absent. In presentations where irritation or discomfort is part of the underlying primary problem, self‐trauma can be intense, for example in acral lick dermatitis (ALD). If deep infection is severe, lesions may heal with scarring.

Skin lesions indicative of deep pyoderma include haemorrhagic crusts and haemopurulent discharge, denoting an extension of infection into the dermal skin layer where blood vessels reside. Also suggestive of deep pyoderma are furuncles, which are defined as infected, ruptured hair follicles that appear as large (>3 mm diameter) red‐to‐bluish papules from which pus can be expressed. Although the term ‘furuncle’ was formative in the naming of important diseases, its use (similar to that of ‘carbuncle’ for infection that spans several adjacent hair follicles) has become blurred in the recent veterinary literature. Nodules, ill‐defined swellings, fistulae and sinuses also can be associated with deep pyoderma. Ulcers may occur with infectious and noninfectious processes, yet their presence in a dog with bacterial infection indicates involvement of deeper skin layers and thus deep pyoderma (Table 9). Necrotic changes may follow in severe advanced cases and should prompt urgency. The umbrella terms ‘skin and soft tissue infections’ or ‘cellulitis’ are commonly used in human medicine when referring to deep bacterial skin infections, but differentiation of the specific presentation in dogs has merit as management varies, particularly that of underlying primary causes.

The pathogenesis of deep infections is largely presumed. An extension of bacterial folliculitis into furunculosis via rupture of infected hair follicles has been proposed as a likely pathogenesis for widespread deep pyoderma, while some localised lesions may occur following traumatic processes (e.g. cuts, bites and penetrating foreign bodies, including hair shaft fragments) that introduce bacteria into deeper layers. The course of the disease then largely depends on the host's ability to mount an appropriate immune response, and contain and resolve infection. Infection can spread along tissue planes and progress gradually or explosively. The latter is more typical for infections involving pathogens such as β‐haemolytic *Streptococcus canis*, for example in necrotising fasciitis.^126^ Histopathologically, the inflammatory infiltrate in deep pyoderma is predominantly neutrophilic or pyogranulomatous, and focussed around hair follicles and adnexa.

Deep bacterial skin infections that are not included in these guidelines are anaerobic cellulitis, infections caused by specific agents causing a nodular or diffuse (pyo)granulomatous dermatitis and panniculitis (e.g. botryomycosis, mycobacterial granulomas) and subcutaneous abscesses.^127^ Information on approaches to these rarer diseases can be found elsewhere.^2^, ^3^

**German shepherd dog deep pyoderma (GSP)** as a distinct entity was purposefully omitted from the list of deep pyoderma presentations in these guidelines. GSP was previously recognised as a very severe and widespread deep pyoderma almost exclusively affecting some middle‐aged German shepherd dogs (GSDs) and their crosses.^128^ The condition was slow to respond to treatment and prone to recurrence. A familial background could be shown^129^ and careful breeding may have led to the perceived reduced frequency of this syndrome over the last 10 years. Until specific characteristics that are helpful for the diagnosis and management are identified, deep pyoderma in a GSD should be treated in the same way as deep pyoderma in any other breed.

### Diagnosis

Deep pyoderma is diagnosed based on the identification of compatible skin lesions such as draining tracts, haemorrhagic crusts, purple papules, ulceration or swelling and confirmation of a bacterial cause through cytological evaluation of representative areas. Clipping of long or matted hair may be needed to appreciate lesions. In some cases (e.g. ALD), signs typical of bacterial infection (e.g. pus, pustules) may be less obvious and deep pyoderma needs to be investigated based on clinical suspicion.^56^ In some cases, invasive sampling from below an intact surface is needed, either by biopsy or by needle aspiration (Box 1).

Underlying primary causes include all those described for other pyodermas (see Section ‘Investigations into underlying primary causes’). Atopic disease is likely to be the most common cause, yet demodicosis, hypothyroidism, hyperadrenocorticism and neoplastic processes also should be considered, depending on the case presentation. Inquiry into the dog's environment is an integral part of the diagnostic process in cases of ALD, callus pyoderma and interdigital infected nodules.

With paw lesions, examination (including palpation) of the palmar/plantar aspects of the affected paw(s) is critical to identify fused pads or comedones on weight‐bearing, haired surfaces. Identification of excess body weight or obesity and of conformational or gait abnormalities or joint disease (via orthopaedic examination and potentially including imaging if infectious osteomyelitis is suspected) is recommended.

#### BC/AST for deep pyoderma

*Staphylococcus pseudintermedius* remains the major pathogen and accounts for approximately 60% of pathogens isolated from deep pyoderma, which is in contrast to the >90% isolated from superficial pyoderma. Nevertheless, infections involving Gram‐negative organisms (e.g. *E. coli*, *Pseudomonas* spp.) or mixed bacterial populations are not uncommon. Although anaerobic pathogens are rarely reported, an anaerobic culture should always be included when processing samples from cases of deep pyoderma (Table 10).

**TABLE 10:** Bacterial pathogens most commonly reported from dogs with deep pyoderma (Web of Science, search string on 19 April 2022 using the string: Dog AND (furunculosis OR deep pyoderma OR cellulitis OR interdigital pyoderma OR acral lick) AND (culture OR microbiology OR aetiology OR bacteria). The last three papers in the table were retrieved from other sources.

Reference	Number of		Deep pyoderma type	Staphylococci		*Streptococcus* spp.	*Enterococcus* spp.	*P. aeruginosa*	*E. coli*	Other
	Isolates	Dogs		SP	Other					
Mueller & Stephan 2007^130^	231	135	Furunculosis, cellulitis, interdigital nodules	115		19	10	20	23	44^a^, ^b^
Shumaker et al. 2008^56^	36	30	Acral lick dermatitis	22	6	0	0	3	0	5^c^, ^d^
Restrepo et al. 2010^131^	8	6	Unspecified deep pyoderma	6	0	2	0	0	0	0
Gutierrez et al. 2020^132^	99	55	Unresponsive deep pyoderma	35	43	7	0	6	4	4^h^
Cain et al. 2015^133^	18	13	Dorsal furunculosis	1	3		0	10	0	4^e^, ^f^
Marchegiani et al. 2019^134^	61	36	Interdigital nodules	26	6	18	8	0	0	3,^g^
Špruçek et al. 2007^135^	10	10	Unspecified deep pyoderma	7	0	1	0	0	1	1,^i^
Total	428	285		270 (63%)		47 (11%)	18 (4%)	39 (9%)	25 (6%)	39 (9%)

Abbreviation: SP, *Staphylococcus pseudintermedius*.

^a^ *Enterobacter* (*n* = 6).

^b^ *Pantoea* (*n* = 9), *Acinetobacter* (*n* = 5) and *Pasteurella* (*n* = 4).

^c^ *Enterobacter* (*n* = 3).

^d^ *Clostridium perfringens* (*n* = 1).

^e^ *Enterobacter cloacae* (*n* = 1), *Serratia marcescens* (*n* = 1), *Klebsiella oxytoca* (*n* = 1).

^f^ *Burkholderia cepacia* (*n* = 1).

^g^ *Bacillus* (*n* = 3).

^h^ *Klebsiella* (*n* = 4).

^i^ *Actinomyces* (*n* = 1).

This higher pathogen diversity in deep pyoderma has important implications for antimicrobial therapy because some antimicrobials recommended for treatment of staphylococci‐associated pyoderma are inactive (e.g. owing to inherent resistance to lincosamides) or have limited efficacy against certain Gram‐negative bacteria such as *Pseudomonas* or AmpC‐producers (e.g. amoxicillin‐clavulanate). For other drugs, including cefalexin and other first‐generation cephalosporins, tetracyclines (doxycycline) and cefovecin, clinical breakpoints are approved for testing Gram‐negative bacteria from dogs, yet most isolates will be reported as resistant because concentrations achievable with standard doses are insufficiently high. Therefore, some laboratories may not include these agents in a culture report (each laboratory has the discretion to decide what agents to include in a report). This highlights the importance of submitting samples for BC/AST to laboratories that follow recognised standards (see Section ‘How to interpret BC/AST reports from dogs with pyoderma’).

Samples for BC/AST must be representative of the deep infection as surface swabs will often miss the relevant pathogens (see Section ‘Bacterial culture and antimicrobial susceptibility testing (BC/AST)’ and Table 4). In order to maximise the benefit of BC/AST results, cytology should always be performed from the same lesion so that culture results can be matched to the predominant pathogen morphology (e.g. cocci, rods or mixed) as seen cytologically, and thus inform the best drug choice (see Section ‘Cytological examination’).

### Treatment recommendations for deep pyoderma

#### Antimicrobial therapy

Systemic antibacterial therapy is always indicated in deep pyoderma, and the choice of drug should always be based on BC/AST results.

Culture‐based prescribing is particularly important in deep pyoderma to reduce the risk of treatment failure of **systemic antimicrobials** and of severe clinical consequences. Furthermore, owing to the long treatment periods typically required for deep pyoderma, the cost of laboratory testing will most often be counterbalanced by the cost of potentially ineffective empirically chosen medication.

Our literature search found 33 published clinical trials involving dogs with deep pyoderma (Table S3) and another 16 investigating dogs with deep and superficial pyoderma (Table S4). Systemic antimicrobial therapy had been evaluated in all, with adjunctive topical therapy in 16 (either as part of the study design or allowed to be continued from before).

Provided that BC/AST has indicated susceptibility, first‐choice drugs (Table 14) should be considered before second‐choice agents (Table 15). Choices will also be guided by clinical characteristics (accumulation in skin, penetration into inflamed tissue, activity in pus, safety, previous adverse drug reactions in the individual dog), and general considerations such as formulation availability, dosing frequency and cost.

Systemic antimicrobials are best started when BC/AST results are available. However, if there is a high risk of deterioration or septicaemia, empirical treatment guided by cytological findings can be started immediately, with re‐evaluation of drug selection when laboratory results are received.

Adjunctive topical antimicrobial therapy is recommended in every case as soon as the dog is considered pain free (SOR C).

Although no studies specifically investigated the effect of **adjunctive topical antimicrobial therapy** in deep pyoderma, adjunctive topical antimicrobials were used in 10 of the 30 published deep pyoderma studies, at least for some of the dogs (Table S3). Topical therapy is expected to facilitate healing by removing crust, reducing the microbial load on the surface, reducing pathogens and possibly shortening the duration of systemic therapy required. For the selection of active ingredients, product formulations and tips on maximising efficacy, see Section ‘Topical antimicrobial therapy’.

#### How long to treat deep pyoderma and when to stop systemic antimicrobials

An initial 3‐week course may be dispensed, and an appointment for re‐examination by a veterinarian should be scheduled before the end of the course to determine whether systemic treatment can be stopped or whether longer treatment is required.

If clinical signs are improving but have not resolved, and if cytological evidence of infection is still present, treatment should be continued with re‐evaluation every 2 weeks.

Systemic antimicrobial therapy can be stopped when skin lesions associated with deep infection (draining/fistulous tracts, pus, pustules, crusts) have resolved and there is no cytological evidence of infection.

At re‐examination, if draining/fistulous tracts, pus, pustules, ulcers or crusts remain or if lesions are no longer improving (have plateaued), their bacterial nature should be confirmed again by cytology to differentiate ongoing infection from sterile disease processes; if bacteria are seen on cytology, BC/AST need to be repeated to investigate drug resistance.

Topical antiseptic treatment can be continued beyond stopping systemic therapy where the primary underlying cause(s) cannot be resolved and the risk of recurrence remains.

There is no evidence to support extending systemic antimicrobial therapy beyond the resolution of clinical signs; instead, underlying primary causes must be identified and addressed.

High‐quality data from clinical treatment trials in canine deep pyoderma are sparse and conclusions on best treatment duration are limited by substantial variation in study design (Tables S3 and S4). Traditionally, a minimum of 4 weeks, extending to 8–12 weeks and 2 weeks beyond clinical cure were recommended.^2^, ^16^ Several months of antimicrobial treatment have been mentioned for the management of ALD.^136^ The rationale behind longer courses for deep pyoderma was to avoid sequestered, subclinical foci of infection causing relapse, yet evidence to support this practice is lacking. With these long treatment recommendations under scrutiny and following our literature review, our group's consensus was to shorten the recommendations by advocating 3 weeks as the initial duration, supported by adjunctive topical antimicrobials and scheduled re‐examinations every 2 weeks to monitor progress. Re‐examinations provide an opportunity to stop, extend or adjust treatment depending on progress, to review compliance and safety of treatment, and to investigate and manage the underlying primary causes. Where a re‐examination before the initial 3 week course is not feasible, treatment can be extended to avoid stopping treatment before veterinary assessment.

At re‐examination, when lesions no longer improve, previously affected areas should be sampled for cytology again (Table 4) to establish whether they are still caused by bacterial infection. If intracellular bacteria are still seen in cytology findings and clinical progress has stopped, another sample should be taken for BC/AST to investigate drug resistance. If appropriately taken samples no longer show cytological evidence of bacterial infection, persistent lesions may be attributed to underlying primary, potentially chronic disease and sterile diseases (Clinical case scenario in Section ‘Clinical case scenario’).^137^, ^138^

The consensus is that it is very rare that >6 weeks of appropriate antimicrobial therapy will be required to eliminate the secondary pyoderma component of skin lesions. Although it will remain difficult to show that bacteria are no longer contributing to remaining lesions (e.g. where remnants of dead *Demodex* mites or hair‐shaft‐induced cysts arising from the palmar/plantar paw surfaces continue to drive inflammation), anti‐inflammatory medications such as prednisolone or ciclosporin, as clinically appropriate for the case, should be considered to replace antimicrobials (Clinical case scenario in Section ‘Clinical case scenario’). Healing of chronic lesions with scarring (fibrosis) may leave affected skin permanently alopecic and more fragile, yet further improvement and prevention of recurrent infection will not require systemic antimicrobials.

#### Supportive management

Medication to **relieve pain** should be considered if the dog is likely to be in pain.

In order to support the resolution of infection during antimicrobial treatment and to prevent recurrence after infection has resolved, **underlying primary causes** must be found and controlled. In many cases, this will be a bigger challenge than resolving the pyoderma component. However, the importance of resolving the underlying primary cause should not be underestimated as individual cases of deep pyoderma have even been shown to improve without systemic antimicrobial therapy. In one study, 16 dogs with generalised demodicosis and secondary bacterial furunculosis achieved complete resolution with acaricidal treatment and benzoyl peroxide washes alone^139^; guidelines for the treatment of demodicosis have been published.^140^ Likewise, a single case of severe postgrooming furunculosis, confirmed histologically, is described to have fully recovered with once daily anti‐inflammatory doses of prednisolone alone.^133^

Adjunctive **fluorescence biomodulation** has been shown to shorten the time to lesion resolution in dogs with deep pyoderma and interdigital furunculosis^134^, ^141^ and it has also been reported to have led to resolution of MDR pyoderma in 16 dogs.^142^ This effect may not be the result of a direct antimicrobial action of the light therapy and, rather, of other mechanisms that support healing and restoration of skin barrier function.^143^

Whirlpool therapy was historically and anecdotally recommended as adjunctive therapy to promote healing, yet following the emergence of multidrug‐resistant pathogens, infection control measures would need to be rigorous.

**Management changes** should be tailored to the disease presentation, underlying primary cause(s), body site and practicality for owners. For example, providing softer surfaces for play and rest and facilitating weight loss in overweight dogs may reduce friction and chronic trauma to vulnerable and affected areas (Box 7).

BOX 7 **Example treatment approach to a dog with widespread deep bacterial folliculitis‐furunculosis**

1

- Dispense 3 weeks of systemic antimicrobial therapy, selected based on bacterial culture and antimicrobial susceptibility test results (prioritising first‐choice drugs over second‐choice drugs if susceptibility is indicated)
- Adjunctive topical antimicrobial therapy (e.g. 2%–4% chlorhexidine shampoo, solution (spray) or mousse) to be applied every 24–48 h
- If the dog is in pain, consider adequate pain‐relief medication
- Identify, investigate and address underlying primary causes
- Re‐assess progress before the end of the 3‐week course:
  - If all skin lesions have resolved: stop systemic antimicrobials
  - If lesions are improving but lesions related to pyoderma remain (e.g. diffuse swelling, papules, pustules, furuncles or pus can still be expressed) **and cytology still reveals intracellular bacteria**: continue systemic antimicrobials for another 2 weeks and schedule re‐examination in 2 weeks (before the end of the course). This step can be repeated every 2 weeks until resolution of infection.
  - Consider whether anti‐inflammatory drugs (e.g. glucocorticoids or ciclosporin) are indicated and clinically appropriate to replace antimicrobials and support healing (see Clinical case scenario 6.4)
  - If the lesions have not improved or worsened, (1) reassess compliance, (2) take skin biopsies for histopathological evaluation and BC/AST and (3) review investigations into underlying causes
- Ensure that a long‐term plan for correcting or managing underlying primary causes is in place before stopping topical antiseptic therapy.

### Clinical case scenario

**FIGURE 9:**
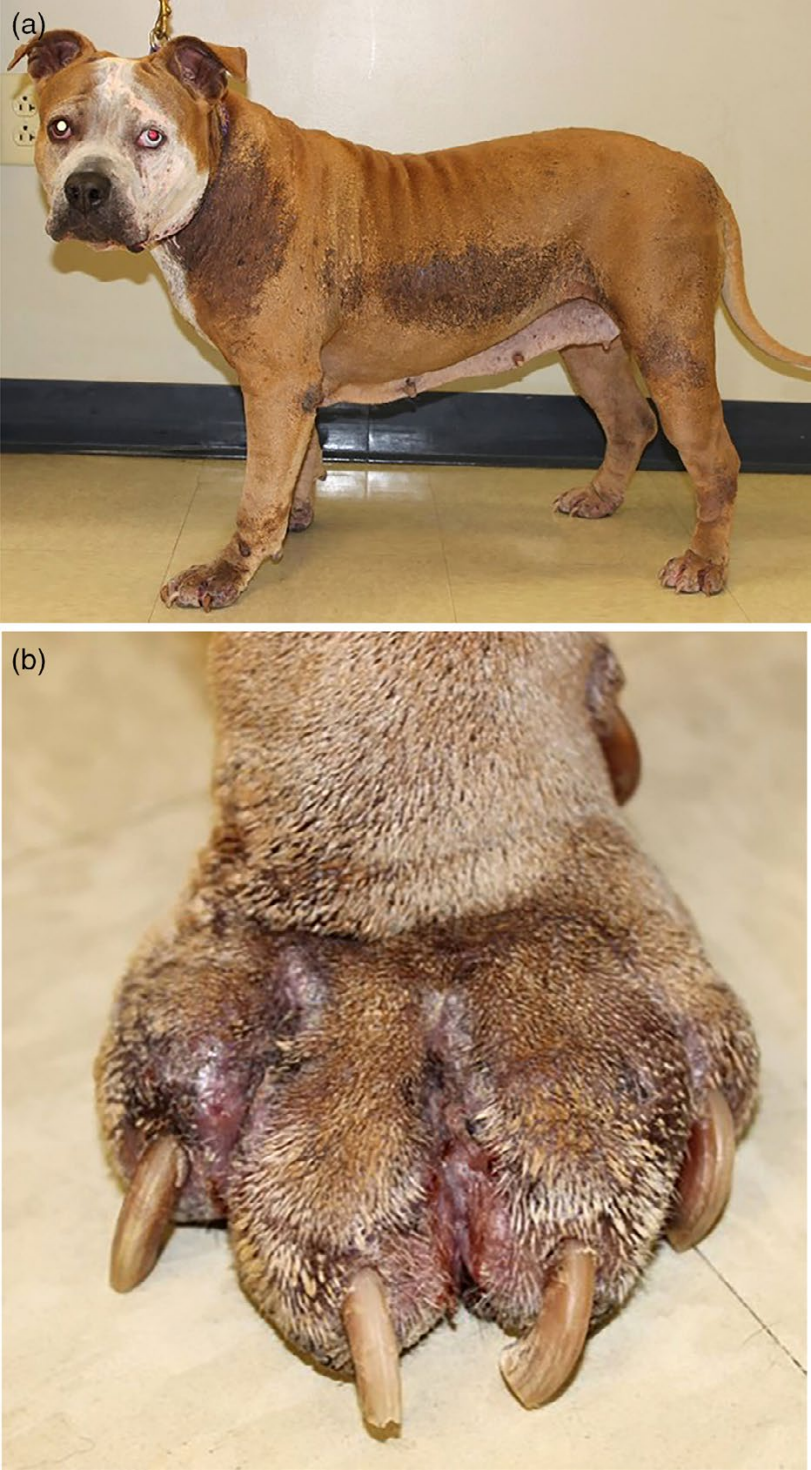
Mastiff cross with deep pyoderma secondary to allergic skin disease. (a) Widespread alopecia and hyperpigmentation affecting the body. (b) Severe suppurative pododermatitis on all four paws.

A 4‐year‐old, spayed female Mastiff cross presents with a 2.5‐year history of generalised pruritic dermatitis. Improvement had always been seen with intermittent treatment using oral prednisone, oclacitinib and systemic antimicrobials (cefalexin, amoxicillin‐clavulanate), yet skin lesions always recurred when treatment was stopped. At presentation, she showed signs of pruritus, symmetrical areas of alopecia and hyperpigmentation on the face, neck and flanks (Figure 9a) and alopecia, erythema, papules, pustules and suppurative fistulous tracts on all four paws (Figure 9b) with signs of pain when walking.

Microscopic examination of plucked hairs (trichogram) showed no evidence of *Demodex* mites or fungal elements. Wood's lamp examination also was negative. Cytology from impression smears revealed pyogranulomatous inflammation with the presence of intra‐ and extracellular cocci. A diagnosis of deep pyoderma (folliculitis‐furunculosis) secondary to allergic dermatitis (atopic dermatitis or adverse reaction to food or both) was made.

After careful cleaning of the surface of a paw lesion, first with water and then with 70% alcohol, a swab for BC/AST was obtained from the exudate discharging from the interdigital fistulous tracts. This yielded an abundant, pure growth of a meticillin‐resistant *S. coagulans* (MRSC), resistant to all antimicrobials in the first‐choice group. Susceptibility was reported to all fluoroquinolones tested, and enrofloxacin (10 mg/kg per os once daily) was prescribed. Additionally, an oral isoxazoline, pain relief medication (7 days), and 3% chlorhexidine whole‐body washes every 48 h were prescribed, more frequently for the paws.

Three weeks later, all suppurative interdigital lesions had healed, although areas of alopecia and scarring remained. Enrofloxacin treatment was discontinued, yet it was recommended to continue with two chlorhexidine baths per week. A food trial was declined, so it was recommended to start treatment with ciclosporin 5 mg/kg p.o. once daily for the long‐term control of the allergic dermatitis. At re‐examination 2 months later, all skin lesions had resolved and no signs of recurrence had been noted. Ciclosporin therapy at the lowest necessary frequency combined with once or twice weekly chlorhexidine washes was recommended to be continued long‐term.

## 8 TOPICAL ANTIMICROBIAL THERAPY

### General comments

Topical therapy used as sole antimicrobial treatment is the treatment‐of‐choice for all cases of surface and superficial pyoderma (SOR A) and also should be considered as adjunct therapy in all cases of pyoderma that require systemic treatment.

Topical antimicrobial therapy provides a great opportunity for responsible antimicrobial stewardship when managing patients with pyoderma (Box 8).

BOX 8 **Advantages of topical antibacterial treatment**

1 Reduces need for/use of systemic antimicrobials and reduces impact of systemic antimicrobial therapy on gut microbiome, as well as other adverse events.

Effective in the treatment of surface and superficial pyoderma; can replace systemic antimicrobials.

When used as adjunctive therapy, it may reduce the duration of systemic therapy needed.

Effective against both meticillin‐susceptible and meticillin‐resistant staphylococci.

Can rapidly reduce environmental contamination by MRSP, MRSA, MRSC by reducing carriage on squames and hair.

Low risk of driving antimicrobial resistance when used appropriately owing to high local concentration of the active ingredient at the site of infection.

Safe, usually well‐tolerated and suitable for long‐term use.

Often cost‐effective.

Shampoo or wipe formulations: reduce malodour, scale and crust, number of bacteria and biofilm and increase cleanliness.

For best outcomes, clinicians need to choose an active ingredient(s) effective against the causative microbes and a product formulation suitable for the clinical presentation and for the dog–owner combination.

### Which active ingredient?

Topical antimicrobials are from diverse groups of agents with sometimes overlapping definitions (Table 11).

**TABLE 11:** Selected terms and definitions used in this document in the context of topical agents that can inhibit or kill bacteria.

Group	Definition	Example
Antibiotic	Derived from a micro‐organism. Some antibiotics used topically also may be used systemically, yet possibly not in all species.	Fusidic acid, mupirocin, polymyxin B
Antimicrobial	Produced chemically or derived from a micro‐organism.	Silver sulfadiazine
Antiseptic	Applied to living tissue, for example to skin.	Chlorhexidine
Biocide	Diverse group, sometimes including disinfectants, but also pest control products or preservatives.	Various
Disinfectant	Used on inanimate objects. Some are used in much lower concentrations as antiseptics.	Sodium hypochlorite (bleach)

Antiseptics should be prioritised over topical antibiotics. Topical antibiotics should be reserved for cases that have not responded to antiseptics or if antiseptics are deemed inappropriate (e.g. known hypersensitivities). Although evidence for this concept is lacking at present, using topical antibiotics prudently may preserve their efficacy for systemic treatment in animals or humans.

A large body of data has been published showing good in vitro anti‐staphylococcal activity for many topically used agents, while comparatively fewer clinical treatment studies are available on efficacy in vivo, such as in the presence of hair, organic material, and biofilm. Active ingredients for which clinical studies were found during our systematic literature review are listed in Table 12 (and Table S1). Additionally, some agents not listed in the tables may be of use for the management of bacterial skin infections based on in vitro support, non‐English language studies, surgical scrub models or anecdote. Alternative agents with potential for topical antimicrobial therapy in canine pyoderma are undergoing testing and may become available for clinical use in the future.^144^

**TABLE 12:** Recommendations for use of selected active ingredients as topical antibacterial therapy in canine surface and superficial pyoderma based on evidence for efficacy from in vivo studies in dogs with spontaneous surface or superficial pyoderma identified during a systemic literature review.^11^

Group	Active ingredient	Strength of recommendation (SOR) for use as a sole therapy in:		Comments
		Surface pyoderma	Superficial pyoderma	
Antiseptic	Benzoyl peroxide 2.5%	SOR A^64^	Not recommended^96^	Can be irritant
	Chlorhexidine 2%–4% (possibly less effective against *Pseudomonas* spp. or ESBL‐producing *E. coli*)^146^	SOR A^64^, ^76^	SOR A^89^, ^94^, ^97^, ^98^, ^99^	Also antifungal
	Chlorhexidine 2%/Miconazole 2%	–	SOR A^98^	Both agents are anti‐staphylococcal and antifungal
	Olanexidine	–	SOR A^94^	Molecule related to chlorhexidine
	Sodium hypochlorite (NaOCl, bleach)/salicylic acid	–	SOR B^93^	Commercial preparation, concentration unknown
Topically used antibiotics	Fusidic acid	SOR A^25^	–	May be restricted in some geographic areas
	Neomycin	SOR B^59^	–	One RCT showing neomycin alone inferior to glucocorticoid alone
Other	Carbonated water	–	SOR B^95^	RCT yet small number of dogs
	Erythritol (5%)/0.1% l‐ascorbyl‐2‐phosphate		SOR B^101^	Prospective case series in 10 dogs
	Medical honey	Not recommended (no antibacterial effect compared to placebo)^75^	–	Summers et al.^8^ reported limited evidence for efficacy in intertrigo based on a report published in Swedish.

While the clinical role of biofilm produced by *S. pseudintermedius* ^145^ in pyoderma remains unknown, physical disruption of potential biofilms and removal of surface debris using water and wipes or shampoos is likely to improve the efficacy of topical antimicrobials.

#### Antiseptics

Currently, the agent with the greatest support for efficacy in canine pyoderma is chlorhexidine at 2–4%, available and found effective in several compositions and formulations. Chlorhexidine in the form of a 4% shampoo and spray used twice weekly was shown to be equally effective as systemic antimicrobial therapy in 31 dogs with superficial pyoderma over a 4‐week period.^89^ Individual studies have compared different chlorhexidine formulations (surgical scrub, shampoo, solution), concentrations and/or administration frequency, finding little statistical difference between groups.^97^, ^98^, ^99^ However, in vitro evidence suggests that low‐concentration chlorhexidine products may only have low efficacy against ESBL‐producing *E. coli* and no efficacy against *Pseudomonas* spp.^146^ This should be considered if it is likely or known that these organisms are present or if rods predominate in cytology findings. Good‐quality evidence for efficacy also was found for a chlorhexidine/miconazole combination, benzoyl peroxide and olanexidine at least for one type of pyoderma (Table 12).

Support for the use of a dilute sodium hypochlorite (NaOCl; bleach)/salicylic acid combination product (concentrations unknown) was published in 17 dogs with staphylococcal pyoderma where bacterial counts and clinical scores improved with three‐times weekly washes over 4 weeks.^93^ Household bleach is widely available as a domestic disinfectant in concentrations of 3%–8% (strength at time of manufacturing) or of 1.5% in disinfection wipes and sprays for use on household surfaces (not safe to use on skin in these concentrations!). It also is used at much lower concentrations of 0.05–0.005% in baths for humans with atopic eczema.^147^, ^148^ Although chlorine is an excellent bactericidal agent, its bactericidal effect in vivo remains questionable as contact with organic matter on skin has a degrading effect and may reduce activity. Household bleach requires significant dilution to achieve a concentration considered safe for use on dogs (<0.05%),^149^ creating a potential for mixing errors that could pose a risk to the dog or owner. Another chlorine‐related oxidising agent is hypochlorous acid (ClOH or HClO), which has been shown to be safe as an ear flush solution in dogs at a concentration of 0.015%.^150^ Hypochlorous acid is present within leucocytes of mammals as an endogenous antimicrobial; it is safe for use in open wounds and therefore suitable for the management of pyoderma lesions associated with erosions or ulcers.^72^

Other molecules used as antiseptics on skin include acids (e.g. acetic, boric, lactic and salicylic), ethyl lactate, 80% ethyl alcohol, povidone iodine, triclosan, some botanical extracts and metal solutions (e.g. aluminium triacetate solution, Burow's solution, magnesium sulfate, Epsom salt) and chloroxylenol (PCMX; used in household cleaning products and some over‐the‐counter dog shampoos). The antibacterial efficacy of these agents has been suggested by a combination of in vitro work, surgical scrub models, commercial household cleaning data, small clinical studies (not published according to our literature search criteria) and anecdote; however, owing to a lack of clinical efficacy and safety data, these agents cannot be recommended at present for clinical use in canine pyoderma while alternative products with proven efficacy and safety are available (Table 12).

#### Topical antimicrobials or antibiotics

Some systemically administered antimicrobials (e.g. fusidic acid) also are available as topical formulations, while others are only available for topical therapy (e.g. mupirocin) (Table 12). Although laboratory breakpoints for these agents exist, including for isolates from animals, susceptibility test results reported by laboratories are only meaningful for systemic therapy and do not predict efficacy by the topical route.

Fusidic acid and mupirocin are known for their narrow‐spectrum anti‐staphylococcal activity. Although there are currently no clinical data on fusidic acid when used alone in canine pyoderma, its efficacy when used as a topical combination product with betamethasone was shown to be equal to that of systemic amoxicillin/clavulanic acid and dexamethasone in dogs with pyotraumatic dermatitis.^25^ In addition, an ex vivo study showed that fusidic acid, applied as a 10‐mg/g ophthalmic suspension, can penetrate to the level of the hair follicle infundibulum at concentrations that greatly exceed typical MICs for canine *S. pseudintermedius*.^151^ In one abstract presentation, clinical efficacy of a 0.2% mupirocin spray was suggested to be comparable to a 4% chlorhexidine mousse, when both were applied twice daily for 3 weeks, in reducing lesion and pruritus scores in five dogs with pyoderma (depth not stated).^152^

For other topical antibiotics such as bacitracin, florfenicol, neomycin, nisin, polymyxin B and silver sulfadiazine, weak evidence in canine pyoderma has been published for some (Table S1). Their use may be preferable over systemic antimicrobials in surface and superficial pyoderma where agents listed in Table 12 are not suitable, yet they cannot be recommended with support from published clinical evidence and there is currently no evidence suggesting a benefit over topical chlorhexidine. For some localised, inflamed lesions, for example in surface pyodermas, topical antibiotics may be advocated and effective in the form of multi‐pharma ear drop formulations (off‐licence).^70^

#### Adverse reactions and resistance concerns

Topical antimicrobial formulations can be considered safe if used according to manufacturers' or literature‐recommended instructions and provided no known hypersensitivities are reported. Nevertheless, adverse reactions such as erythema and pruritus can be seen, usually immediately after application. They tend to be transient and do not require specific treatment. Contact dermatitis can rarely occur after repeated application. In cases where reactions are problematic, changing the formulation and active ingredient or reducing the concentration may be advisable. Chlorhexidine solutions are toxic to the cornea and use around the eyes, including where there is potential for splashes, should be avoided.^153^ For treatment of periocular skin lesions, antimicrobial ophthalmic preparations may be safer. The potential for cutaneous atrophy when using topical glucocorticoid combinations over long terms needs to be considered.

Resistance or nonsusceptibility to topically used agents has not been convincingly documented or defined to date. Although genes associated with higher MICs to some topical agents have been identified in bacterial pathogens, their presence has not been linked to clinical treatment failure. MICs of staphylococci from dogs have been shown to be low against fusidic acid and chlorhexidine.^154^, ^155^, ^156^

Although there have been intermittent reports of in vitro chlorhexidine tolerance in staphylococci, the concentrations of chlorhexidine achieved through appropriate clinical use far exceed the maximum MICs described in the literature, despite decades of use of this active ingredient. No true antimicrobial resistance resulting in clinical treatment failure of staphylococcal infection using chlorhexidine has yet been described.

### Product formulation

In order to maximise chances of active ingredients reaching the site of infection for the required duration, at the desired concentration, and at the necessary frequency, a practical product formulation needs to be chosen (Table 13). Shampoos are well suited for widespread disease, while nonrinse preparations may be preferable as they are less time‐consuming. Mousses, wipes or sprays may be favoured by some for their suitability for skin fold disease. In addition to their immediate antibacterial effect, some shampoo, spray and mousse/foam formulations provide residual antimicrobial activity on hairs for ≤10 days.^157^, ^158^, ^159^

**TABLE 13:** Product formulations available for topical antimicrobial therapy in canine pyoderma.

	Formulation	Most suitable	Comments	Typical frequency recommended for initial therapy
Leave‐on application, no rinsing	Cream	Localised lesions (e.g. acute moist dermatitis, skin folds)	If in combination with a glucocorticoid, advise owners to wear gloves and limit duration of application (particularly to axillary/inguinal regions) to minimise cutaneous atrophy	Once or twice daily (or more often if ingredients are only antimicrobial)
	Gel			
	Ointment			
	Suspension			
	Solution			
	Foam or mousse	Localised or widespread, interdigital skin	Consider when frequent bathing is not possible	Daily
	Spray	Localised lesions	May be suitable for painful areas Easy and clean to apply	Once or twice daily
	Wipes	Skin folds, localised lesions	Easy to use in folds, practical and effective for less‐haired skin	Once or twice daily
	Soaks	Widespread lesions, pododermatitis		Daily
Rinsing needed	Shampoo	Widespread lesions in hairy areas (clipping of long coats will aid application and penetration)	Considerable time and effort required May interfere with the efficacy of other topically applied products—consider changing to oral ectoparasiticides	Typically at least two to three times per week Contact time of 10–15 min recommended

### Practical tips to promote good outcomes

Many owners will be familiar with the concept of topical therapies as they are widely used in human medicine for the management of skin conditions. However, the application of topical therapy to hairy, sometimes uncooperative, dogs several times daily can be difficult and often more challenging than systemic treatment. The risk of compliance failure can be minimised by highlighting some of the advantages of topical therapy to enhance acceptance or ‘buy‐in’ for topical treatments and treatment success (Box 8).

Strategies to improve the success of topical therapy can include the following:

- Clipping of long, matted or thick hair coats can make application easier.
- Removing excess debris using water and wipes before the application of topical products, as active ingredients may be inactivated by organic material including biofilm.
- Ensuring contact of product with lesional skin
- Allowing adequate contact time for shampoo formulations; a 10‐ to 15‐min contact time before rinsing is currently recommended.
- Tailoring of product formulations to dog/lesion and owner needs (Table 13)
- Combining different product formulations (same or similar active ingredient) may improve acceptance; for example, replacing shampoos on some days with a mousse/foam, spray or solution that do not require wetting and rinsing off.
- Distracting dogs from removing active ingredients through play, walks or feeding immediately after treatment

Careful instructions on shampooing and soaking, application of sufficient product, concentration, contact time and rinsing may be needed. One study determined the amount of 2% chlorhexidine acetate shampoo needed for bathing a two‐hand‐sized area of infected skin to be the size of a 26.5‐mm diameter coin when dispensed.^99^ Manufacturers of topical products are encouraged to provide specific dosing recommendations (e.g. pumps per kg body weight or m^2^) to ensure maximum efficacy.

Some topical therapy combinations may be offered by compounding pharmacies. These are typically compounded (formulated) using bulk chemical substances and other ingredients. Compounded products are not approved by regulatory authorities and the quality, strength, safety and effectiveness cannot be assured. Some regulatory authorities prohibit compounding from bulk chemical substances (e.g. https://www.fda.gov/regulatory‐information/search‐fda‐guidance‐documents/cvm‐gfi‐256‐compounding‐animal‐drugs‐bulk‐drug‐substances).^160^ Whenever possible, use approved products.

## 9 SYSTEMIC ANTIMICROBIAL THERAPY FOR CANINE PYODERMA

### General comments

Systemic antimicrobials should be reserved for deep pyoderma or for a small number of cases of superficial pyoderma where topical therapy alone has not been effective or is not feasible (Figure 1).

For this version of the canine pyoderma guidelines, we assigned antimicrobial drugs to four groups in order of selection priority: first‐choice, second‐choice, reserved and strongly discouraged (Tables 14, 15, 16, 17). Groupings reflect the authors' considerations of efficacy and safety based on published evidence and the importance of a drug in human medicine. For our document, drug allocations were created following an iterative process and unanimously agreed upon by our author panel except for the potentiated sulfonamides. This drug class was moved from the first‐choice group into the second‐choice group by majority vote, not for resistance concerns but owing to concerns over clinical adverse events for dogs. Another drug assigned to a different group compared to the earlier ISCAID guidelines for canine bacterial folliculitis^5^ is cefovecin, which has been placed into the second‐choice group by unanimous vote. This change is in line with datasheet recommendations in most countries. These state that the use of third‐generation cephalosporins should be based on susceptibility testing.

**TABLE 14:** ‘First‐choice drugs’ for canine pyoderma if systemic therapy is indicated.

First‐choice drugs: good, predicted efficacy against most meticillin‐susceptible *Staphylococcus* spp., low risk of adverse effects			
Class	Drug & suggested dose	Comments & evidence base in canine pyoderma	Resistance considerations
β‐lactams	Amoxicillin‐clavulanate 12.5 mg/kg p.o. q12h^115^	Although used at higher doses anecdotally, evidence to support higher doses is lacking. Recommended dosing intervals should be considered for a time‐dependent drugs. Clinical studies: 12 studies involving 338 dogs using different outcome measures: Variable evidence for efficacy (Tables [Link], [Link])	Can increase faecal shedding of ampicillin‐resistant *E. coli* during treatment^169^, ^170^
	Cefalexin, cefadroxil^171^ 22–25 mg/kg p.o. q12h	First‐generation cephalosporins Cefadroxil and cefalexin may be used interchangeably with equal efficacy depending on availability In vitro: Excellent activity against meticillin‐susceptible *Staphylococcus pseudintermedius* ^172^ Clinical studies: 22 studies involving 555 dogs using different outcome measures: Variable evidence for efficacy reported for both drugs (Tables [Link], [Link])	Can increase faecal shedding of *E. coli* producing CMY‐2 β‐lactamase in dogs^173^, ^174^
Lincosamides	Clindamycin 11 mg/kg p.o. q12h	Although there is evidence for successful treatment when dosed at 5.5 mg/kg p.o. q12h, we recommend a higher dose for a more consistent clinical response Narrow spectrum, high activity against wild‐type strains of *Staphylococcus* spp. and with regional variation in activity; check local resistance pattern before empirical use Clinical studies: Efficacy reported by individual studies	Should not be used in erythromycin‐resistant isolates without additional testing, owing to inducible clindamycin resistance seen in some *S. aureus* isolates and rarely in *S. pseudintermedius* isolates^175^
	Lincomycin 22 mg/kg p.o. q12h	May be used as substitute for clindamycin (if unavailable) Insufficient evidence for or against use of lincomycin Oral formulations suitable for accurate dosing in dogs may be geographically restricted	No approved susceptibility testing standards and breakpoints available for veterinary isolates

Abbreviations: p.o.: per os (administered orally); q12h: twice daily every 12 h.

We steered away from the terms ‘tier’, ‘line’ and ‘category’ to avoid confusion with other classification systems. These systems are used to reflect the likelihood of an agent to produce resistant bacteria that can be a public health risk and help to prioritise low risk drugs over those of critical importance for human health. Unfortunately, a degree of confusion has developed with the wider use of ranking systems. For example, the ‘first tier’ classification by the US Food and Drug Administration (FDA)^161^ relates to the most critical and important drugs for human health whereas the ‘first tier’ or ‘first line’ category in earlier veterinary pyoderma guidelines^5^, ^162^ comprises the ‘preferred use’ agents.

Drugs authorised for use in canine pyoderma should be used whenever possible. Authorisation processes will have involved efficacy and safety testing, which will improve chances of successful outcomes. Recommended dosages are expected to achieve therapeutic drug concentrations against *S. pseudintermedius* in well‐perfused canine skin. The recommended doses are in agreement with those listed in Appendix D of the CLSI veterinary standards as well as pharmacokinetic‐pharmacodynamic (PK‐PD) assessment.^48^

General concepts of antimicrobial prescribing, such as frequent weighing of the dog, accurate dosing and reminding owners of the need for good compliance, are important. Maintaining regular administration intervals is particularly important for time‐dependent antimicrobials such as the β‐lactams, while doses at the high end of the dosing range are recommended for concentration‐dependent drugs such as the fluoroquinolones.

Drugs in this document are spelt according to the International Nonproprietary Name (INN) (e.g. cefalexin, rifampicin) which may differ from other drug nomenclature such as the United States Adopted Names (USAN) (cephalexin, rifampin).

### Culture‐based treatment

For MSSP, first‐choice drugs should be used unless clinical considerations (not simply convenience) make agents from this group unsuitable (e.g. known drug hypersensitivities, a clinical or safety need for longer dosing intervals). If other drug options are needed, second‐choice drugs, including the potentiated sulfonamides (formerly listed as first‐choice drugs for pyoderma), can be considered provided that potential clinical adverse effects and an increased risk of selecting for drug resistance (e.g. fluoroquinolones)^163^ are taken into account.

If MRSP is isolated, first‐ or second‐choice drugs may still be appropriate if in vitro susceptibility is indicated and treatment with those agents can translate into clinical efficacy (see also Section ‘Meticillin‐resistant staphylococcal pyoderma’). However, for multidrug‐resistant MRSP, agents from the reserved drugs group might be needed.

If BC/AST yields mixed bacterial growth with varied susceptibility patterns, its individual relevance should be assessed (see Section ‘Bacterial culture and antimicrobial susceptibility testing (BC/AST)’).

### Empirical choices

Empirical systemic antimicrobial therapy should only be considered where the risk for drug resistance is low.

Only first‐choice drugs (clindamycin, cefalexin, cefadroxil, amoxicillin‐clavulanate) should be considered for empirical therapy.

First‐choice drugs are expected to have good efficacy against MSSP (SOR A).

Drug choices based on BC/AST results will always be preferable, yet empirical choices for pyoderma treatment may be appropriate if cytology findings support a staphylococcal infection and if there is a low suspicion for MRS based on clinical, historical and geographical information. This applies to some cases of uncomplicated infections that are likely to resolve quickly, those that have not received systemic antimicrobial therapy previously for any reason (or very little) and to dogs that live in areas with a low prevalence of MRS.

If first‐choice drugs are unsuitable (e.g. known drug hypersensitivities, clinical or safety reasons requiring longer dosing intervals), BC/AST should inform the selection of second‐choice or other group drugs.

Because cost (and occasionally practicality) is likely to be the main driver for empirical selection of drugs, owners should be made aware that the cost of antimicrobial resistance may ultimately exceed that of BC/AST, should empirically chosen drugs turn out to be ineffective; likewise, practices and laboratories should support antimicrobial stewardship by charging reasonably for BC/AST.^164^ Resistance to some first‐choice agents among MSSP is higher in dogs that have previously received systemic antimicrobial therapy and in those presenting to referral centres.^165^, ^166^, ^167^, ^168^ The economic and clinical value of BC/AST also increases with the prevalence of meticillin‐resistance among *S. pseudintermedius* clinical isolates and in situations where the risk of MRSP involvement is high. Testing is considered critical in certain situations (Box 2), in dogs presenting to referral centres and those with a history of prolonged or repeated antimicrobial treatment, MRS infection, or with MRS infection in their household.

### First‐choice drugs

Drugs listed as ‘first‐choice drugs’ (Table 14) are expected to show good efficacy in dogs with pyoderma owing to meticillin‐susceptible staphylococci on the basis of approval by regulatory authorities (e.g. European Medicines Agency (EMA), FDA) and based on published clinical trial evidence.^8^

Representatives of first‐choice drugs are expected to be available and approved in most countries for the treatment of canine skin infections and susceptibility testing breakpoints for staphylococci from dogs are available for all except lincomycin.^48^ Dosages listed here may deviate from previous recommendations for staphylococcal skin infections in dogs and from registration datasheets. They are based on a careful review of the current approved label recommendations in various countries, updated PK‐PD analysis, and the dose that was used to determine approved susceptibility testing breakpoints (Appendix D of CLSI 2024 standards).^48^

### Second‐choice drugs

Drugs listed as ‘second‐choice’ should *only* be considered (i) when the causative bacterium is susceptible based on BC/AST results, *and* (ii) when first‐choice agents are not appropriate (Table 15). They are ranked as ‘second‐choice’ either because of an increased relative risk for the selection of important multidrug‐resistant pathogens in pets and humans^161^, ^176^, ^177^ or as a consequence of an increased risk of adverse events.^178^ Compared to first‐choice drugs, approval status, regulatory legislation and availability of these agents are more variable across the world.

**TABLE 15:** ‘Second‐choice drugs’ for canine pyoderma if systemic therapy is indicated.

Second‐choice drugs: only to be considered when BC/AST results are available, and when first‐choice drugs are not suitable			
Class	Drug & suggested dose	Comments & evidence base in canine pyoderma	Resistance considerations
Third‐generation cephalosporins	Cefovecin 8 mg/kg subcutaneously once	Only available as long‐acting injectable formulation Use should be limited to infections with *Staphylococcus pseudintermedius* ^181^ Can be repeated after 14 days if clinical progress has been confirmed yet some lesions compatible with infection remain Can be considered when compliance failure is a factor in achieving treatment cure In vitro and clinical evidence for efficacy against meticillin‐susceptible staphylococci is strong Only weak activity against Gram‐negative bacteria such as *Escherichia coli* and no activity against *Pseudomonas* spp.^181^ Restricted by regulatory authorities in some countries; consult local antimicrobial policies and restrictions	The European Medicines Agency Committee for Veterinary Medicinal Products (EMA CVMP) provides a *special precautions for use in animals statement* that ‘It is prudent to reserve third generation cephalosporins for the treatment of clinical conditions, which have responded poorly, or are expected to respond poorly, to other classes of antimicrobials or first generation cephalosporins.’ Staphylococci resistant to first‐generation cephalosporins, amoxicillin‐clavulanate and oxacillin/meticillin also will be resistant to cefovecin and to cefpodoxime proxetil
	Cefpodoxime (proxetil) 5–10 mg/kg p.o. q24 h	Activity against *Staphylococcus* spp. is similar to first‐generation cephalosporins Cefpodoxime once daily was equally effective as twice daily cefalexin^182^ Well‐tolerated after oral administration Both availability and usage may be geographically restricted; consult local antimicrobial policies and restrictions	
Second‐Generation Fluoroquinolones	Enrofloxacin 5–20 mg/kg p.o. q24 h	Although the label dose range is wide, a minimum dose of 10 mg/kg of enrofloxacin and of 5.5 mg/kg of marbofloxacin is recommended for *Staphylococcus* spp. (and 20 mg/kg of enrofloxacin for *P. aeruginosa)* High doses should be used if isolates are reported as ‘SDD’ (susceptible dose‐dependent by CLSI)^179^ Restricted by regulatory authorities in some countries; consult local antimicrobial policies and restrictions	There is some evidence that oral administration of a fluoroquinolone may be more likely to select for antibiotic‐resistant *E. coli* in dogs compared to other antimicrobial agents^183^, ^184^, ^185^ Fluoroquinolone use is one of the main risk factors for acquisition of MRSA in humans^158^, ^173^, ^183^, ^184^
	Marbofloxacin 2.75–5.5 mg/kg p.o. q24 h		
Third‐generation Fluoroquinolones	Orbifloxacin 7.5 mg/kg p.o. q24h	Availability may be geographically restricted	As for other fluoroquinolones: risk factor for MRS
	Pradofloxacin 3–6 mg/kg p.o. q24 h	Restricted by regulatory authorities in some countries; consult local antimicrobial policies and restrictions Clinical studies: efficacy shown in 20 dogs with either superficial or deep pyoderma^130^ and in 56 dogs with deep pyoderma in a controlled study^130^ High doses should be used if isolates are reported as ‘SDD’ (susceptible dose‐dependent by CLSI)^179^ Restricted by regulatory authorities in some countries; consult local antimicrobial policies and restrictions	As for other fluoroquinolones: risk factor for MRS May be preferable over other fluoroquinolones due to a lower risk of selection for resistance due to its low mutant prevention concentration shown in vitro^186^
	Levofloxacin^187^ 25 mg/kg p.o. q24 h	Human‐only fluroquinolone Almost 100% oral absorption in dogs and similar in activity to the veterinary approved fluoroquinolones Should only be considered as an alternative when veterinary approved agents are not available, or where the dog's size necessitates dosing with multiple tablets and this is not feasible owing to patient factors	Only fluoroquinolone with approved testing standards and breakpoints for *Pseudomonas aeruginosa* isolates from animals As above, risk factor for MRS
Tetracyclines	Doxycycline hyclate or monohydrate (can be used interchangeably) 5 mg/kg p.o. q12h or 10 mg/kg p.o. q24 h	Availability may be geographically restricted Good safety profile in dogs except for vomiting (especially at high doses)	Susceptibility testing is advised for the specific drug, not just the class of tetracyclines In vitro susceptibility seen in approximately 50% of *Staphylococcus* spp.^188^
	Minocycline hydrochloride^189^, ^190^ 5 mg/kg p.o. q12h	Human‐only tetracycline No published evidence of clinical efficacy in canine pyoderma except a temporary improvement in one dog^191^ Oral absorption better if administered on an empty stomach Gastrointestinal adverse effects (vomiting) more frequent than with doxycycline, especially at high doses^192^	Similar properties as doxycycline, yet some strains of *Staphylococcus*, including MRSP, that are resistant to doxycycline owing to *tet(K)*‐mediated resistance can be susceptible to minocycline
Potentiated sulfonamides	Trimethoprim‐sulfadiazine or trimethoprim‐sulfamethoxazole 15 mg/kg p.o. q12h (or 30 mg/kg p.o. q24 h)	Human/veterinary potentiated sulfonamides Dogs are more susceptible to adverse effects than other animals May be considered for dogs in otherwise good general health and if owners have been made aware of a greater risk for clinically significant adverse effects. Dose‐dependent adverse effects: keratoconjunctivitis sicca (occurs in about 15% of dogs, reversible when treatment is promptly stopped), folate deficiency, drug‐induced hypothyroidism Idiosyncratic adverse reactions/sulfonamide hypersensitivity: Overall risk greater than with other antimicrobials and true incidence unclear (0.25%–10% depending on study design^193^, ^194^; fever, blood dyscrasias, polyarthropathy, skin eruptions, acute hepatopathy (risk may be very low for ormetoprim‐sulfadimethoxine owing to a different elimination pathway in dogs), proteinuria A breed‐associated increased risk may exist for Dobermans yet evidence for this remains unclear Avoid in dogs with a known drug‐hypersensitivity	No approved susceptibility testing breakpoints available for isolates from animals; human breakpoints and testing standards may be applied using trimethoprim‐sulfamethoxazole May have good activity against some MRSA, MRSP and MRSC isolates Risk of emerging resistance or selection pressure from treatment is considered low
	Ormetoprim‐sulfadimethoxine 55 mg/kg p.o. loading dose, then 27.5 mg/kg p.o. q24h		

Abbreviations: CLSI, Clinical Laboratory Standards Institute; EUCAST, European Committee on Antimicrobial susceptibility testing; q12h, twice daily every 12 h; q24h, once daily every 24 h.

Changes to this drug group, compared to the previous pyoderma guidelines,^5^ are based on updates for laboratory testing standards (e.g. higher recommended doses for second‐generation fluoroquinolones),^179^ new data on the impact on the wider microbiome and extended discussions among the authors about clinical considerations. Although the potentiated sulfonamides may be effective against staphylococci and only have a minor impact on further drug resistance, the risk of adverse events in dogs is well documented. Their selection should be based on BC/AST results if available safer options have been excluded. Also, we no longer included ciprofloxacin, a human‐label generic oral fluoroquinolone, among the fluoroquinolones. Although ciprofloxacin has a similar in vitro activity against staphylococci as veterinary fluoroquinolones, in dogs its oral absorption is low and its bioavailability highly variable.^180^ Because approved testing standards and breakpoints for veterinary isolates are not currently available for ciprofloxacin and human breakpoints for this drug are inappropriate for veterinary isolates, meaningful susceptibility testing for ciprofloxacin is not possible and effective doses for dogs have not been established. The tetracyclines have been moved to the second‐choice group owing to a ≤ 50% chance of resistance when selected for empirical treatment.

### Reserved antimicrobial drugs

Drugs listed as ‘reserved’ should be limited to treating infections caused by multidrug‐resistant staphylococci, mainly MRSP, when no other first‐ or second‐choice options are appropriate (Table 16).

**TABLE 16:** Reserved antimicrobials drugs that may be considered for dogs with pyoderma owing to multidrug‐resistant, meticillin‐resistant staphylococcal pathogens (MRSP, MRSA, MRSC).

Reserved antimicrobial drugs: to be considered *only* when BC/AST results are available, for the management of multidrug‐resistant, meticillin‐resistant staphylococcal pathogens when first‐ and second‐choice drugs are not suitable, and all limitations have been discussed with owners			
Class	Drug & suggested dose	Comments & evidence base	Resistance considerations
Ansamycins	Rifampicin^a^ 5 mg/kg p.o. q12h or 10 mg/kg p.o. q24h (q12h dosing is preferred; do not exceed total daily dose of 10 mg/kg)	Idiosyncratic adverse reactions: hepatotoxicity; skin eruptions Metabolites may cause orange‐red discolouration of urine, faeces and saliva Recommended to monitor serum biochemistry before treatment and every 7–10 days Strong metabolic enzyme inducer that may diminish the effectiveness of other medications administered simultaneously (including barbiturates, ketoconazole, ciclosporin) No veterinary‐label formulations Restricted by regulatory authorities in some countries; consult local antimicrobial policies and restrictions	Highly active against *Staphylococcus* spp., including MRS Rare resistance has been identified in staphylococci from dogs^196^ In human medicine, typically used in combination with another antimicrobial agents to reduce the risk of treatment failure There are currently no data to support combination therapy over monotherapy in canine MRSP infections No approved susceptibility testing breakpoints available for isolates from animals; human breakpoints and testing standards may be applied
Aminoglycosides	Amikacin 15 mg/kg intravenously or intramuscularly or subcutaneously q24h	May be considered for dogs that are in otherwise good general health *and* if owners have been made aware of a greater risk for clinically‐significant adverse effects. Dose‐dependent adverse effects: nephrotoxicity (IRIS http://www.iris‐kidney.com/education/prevention.html)^197^, ^198^ Recommended to monitor serum biochemistry before treatment and every 7–10 days Check urine every 3–5 days for declining specific gravity (USG) and casts – early indicators for nephrotoxicity Higher doses have been mentioned, yet 15 mg/kg for amikacin (9–14 mg/kg for gentamicin) is considered sufficient for pyoderma (based on reaching 90% of therapeutic targets in a population of dogs). Higher doses up to 30 mg/kg once daily are only needed if the volume of distribution is greatly increased (e.g. third space syndrome)	These aminoglycosides can show good clinical efficacy with a lesser impact on further drug‐resistance Approved susceptibility testing standards and breakpoints available for staphylococci from dogs Active against susceptible strains of *Staphylococcus* spp. and more active against Gram‐negative bacilli than Gram‐positive cocci Amikacin may be more active than gentamicin against some isolates according to in vitro testing^199^
	Gentamicin 9–14 mg/kg i.v. or i.m. or s.c. q24h		
Amphenicols	Chloramphenicol 40–50 mg/kg p.o. q8h (consider the lower range of the dose for large dogs)	Many adverse effects are possible in animals Important: pet owners must be aware of the risks to their own health from accidental exposure: gastrointestinal, neurological, myelosuppression, particularly with longer use Owing to the risk (rare) of idiosyncratic, aplastic anaemia in humans, avoid direct contact, wear gloves Consider recheck every 2 weeks Approved for use in dogs in some countries, yet human‐label or restricted in others; consult local antimicrobial policies and restrictions Potent inhibitor of major cytochrome P450 enzymes^200^	Most *Staphylococcus* spp., including MRSP, will be resistant using the new CLSI testing standards and breakpoints^48^

Abbreviations: CLSI, Clinical Laboratory Standards Institute; MRSP, meticillin‐resistant *Staphylococcus pseudintermedius*; MRSA, meticillin‐resistant *S. aureus*; MRSC, meticillin‐resistant *S. coagulans* (formerly: *S. schleiferi)*; q8h, every 8 hours; q12h, twice daily every 12 h; q24 h, once daily every 24 h.

^a^ Rifampicin is the International Nonproprietary Name (INN) and the British Approved Name (BAN); rifampin is the US Adapted Name (USAN); rifamycin is an older name for the same drug, which is no longer used.

They are either not authorised for or in some countries banned from use in dogs because they are critically important for the treatment of serious infections in human medicine such as tuberculosis. Carbapenems are not included in our recommendations as they are not expected to be effective in MRSP infections and their use is considered inappropriate in canine pyoderma. Furthermore, all drugs in this group are associated with a risk of dose‐dependent predictable and/or idiosyncratic unpredictable serious adverse effects in dogs. Experience with and data on the use of these drugs and their clinical adverse effects are limited.

Microbiology laboratories may not routinely report susceptibilities for these agents. Consequently, extended susceptibility testing may have to be requested specifically for individual isolates. Given the importance of reserved antimicrobials for human medicine and to limit increasing drug resistance in the patient, consideration of the following five criteria is strongly recommended when evaluating the selection of reserved group antimicrobials.^195^

1. BC/AST results from a recent, representative sample indicate that the infecting MRS is susceptible to the agent under consideration and no other treatment options are appropriate.
2. The risks for adverse treatment effects have been evaluated after thorough clinical assessment of the dog's health status.
3. The prognosis for resolving the pyoderma is good and it is likely that resolution can be achieved within an acceptable duration of treatment.
4. Underlying primary causes have been identified, are treatable, and a plan is in place for long‐term management and prevention of recurrent secondary bacterial infections.
5. Owners have been made aware of the risks associated with treatment, of the need for compliance, and they are committed to the potentially life‐long follow‐up care needed.

If not *all* of these criteria are met, the potential harm from using reserved drugs for dog and human health will likely outweigh the short‐term benefit of a temporary resolution of infection, and other management options should be sought (see Section ‘What if recurrences keep happening?’).

### Strongly discouraged antimicrobial drugs

Drugs listed as ‘strongly discouraged’ are not licensed for use in animals (Table 17). They are of critical importance in human medicine for the treatment of serious infections involving multidrug‐resistant *S. aureus* and *Enterococcus* spp. Their use in all animals has been banned in the European Union since 2023.^201^ Although these drugs typically show good in vitro activity against veterinary isolates of *Staphylococcus* spp., their use in canine pyoderma is strongly discouraged. First, there is a risk of resistance emerging from treatment in the targeted pathogen but also in the commensal microbiota of the dog, with the potential for zoonotic transmission to in‐contact people. Secondly, veterinarians would rarely have sufficient experience with the use of these drugs to ensure safe and effective prescribing. If considering drugs from this group, we encourage consultation with or referral to a clinical expert (specialist) in a relevant area (infectious diseases, clinical pharmacology, dermatology) before prescribing.

**TABLE 17:** Antimicrobials for which the use in canine pyoderma is strongly discouraged.

Strongly discouraged antimicrobial drugs			
Class	Drug	Comments & evidence base	Resistance consideration
Oxazolidinone	Linezolid 10 mg/kg p.o. q12h	In people, administration for >2 weeks, without careful monitoring, is discouraged because of reversible myelosuppression. Myelosuppression has been described in adult dogs used in drug‐safety testing^202^ Restricted for use in dogs by regulatory authorities in some countries; consult local antimicrobial policies and restrictions	No approved susceptibility testing breakpoints available for isolates from animals; when testing canine isolates, human breakpoints and testing standards may be applied Linezolid‐resistant *Staphylococcus* spp. associated with ribosomal mutations are rare, yet plasmid‐mediated resistance mediated by the *cfr* gene can occur in isolates from animals
Glycopeptide	Vancomycin 15 mg/kg q6‐8h i.v. slow infusion (frequency of administration based on clinical monitoring) Constant‐rate infusion (CRI): loading dose of 3.5 mg/kg, followed by CRI of 1.5 mg/kg mixed in 5% dextrose in water	Must be administered to dogs by slow i.v. infusion. Oral vancomycin cannot be used as it is not absorbed by dogs Monitoring blood concentrations of vancomycin, preferably using the AUC (area under the curve), is recommended to optimise treatment and to avoid adverse effects and treatment failure.^203^ This type of monitoring is rarely available to veterinarians Risk of nephrotoxicity in dogs Restricted for use in animals by regulatory authorities in some countries; consult local antimicrobial policies and restrictions	No approved susceptibility testing breakpoints available for isolates from animals; when testing canine isolates, human breakpoints and testing standards may be applied Although there are no vancomycin‐resistant staphylococci reported from dogs to date, vancomycin‐resistant *Saureus* (VRSA), is a serious health concern in human medicine worldwide^204^

Abbreviations: q12h, twice daily; q6–8h, three or four times a day.

## 10 PREVENTING RECURRENCES OF PYODERMA

If underlying primary causes remain undiagnosed or untreated, all types of pyoderma can recur even following successful antimicrobial treatment.^205^ However, managing recurrences with repeated courses of systemic antimicrobials and historical strategies such as pulse‐ or low‐dose antimicrobial therapy is no longer considered safe or appropriate and must be avoided.^206^

### Undiagnosed underlying causes

In dogs that require systemic antimicrobial therapy more than once a year for recurrent pyoderma, the search for underlying primary causes must be intensified or repeated.

Based on findings from molecular studies, staphylococci isolated from pyoderma lesions are genetically indistinguishable from those at carriage sites.^41^ Thus, host factors such as altered immune, physical or chemical skin defence mechanisms must be present for pyoderma to occur, even if such factors cannot be easily diagnosed with available diagnostic tests. The concept of ‘idiopathic’ or ‘primary’ pyoderma is no longer tenable.

A thorough diagnostic review of potential primary underlying diseases should be repeated at least every 6–12 months, with the choice of tests guided by the skin lesions and clinical signs that remain after pyoderma has been resolved through successful treatment (see Section ‘Diagnostic approach and diagnostic tests’). Briefly, where the age of onset of recurrent pyoderma is young (<3 years), allergic skin disease is one of the most likely underlying causes. In older dogs, blood and urine analyses, including testing for endocrinopathies, imaging for neoplastic conditions, or skin histopathological evaluation, should be considered over time as some underlying conditions may only become detectable in more advanced stages. Ectoparasite prophylaxis, appropriate for regionally prevalent ectoparasites, should be in place before antimicrobial therapy is considered.

### Recurrent pyoderma in atopic dogs

In dogs with recurrent superficial pyoderma secondary to underlying allergic skin disease, appropriate medication or management strategies for their allergy should be prioritised over repeated treatment with antimicrobials.

Allergic, including atopic, skin diseases are suspected and diagnosed clinically if erythema and pruritus remain at predilection sites after ectoparasites and microbial infections have been resolved.^207^ The most appropriate drugs and management options for controlling allergic skin disease depend on the clinical signs and stage of disease in each case.^14^, ^208^, ^209^ If inflammatory signs predominate, especially if chronic‐hyperplastic changes are found, glucocorticoids and ciclosporin may be most suitable to control disease. In cases where pruritus is pronounced but inflammation is minimal, alternative anti‐pruritic drugs such as Janus kinase inhibitors (e.g. oclacitinib) or interleukin‐31 monoclonal antibodies (e.g. lokivetmab) also be adequate.

It is generally recommended to resolve a microbial infection first before starting immune‐modulatory treatment. This recommendation applies to all deep pyodermas where the risk of systemic spread may be high. In superficial and surface pyoderma, resolving the pyoderma first, before starting anti‐inflammatory medication, will allow the lowest necessary dose of the chosen anti‐allergy medication to be established. However, where dogs are already receiving treatment for their primary allergic disease or where signs of allergic disease are severe, starting, continuing or upgrading anti‐inflammatory treatment early may be of low risk for general health (provided doses are not immunosuppressive and progress can be monitored) and may actually be helpful by normalising the local skin microenvironment and thus improving resilience against infection.

Although seemingly counterintuitive, glucocorticoids (systemically or topically) or ciclosporin or other drugs to manage allergic pruritus (oclacitinib, lokivetmab) are best used to improve control of allergic skin disease and proactively prevent secondary bacterial infections. Evidence specific for which drugs to use to prevent recurrence of pyoderma in allergic dogs is lacking, yet studies suggest that prednisolone and oclacitinib may reduce the need for antimicrobial drugs in some atopic dogs.^210^, ^211^

### What if recurrences keep happening?

Proactive topical antimicrobial therapy with antiseptics may be effective in preventing relapses and can be maintained indefinitely.

Where pyoderma recurs despite continuing efforts to find and manage underlying causes, antimicrobial treatment will be required to resolve recurrent lesions and associated pruritus or pain. Topical antiseptics (e.g. 2%–4% chlorhexidine products) applied daily or 2–3 times weekly as needed may be necessary (see Section ‘Topical antimicrobial therapy’). Whether this approach also is effective in preventing recurrences of pyoderma has not been proven and future studies are urgently needed.

### Current or future alternative treatment options

Few alternatives to antimicrobial therapy have been investigated or are available.^212^ Autogenous *S. pseudintermedius* bacterins and S. *aureus* lysate were well‐tolerated and have been shown to reduce the need for antimicrobial treatment in dogs with recurrent pyoderma in several small studies.^213^, ^214^, ^215^, ^216^ Unfortunately, their availability is currently very limited in many countries due to licensing difficulties.

While bacteriophage therapy, skin probiotics and bacterial interference approaches may become available in the future, clinical studies in dogs with pyoderma have not been published to date.^217^, ^218^, ^219^

## 11 METICILLIN‐RESISTANT STAPHYLOCOCCAL PYODERMA

### General comments

General management concepts and treatment recommendations for surface, superficial, and deep pyoderma apply also to MRS pyoderma and additional detail can be found in the MRS‐specific guidelines, available from the World Association for Veterinary Dermatology (WAVD).^6^

Briefly, meticillin‐resistance in staphylococci is used as a marker for resistance to virtually all β‐lactam antibiotics, including first‐ and third‐generation cephalosporins and carbapenems. While there is no direct relationship between meticillin‐resistance and resistance to other antimicrobial classes, MRS have often acquired multiple additional resistances and many are resistant to most veterinary antimicrobials. This can make treatment selection for systemic therapy challenging, yet topically used agents, both antiseptics and topical antibiotics, have remained effective (Table S1). The main risk factors for MRSP infections in dogs include repeated courses of systemic antimicrobials, chronic disease associated with repeated visits to veterinary premises, hospitalisation and some surgical procedures.^220^, ^221^, ^222^ Pyoderma associated with MRS is clinically indistinguishable from pyoderma involving meticillin‐susceptible staphylococci and BC/AST is required for identification.^222^ The prognosis for MRS pyoderma is considered good, although one study has reported a longer duration to resolution of infection, which is likely to be associated with chronicity of infections.^91^ Because of the ability of multidrug‐resistant staphylococci to spread among dogs, between different host species, and to contaminate their environments, infection control measures and the potential for zoonotic transmission require consideration.^6^

If meticillin resistance is reported in coagulase‐negative staphylococci from skin, their clinical relevance may be doubtful and careful consideration of clinical signs and cytology is recommended before antimicrobial therapy is prescribed.

Coagulase‐negative staphylococci are widely distributed as commensals in animals and humans and are typically considered as nonpathogenic or barely pathogenic or as contaminants when reported from skin samples by BC/AST. Multidrug resistance, including meticillin resistance, is common in coagulase‐negative staphylococci yet will not trigger the same concerns for treatment and infection control measures as in coagulase‐positive staphylococci, except in immunocompromised individuals or where infection is associated with implants.^223^, ^224^

The relevance of coagulase‐negative staphylococci as pathogens in a skin infection needs to be confirmed through review of the clinical history, physical examination, cytology and review of sampling and culture method. A coagulase‐negative *Staphylococcus* isolated from intact primary lesions (e.g. a pustule or a furuncle ruptured in a sterile manner) or grown in pure culture from a tissue sample obtained through sterile biopsy technique is more likely to be a true pathogen than if isolated from a surface swab (Section ‘Diagnostic approach and diagnostic tests’). Accurate speciation of staphylococci by veterinary microbiology laboratories is therefore important.^223^, ^224^

### Treatment of MRS infection

Topical antimicrobial therapy as the sole antibacterial treatment modality is the treatment‐of‐choice for all cases of surface and superficial MRS pyoderma (SOR A).

If systemic therapy is needed for MRS pyoderma and if susceptibility is reported for non‐beta lactam, first‐choice or second‐choice antimicrobials, these should be prescribed.

If reserved‐group drugs (Table 16) are considered, recent laboratory test results need to be available, the larger clinical context needs to be acknowledged, and owners need to be fully aware of the more complex treatment requirements to avoid inappropriate use and disappointing treatment outcomes (see Section ‘Reserved antimicrobial drugs’).

Adjunctive topical antimicrobial therapy is recommended for every case of pyoderma that involves meticillin‐resistant staphylococci.

Antiseptics and topical antibiotics that have proven efficacy against staphylococci are expected to be effective against MRS also (see Section ‘Topical antimicrobial therapy’). Genes associated with higher MICs for chlorhexidine and some topical antibiotics have been identified in MRS from dogs, yet their clinical relevance remains unclear, and clinical treatment failures have not been reported with topical therapy.^155^

For systemic antimicrobial therapy, clindamycin may occasionally be appropriate from the first‐choice drug group if in vitro susceptibility is indicated by the report. Furthermore, dogs infected with ‘low‐level’ MRSP isolates may respond favourably to cefalexin or amoxicillin/clavulanic acid. These MRSP isolates typically display MICs of oxacillin between 0.5 and 2 mg/L, are cefalexin‐susceptible according to CLSI standards and breakpoints.^225^

If second choice‐drugs are needed and if susceptibility to fluoroquinolones is reported, it should be confirmed with the laboratory that the most recently revised CLSI breakpoints were used^179^ and the higher doses recommended in Table 16 used.

Within the reserved drugs group, most evidence and information is available for rifampicin in the treatment of MRSP pyoderma.^226^, ^227^, ^228^, ^229^, ^230^, ^231^, ^232^, ^233^, ^234^ Little is published on the use of amikacin for canine pyoderma; however, information on adverse drug effects is available based on experience in the management of other infections, especially those associated with the urinary tract.^197^, ^198^

Use of other drugs such as the oxazolidinones or glycopeptides in dogs is strongly discouraged and may be legally restricted in some countries. Their use can be considered unwarranted in cases of canine pyoderma where alternatives can be identified. Consultation with a dermatology, internal medicine, microbiology or infection control specialist is recommended to identify suitable alternative management plans.

### Infection control measures for the veterinary clinic or hospital

Dogs with MRS infection will contaminate their environment with multidrug‐resistant bacteria. Additionally, the majority (>60%) of dogs that have recovered from MRSP infection continued to carry MRSP on healthy skin and mucosae for many months.^229^ Dogs also will continue to contaminate clinical areas when visiting veterinary premises through natural shedding of hair and skin cells with adherent bacteria and from respiratory secretions. For MRSA, studies have shown that viable MRSA can be recovered from dry surfaces after 6 months and longer,^230^ and similar long environmental survival is expected for MRSP. Implementation of cleaning and disinfection protocols, including rigorous hand hygiene, should be instituted when a dog with MRS infection is visiting and guidelines for practice infection control measures are widely available, for example from the Ontario Animal Health Network.^231^ However, further measures such as screening and isolation strategies for dogs with MRSP pyoderma have not been evaluated in veterinary settings yet. In human medicine, it has been shown that the effectiveness and cost‐efficiency of screening and isolation measures varied with the local prevalence of MRSA and the intensity of the hospital setting.^232^ Given the wide range of regional MRSP prevalence rates across the world, recommendations on best screening and isolation practices will need to be made regionally, possibly in collaboration with local infection control advisory colleagues.

### Zoonotic potential

The risk of MRS transmission from dogs to humans is considered low for MRSP, potentially higher for the mostly human‐hospital‐associated pathogen MRSA and unknown for MRSC.^6^ Rare human infections with MRSP have been reported, and owners should be informed of the zoonotic potential and of the merits of rigorous hand hygiene and reducing exposure to infectious material.^233^, ^234^ However, the presence of MRSA suggests a probable human source of infection. Thus, it is advisable to talk to pet owners about the possibility of human‐to‐animal transmission of MRSA in the household when discussing management. Awareness about potential exposure to MDR bacteria from a dog's infection is particularly important for people with co‐morbidities or other risk factors for infection. For further concerns, owners should be advised to consult their own medical practitioner who can assess the risk of zoonotic transmission in light of the owner's medical history and that of other household members.

## CONCLUSIONS

With the skin being easily accessible for sampling and topical therapy, it offers unique opportunities for antimicrobial stewardship. Routine use of cytology, topical antimicrobial therapy and a focus on correcting underlying primary causes that lead to pyoderma will substantially reduce our reliance on systemic antimicrobials.

For cases that need systemic antimicrobial therapy, information presented in the document will help clinicians to choose and use antimicrobials responsibly according to available evidence and consensus. Adherence to the guidelines will increase the chances for successful treatment outcomes and reduce the risk of inappropriate selection pressure on bacterial pathogens and commensal flora. Knowledge gaps identified during the guideline process include the need for a better characterisation of some of the clinical presentations of canine pyoderma to improve diagnosis and, importantly, a standardisation of study design and outcome measures for clinical trials.

## AUTHOR CONTRIBUTIONS

**Anette Loeffler:** Conceptualization; methodology; data curation; project administration; writing – original draft; writing – review and editing. **Christine L. Cain:** Conceptualization; writing – original draft; methodology; writing – review and editing; data curation. **Lluís Ferrer:** Conceptualization; writing – original draft; methodology; writing – review and editing; data curation. **Koji Nishifuji:** Data curation; conceptualization; methodology; writing – original draft; writing – review and editing. **Katarina Varjonen:** Data curation; conceptualization; methodology; writing – original draft; writing – review and editing. **Mark G. Papich:** Data curation; conceptualization; methodology; writing – original draft; writing – review and editing. **Luca Guardabassi:** Conceptualization; methodology; writing – review and editing; data curation; writing – original draft. **Siân M. Frosini:** Conceptualization; methodology; writing – review and editing; data curation. **Emi N. Barker:** Conceptualization; writing – review and editing; methodology; data curation. **J. Scott Weese:** Data curation; methodology; writing – review and editing; conceptualization.

## FUNDING INFORMATION

Self‐funded.

## CONFLICT OF INTEREST STATEMENT

None of the authors have received any financial contribution for the guideline project.

## Supporting information


Table S1.

Table S2.

Table S3.

Table S4.

## Data Availability

Data sharing is not applicable to this article as no new data were created or analyzed in this study.
