# Supplementary material for: Antimicrobial use guidelines for canine pyoderma by the International Society for Companion Animal Infectious Diseases (ISCAID)
Source: Vet Dermatol. 2025 May 7;36(3):234–82. doi: 10.1111/vde.13342 (PMC12058580; doi:10.1111/vde.13342)
Supplement: Supplementary file 1 — Table S1. [file VDE-36-234-s004.docx]

| **Systematic review of TOPICAL antimicrobial therapy as sole or adjunctive therapy in the treatment of canine superficial pyoderma** | | | | | | | | |
| --- | --- | --- | --- | --- | --- | --- | --- | --- |
| **Reference** | **Study design** | **SORT Level of evidence (LoE)** | **Study characteristics** | | | | **Outcome / Clinical resolution** | **Microbiology information**  **(as provided)** |
|  |  |  | ***n* completed / enrolled** | **Antimicrobial therapy** | | **Duration of therapy** |  |  |
|  |  |  |  | **Topical** | **Systemic** |  |  |  |
| Bensignor et al., 2016 | RCT | 1 | 12/12 | Each applied to one half of the body twice daily (n = 12):  (A) Essential oils combination product: oils (manuka, lavandin, hemp seed); N-acetyl cysteine  (B) Water | Cefalexin 15 mg/kg p.o. twice daily (n = 12) | 28 days | Cure based on composite “total pyoderma score” (clinical assessment and cytological evaluation):  D14: (A) 50% (n = 6) versus (B) 8% (n = 1)  D21: (A) 83% (n = 10) versus (B) 50% (n = 6)  D28: (A) 100% (n = 12) versus (B) 83% (n = 10)  D35: (A) 100% (n = 12) versus (B) 100% (n = 12)  Total pyoderma score reduced in both treatment groups at D7, D14, D21 and D28 cf. D0 (*P <*0.05). Reduction in total pyoderma score was greater for (A) at D7 (*p =*0.0084), D14 (*p =*0.0038), and D21 (*p =*0.0340). | *Cytological evaluation*  Enrolment: intracellular cocci in all |
| Borio et al., 2015 | RCT | 1 | 41/53  LTF (n = 2), DFP (n = 1), concurrent disease (n = 3), clinical adverse event (n = 2), lack of efficacy (n = 2) | (A) 4% chlorhexidine digluconate shampoo (twice weekly) *and* 4% chlorhexidine digluconate spray once daily on non-shampoo days (n = 25/31) | (B) amoxicillin–clavulanate 25 mg/kg p.o. twice daily (n = 16/20; analysis for the group was reduced from 22 to 20 dogs after MRSP isolation) | 28 days | Composite “total pyoderma score” (clinical assessment and cytological evaluation): overall improvement in both groups.  No differences between treatment groups on D7 (*p =*0.96), D28 (*p =*0.51) and D56 (*p =*0.73).  5 dogs in each group had higher pyoderma scores on D56 cf. D28.  *Adverse effects*  (A) n = 2 erythema, scaling and pruritus attributed to treatment  (B) n = 2 withdrawn owing to “gastric disorder” (not clear if attributable to treatment) | *Cytological evaluation*  Enrolment: neutrophils with intracellular bacteria in all  *Culture*  Enrolment: *S. pseudintermedius* in 48 [n = 6 MRSP in topical group; n = 2 MRSP in systemic group (excluded from analysis; subsequently resolved with topical protocol)]; no growth 4/53; *Bacillus* spp. 1/53 [considered a contaminant] |
| Hsiao et al., 2021 | RCT | 1 | 28/29  Suspected skin reaction to spray (mild itch, scaling, and erythema; n = 1) | (A) 1.5% olanexidine gluconate spray once daily (n = 14/15)  (B) 3% chlorhexidine gluconate shampoo once weekly for 2 treatments (n = 14) | Systemic treatment not described, but not explicitly excluded during the study | 10 days | Clinical response (D0 🡪 D14):  Partial improvement in pyoderma score for both treatment groups [percentage reduction in pyoderma score]:  (A) reduced from 36.0 ± 15.8 (range 20–58) to 16.3 ± 12.1 (range 8–46) [54.8% ± 28.7%]  (B) reduced from 39.5 ±11.8 (range 20–58) to 22.3 ± 17.2 (range 6–40) [42.6% ± 33.2%]  No difference between treatment groups (*p =*0.73). | *Cytological evaluation*  Enrolment: intracellular cocci in all  *Culture*  Enrolment: *S. pseudintermedius* in all [MSSP (n = 14); MRSP (n = 15)] |
| Loeffler et al., 2011 | RCT | 1 | 20/22  Negative culture (n = 1); DFP (n = 1) | (A) 3% chlorhexidine gluconate shampoo twice weekly (n = 10/11)  (B) 2.5% benzoyl peroxide shampoo daily for 2 days, then twice weekly (n = 10/11) | None | 21 days | Clinical response:  Resolution or much improved (D0 🡪 D21): (A) 70% (n = 7) versus (B) 20% (n = 2) (*p <*0.05).  Papule/pustule scores:  (A) reduced (D0 🡪 D7; *p =*0.01) and (D0 🡪 D21; *p <*0.001)  (B) no change (D0 🡪 D21)  D 21 scores (A) < (B) (*p <*0.05)  1 dog treated with benzoyl peroxide had subjectively drier and finer coat at D22 | *Culture*  Enrolment: coagulase-positive staphylococci in 21 |
| de Jaham, 2003 | Nonrandomised clinical trial | 2 | 20/20 | (A) 10% ethyl lactate shampoo twice weekly (n = 10)  (B) No topical treatment (n = 10) | Cephalexin 25-30mg/kg p.o. q12h (n = 20) | 42 days | Clinical resolution  D 14: (A) 10% (n = 1) versus (B) 0%  D 28: (A) 80% (n = 8) versus (B) 30% (n = 3)  Severity index in (A) lower than group (B) on D14 and 28 (*P <*0.01).  Dogs in (A) had shorter average time to resolution than (B) (29.4 days versus 37.8 days; *p <*0.02). | Not described |
| Duangkaew, et al., 2017 | Prospective case series | 2 | 16/20  Progressive disease at D28 (n = 1) and D42 (n = 3) | Essential oils combination products:  Shampoo containing oils (manuka, oregano, rosemary, hemp seed) and extracts (propolis, honey, green apple) once weekly  *And* topical spray containing oils (Palmarosa, savory, sage, lavandin, lemon eucalyptus, hemp seed, tamaru) and extracts (ajowan, neem) applied topically 48h after the bath (n = 16/20) | None | 56 days | Of those that completed the trial (to D56): 94% (n = 15) had 75-100% improvement; one had no improvement | *Cytological evaluation*  Up to D56: no results shown  *Culture*  Enrolment: *Staphylococcus* spp. in all  D56: no results shown |
| Fadok and Irwin, 2019 | Prospective case series | 2 | 17/19  LTF (n = 1);  progressive disease at D14 (n = 1) | Bathed with sodium hypochlorite / salicylic acid shampoo (proprietary formulation) three times weekly (n = 17/19) | None | 28 days | Clinical severity score decreased from baseline (10.95 ± 2.92) to D14 (5.8 ± 2.7) and to D28 (3.65 ± 1.85) (both *p <*0.001) | *Cytological evaluation*  Enrolment: cocci present in all  D 14; 29% (n = 5) negative  D 28: 82% (n = 14) negative  *Culture*  Enrolment: *S. pseudintermedius* in all [MSSP (n = 2); MRSP (n = 17)] |
| Iyori et al., 2022 | RCT | 2 | 19/19 | Once weekly bathing in:  (A) carbonated water (pH 4.5) (n = 10)  (B) non-carbonated water (placebo) (n = 9) | None | 14 days | Clinical score  (A) 70% (n = 7) showed a >50% reduction in clinical scores (D0 🡪 D21)  (B) no change (D0 🡪 D21) | *Culture*  Enrolment: *S. pseudintermedius* in all (7/19 MRSP) |
| Marchegiani et al., 2023 | RCT | 2 | 20/22  DFP (n = 2) | (A) fluorescence biomodulation (Phovia) once weekly (n = 6/7)  (B) fluorescence biomodulation twice weekly (n = 6/7) | (C) cefadroxil 20 mg/kg p.o. twice daily (n = 8/8) | Until clinical resolution (up to 6 weeks) | Average time to clinical resolution (total disappearance of lesions):  (A) 2.40 ± 1.14 weeks  (B) 2.30 ± 0.67 weeks  (C) 3.75 ± 1.04 weeks  Difference in time to achieve clinical resolution (*p =*0.0291) between groups (but pairwise comparison not reported) | *Cytological evaluation*  Enrolment: “bacterial follicular involvement”  *Culture*  Enrolment: *S. pseudintermedius* in all [with other *Staphylococcus* spp. [n = 10]; *Enterococcus* spp. [n = 3]; *Streptococcus* spp. [n = 2]; *Proteus mirabilis* [n = 3]) |
| Murayama et al., 2010a | RCT | 2 † | 10/10 | Each applied to one half of the body twice weekly (n = 10):  (A) 2% chlorhexidine acetate surgical scrub  (B) 4% chlorhexidine gluconate shampoo | None | 7 days | Both products reported to reduce clinical signs (no statistical comparison D0 🡪 D7). None were considered to have clinical resolution. No difference between treatment used (*p =*0.56).  † Downgraded for lack of clinical and statistical detail | *Cytological evaluation*  Enrolment: consistent with pyoderma |
|  | Prospective case series | 2 | 10/10 | *Dogs from the above trial continued with:* | | | 1 dog had clinical resolution after 14 days of topical treatment alone.  Remainder had clinical resolution within 21 days | *Dogs from the above trial continued with:*  *Culture*  Performance at the discretion of clinicians and results not reported |
|  |  |  |  | 2% chlorhexidine acetate surgical scrub twice weekly | Dependent on susceptibility testing: cefalexin 20 mg/kg p.o. twice daily (n = 6) *or* minocycline 5 mg/kg (n = 3) p.o. twice daily *or* none (n = 1) | 7–21 days |  |  |
| Murayama et al., 2010b | RCT | 2 † | 10/10 | Each applied to one half of the body twice weekly (n = 10):  (A) 2% chlorhexidine acetate surgical scrub  (B) 2% chlorhexidine gluconate / 2% miconazole nitrate shampoo | None | 7 days | Both products reported to reduced clinical signs (no statistical comparison D0 🡪 D7).  Only one dog considered to have clinical resolution. No difference between treatment used (*p*-value not given).  † Downgraded for lack of clinical and statistical detail | *Culture*  Enrolment: *S. intermedius* group organisms in all (cefalexin-resistance noted in 5) |
| Murayama et al., 2011 | RCT | 2 | 27/27 | 2% chlorhexidine acetate surgical scrub applied to every 2 days at three different doses:  (A) 57  mL⁄m^2^ body surface area (n = 9)  (B) 29 mL⁄m^2^ body surface area (n = 9)  (C) 19 mL⁄m^2^ body surface area (n = 9) | None | 7 days | For each treatment group   - Majority of dogs considered to have a “good” response (improvement, with some lesions remaining after treatment) - Some but not all lesion scores improved from D0 🡪 D7 (*P <*0.05)   No difference in total outcome score or for each lesion score between groups (*p* > 0.05) | *Cytological evaluation*  Enrolment: neutrophils and intracellular cocci in all  *Culture*  Enrolment: performed but not reported |
| Seltzer et al., 2010 | RCT | 2 † | 21/26  Disease progression (n = 3);  DFP (n = 1); dermatophytosis (n = 1) | Sprayed once daily onto affected areas:  (A) 0.2% stannous fluoride (n = 11/12)  (B) vehicle/placebo (glycerin; zinc gluconate) (n = 10/14)  All dogs washed weekly with non-medicated shampoo | None | 28 days | Using both linear and non-linear models, clinical scores improved D0 🡪 D21 and D0 🡪 D42 (*p <*0.05). However, there was no difference in investigators clinical scores on D0 and D28 for either treatment group (*p*> 0.05).  All dogs in (A) and nearly all dogs in (B) (n = 11/12) had cytological evidence of pyoderma at D28.  Prescription of systemic antimicrobials:  (A) one on D14 (withdrawn from study); seven on D42 (study completion)  (B) three on D28 (withdrawn from study); seven on D42 (study completion)  † Downgraded for inconsistent detail for analysis | *Cytological evaluation*  Enrolment: evidence of bacterial skin infection in all  *Culture*  Enrolment: positive culture in all  (A) *S. intermedius-*group (SIG) (n = 11); other *Staphylococcus* spp. (n = 1); other bacteria (n = 3).  (B) SIG (n = 10); other *Staphylococcus* spp. (n = 2); other bacteria (n = 4; 2 with concurrent other staphylococci)  D 28:  (A; n = 11 cultured) SIG (n = 10); other *Staphylococcus* spp. (n = 2, one with concurrent other bacteria  (B; n = 12 cultured) SIG (n = 9); other *Staphylococcus* spp. (n = 2, one with concurrent other bacteria); other bacteria (n = 5) |
| Tochio et al., 2023 | Prospective case series | 2 | 10/10 | Erythritol (5%) / 0.1% l-ascorbyl-2-phosphate sprayed three times daily onto affected areas | None | 28 days | Clinical scores reduced D0 🡪 D28 (*p <*0.05). | *Cytological evaluation*  Enrolment: neutrophils with intracellular cocci in all  *Microbiota assessment*  Copy numbers of *S. pseudintermedius* and *S. schleiferi* decreased D0 🡪 D28 (*p <*0.05) |
| Aiemsaard et al., 2022 | Prospective case series ‡ | 3 | 8/8 | (A) 5% clove essential oil, 10% ethyl alcohol, 10% polyoxyethylene sorbitan monooleate, 75% isopropyl myristate mixture twice daily (8 h apart) (n = 4)  (B) 2% chlorhexidine gluconate spray twice daily (8h apart) (n = 4) | None | 15 days | Improvement in lesion score and staphylococcal count shown on D5, D10 and D15 (no difference between groups; *p*> 0.05), with negative culture achieved in all dogs by 10 days  Clinical difference (≥50% average reduction in clinical lesion score) achieved by D10 in (A) and by D5 in (B) (no difference between treatment groups; *p*> 0.05) | *Culture*  Enrolment: coagulase-positive staphylococci in all |
| Bryan et al., 2012 (Superficial & deep pyoderma) | Retrospective case series | 3 | 26/37  LTF (n = 5); DFP (n = 6) | Various (incompletely described) chlorhexidine shampoo, solution and spray, chlorhexidine and ketoconazole shampoo, wipes and flush, miconazole and chlorhexidine shampoo, mupirocin ointment, and benzoyl peroxide shampoo | None | Variable duration (details not given) | Dogs with MSSP (n = 2) had resolution of clinical signs.  Dogs with MRSP (17 had previously failed systemic antimicrobials): clinical signs resolved in 63% (n = 15), improved in 17% (n = 4), and did not improve in 21% (n = 5). | *Culture*  Enrolment: *S. pseudintermedius* in all [MSSP (n = 9); MRSP (n = 28)] |
| De Lucia et al., 2017  (Superficial & deep pyoderma) | Retrospective case series | 3 | 28/29  LTF (n = 1) | Various (incompletely described; n = 28/29)  Chlorhexidine formulation in 27(17 as sole topical agent; other agents included mupirocin cream (n = 7), fusidic acid cream (n = 2), sodium hypochlorite solution (n = 2), colloidal sulfur shampoo (n = 1), amikacin solution (n = 1), benzoyl peroxide shampoo (n = 1)) | Rifampicin 5 mg/kg p.o. twice daily for 7–70 days (aim: 7–14 days beyond clinical cure) (n = 28/29), one each with additional clindamycin or trimethoprim-sulfamethoxazole | Duration of topical therapy not given | Overall response was good in 71% (n = 20), moderate in (n = 6) and poor in (n = 2) | *Cytological evaluation*  Enrolment: neutrophils / macrophages, intracellular cocci  *Culture*  Enrolment: MRSP (n = 20); MRSA (n = 1); other coagulase-positive meticillin-resistant staphylococci (n = 11) |
| Murayama et al., 2010a | Prospective case series | 3 | 8/8 | 2% chlorhexidine acetate surgical scrub every 2 days | After 14 days, if deemed clinically appropriate  Dependent on susceptibility testing | 14–56 days | Clinical resolution   - 5 after 14 days of topical therapy alone - 2 after 35 days topical therapy and 21 days of minocycline (5mg/kg orally twice daily)   1 did not have complete response to 8 weeks of topical treatment and 6 weeks of minocycline (5 mg/kg p.o. twice daily); but “improved” following a switch to topical treatment and ciprofloxacin (10 mg/kg p.o. twice daily) for 6 weeks | *Cytological evaluation*  Enrolment: consistent with pyoderma  *Culture*  Enrolment: cefalexin-resistant *S. intermedius* group isolated |
| Narang et al., 2015  (“Bacterial dermatitis”) | Prospective case series | 3 | 6/6 | Polyherbal (citronella, lemongrass, eucalyptus) with sesame oil base once daily | None | 7–21 days | 100% (n = 6/6) resolution by D21 | Not described |

RCT, randomised controlled trial; LTF, lost to follow up; DFP, deviated from protocol

†Study level of evidence (LoE) amended for reasons given under “Outcome”

| **Level of evidence (LoE)** | | **Definition for treatment studies** |
| --- | --- | --- |
| 1 | Good quality, patient-orientated | “High quality” randomised controlled trial (RCT) OR meta-analysis of consistent RCTs with ≥10 dogs per group. |
| 2 | Limited quality patient-orientated | “Low quality” RCT downgraded either owing to <10 dogs per group, lack of separate assessment of groups, lack of specific clinical interpretation OR prospective case series (cohort study) containing ≥10 dogs |
| 3 | Other evidence | Prospective case series containing <10 dogs OR a retrospective case series (any size) |

# References

Aiemsaard J, Aiyaranoi K, Thongkham E, Borlace GN, Senaphan K. In Vivo efficacy of clove essential oil spray formulation on canine superficial pyoderma. [Songklanakarin J Sci Technol. 2022;44:308](https://openurl.ebsco.com/results?sid=ebsco:ocu:record&bquery=IS+0125-3395+AND+VI+44+AND+IP+2+AND+DT+2022)–15

Bensignor E, Fabriès L, Bailleux L. A split-body, randomized, blinded study to evaluate the efficacy of a topical spray composed of essential oils and essential fatty acids from plant extracts with antimicrobial properties. Vet Dermatol. 2016;27:464–e123.

Borio S, Colombo S, La Rosa G, De Lucia M, Damborg P, Guardabassi L. Effectiveness of a combined (4% chlorhexidine digluconate shampoo and solution) protocol in MRS and non–MRS canine superficial pyoderma: a randomized, blinded, antibiotic-controlled study. Vet Dermatol. 2015;26:339–44.

de Jaham C. Effects of an ethyl lactate shampoo in conjunction with a systemic antibiotic in the treatment of canine superficial bacterial pyoderma in an open-label, nonplacebo-controlled study. Vet Ther. 2003;4:94–100.

Duangkaew L, Larsuprom L, Lekcharoensuk C, Chen C. Effect of a mixture of essential oils and a plant–based extract for the management of localized superficial pyoderma in dogs: an open-label clinical trial. Thai J Vet Med. 2017;47:513–22.

Fadok VA, Irwin K. Sodium hypochlorite/salicylic acid shampoo for treatment of canine staphylococcal pyoderma. J Am Anim Hosp Assoc. 2019;55:117–123.

Hsiao YH, Imanishi I, Iyori K. Efficacy of olanexidine gluconate in canine superficial pyoderma: a randomised, single–blinded controlled trial. Vet Dermatol. 2021;32:664-e174.

Iyori K, Tamagawa S, Ide K, Nishifuji K. Clinical efficacy of artificially carbonated water bathing on superficial bacterial folliculitis in dogs. Vet Dermatol. 2022;33:36-e12.

Loeffler A, Cobb MA, Bond R. Comparison of a chlorhexidine and a benzoyl peroxide shampoo as sole treatment in canine superficial pyoderma. Vet Rec. 2011;169:249.

Marchegiani A, Spaterna A, Fruganti A, Cerquetella M. Exploring fluorescent light energy as management option for canine superficial bacterial folliculitis. Front Vet Sci. 2023 Jun 2;10:1155105.

Murayama N, Nagata M, Terada Y, Shibata S, Fukata T. Efficacy of a surgical scrub including 2% chlorhexidine acetate for canine superficial pyoderma. Vet Dermatol. 2010a;21:586–92.

Murayama N, Nagata M, Terada Y, Shibata S, Fukata T. Comparison of two formulations of chlorhexidine for treating canine superficial pyoderma. Vet Rec. 2010b;167:532–3.

Murayama N, Terada Y, Okuaki M, Nagata M. Dose assessment of 2% chlorhexidine acetate for canine superficial pyoderma. Vet Dermatol. 2011;22:449–53.

Seltzer JD, Flynn–Lurie AK, Marsella R, Brennan MM. Investigation of the clinical efficacy of 0.2% topical stannous fluoride for the treatment of canine superficial pyoderma: a prospective, randomized, double-blinded, placebo-controlled trial. Vet Dermatol. 2010;21:249–58.

Tochio T, Kawano K, Iyori K, Makida R, Kadota Y, Fujii T, et al. Topical erythritol combined with L-ascorbyl-2-phosphate inhibits staphylococcal growth and alleviates staphylococcal overgrowth in skin lesions of canine superficial pyoderma. Pol J Vet Sci. 2023;26:647–55.

Bryan J, Frank LA, Rohrbach BW, Burgette LJ, Cain CL, Bemis DA. Treatment outcome of dogs with methicillin-resistant and methicillin-susceptible Staphylococcus pseudintermedius pyoderma. Vet Dermatol. 2012;23:361–8,e65.

De Lucia M, Bardagi M, Fabbri E, Ferreira D, Ferrer L, Scarampella F, et al. Rifampicin treatment of canine pyoderma due to multidrug-resistant methicillin-resistant staphylococci: a retrospective study of 32 cases. Vet Dermatol. 2017;28:171–e36.

Narang A, Arora N, Krishan G, Rajora VS, Singh JL. Therapeutic studies on canine dermatoses with special reference to polyherbal formulation. Vet Practit. 2015;16:140–3.
