# Supplementary material for: Antimicrobial use guidelines for canine pyoderma by the International Society for Companion Animal Infectious Diseases (ISCAID)
Source: Vet Dermatol. 2025 May 7;36(3):234–82. doi: 10.1111/vde.13342 (PMC12058580; doi:10.1111/vde.13342)
Supplement: Supplementary file 2 — Table S2. [file VDE-36-234-s002.docx]

| **Systematic review of SYSTEMIC antimicrobial therapy in the treatment of canine superficial pyoderma** | | | | | | | | | | |
| --- | --- | --- | --- | --- | --- | --- | --- | --- | --- | --- |
| **Citation** | **Study design** | **SORT level of evidence** | **Study characteristics** | | | | **Clinical outcomes** | | | **Microbiological assessment (results as reported at enrolment unless stated otherwise)** |
|  |  |  | **n completed / enrolled** | **Antimicrobial therapy (systemic)** | **Adjunctive therapy (topical)** | **Treatment duration** | **≤3 weeks** | **>3 weeks** | |  |
| Littlewood et al., 1999 | RCT | 1 | 56 / 59 | 1. Clindamycin 5.5 mg/kg p.o. twice daily (n = 29) 2. Amoxicillin-clavulanate 12.5 mg/kg p.o. twice daily (n = 27) | Localised 2% chlorhexidine gluconate / 2% miconazole nitrate shampoo only if *Malassezia* identified | 21–42 days | At 21 days:   1. 17/29 (59%) had resolution; 4 had persistent disease attributed to clindamycin-resistance (withdrawn) 2. 8/27 (30%) had resolution | At 42 days (of those not resolved at 21 days):   1. 5 received treatment; 4 had resolution; 1 had treatment failure attributed to clindamycin-resistance 2. 14 received treatment; 10 had resolution; 4 had persistent disease | | *Cytological evaluation*  *Culture*  Enrolment (n = 56): 46/56 coagulase-positive *Staphylococcus* spp. cultured; 4/56 coagulase-negative *Staphylococcus* spp.; 17/56 mixed culture; no growth in 5  At D21 (n = 56): all isolates were sensitive to amoxicillin-clavulanate and 5 were resistant to clindamycin  At D42: data not given |
|  |  |  |  |  |  |  | 1. 3 dogs with persistence of disease withdrawn after 21 days were subsequently reported to have resolution (2 after therapy for underlying disease) 2. 3 dogs with persistence of disease were withdrawn at 21 days; 2 further lost to follow-up at 42 days were subsequently reported to have resolution (after therapy for underlying disease)   Treatment (A) was more effective than (B) for most lesion scores | | |  |
| Lloyd et al., 1997 | RCT | 1 | 47 / 68 | 1. Amoxicillin-clavulanate 12.5 mg/kg p.o. twice daily (n = 20) 2. Amoxicillin-clavulanate 25 mg/kg p.o. twice daily (n = 27) | Not permitted during study | 2–12 weeks | At 14 days: 13/47 had resolution  At 21 days: further 7/47 had resolution | Between 4 and 5 weeks: further 15/47 had resolution  Between 6 and 8 weeks: further 8/47 had resolution  4/47 had no response to treatment | | *Culture*  All: *Staphylococcus* spp. |
|  |  |  |  |  |  |  | Median duration of treatment required to resolution: 4 weeks  No difference between the treatment groups (*p* = 0.57) | | |  |
| Messinger & Beale, 1993 | RCT | 1 | 43 / 45 | 1. Trimethoprim-sulfadiazine [target dose 30 mg/kg; actual dose mean 31.6 mg/kg, range 30.0‑34.3 mg/kg] p.o. once daily (n = 13/14) 2. Trimethoprim-sulfadiazine [target dose 30 mg/kg; actual dose mean 34.5 mg/kg, range 30.2–40.7 mg/kg] p.o. twice daily (n = 14/14)   (C) Ormetoprim-sulphadimethoxine; D 1 [target dose 55 mg/kg; actual mean dose 68.9 mg/kg, range 55.0‑80.0 mg/kg]; D2 onwards [target dose 27.5 mg/kg; actual mean dose 29.4 mg/kg, range 27.5‑40.0 mg/kg] p.o. once daily (n = 16/17) | Topical antimicrobials continued if commenced prior to study | 3 weeks; extended to 6 weeks if not resolved at 3 weeks | All dogs enrolled showed clinical improvement at 3 weeks.  At 3 weeks:   1. 5/13 (38%) had resolution 2. 8/14 (57%) had resolution   (C) 12/16 (75%) had resolution | At 6 weeks:   1. further 5/13 (38%) had resolution; 2. further 3/14 (21%) had resolution 3. further 4/16 (25%) had resolution | | *Culture*  (n = 45): 44 with *S. [pseud]intermedius*, 2 with resistance to trimethoprim-sulfamethoxazole (excluded); 1 with non-enteric Gram-negative rod |
|  |  |  |  |  |  |  | The 6 dogs without resolution at 6 weeks [3 in (A) and 3 in (B)] were given cefalexin (22 mg/kg p.o. once daily for 3 weeks) and achieved resolution.  No difference on overall cure between groups (*p* = 0.092) | | |  |
| Six et al., 2008 | RCT | 1 | 129 / 320 | 1. Cefovecin 8 mg/kg SC every 14 days (n = 62) 2. Cefadroxil 22 mg/kg p.o.   twice daily (n = 67) | Not permitted during study | 14–28 days | At 14 days:   1. 45/62 treatment success 2. 44/67 treatment success | At 28 days:  (A) 12/62 treatment success  (B) 16/67 treatment success | | *Culture*  (n = 235/320 overall study): *S.[pseud]intermedius* 117/235; coagulase-negative staphylococci 38/235; others 109/235 |
|  |  |  |  |  |  |  | Treatment (A) was considered noninferior to treatment (B) | | |  |
| Stegemann et al., 2007 | RCT | 1 | 152 / 152 | 1. Cefovecin 8 mg/kg SC every 14 days (n = 107) 2. Amoxicillin-clavulanate 12.5 mg/kg p.o. twice daily (n = 45) | Not permitted during study | 14 days; extended by 14-day intervals as required (up to 56 days) | Clinical success (all signs mild to absent) achieved:  At 14 days in 39% (n = 42/107) in (A) and in 44% (n = 20/45) in (B) | Clinical success (all signs mild to absent) achieved:  At 21 days in a further 43% (n = 46/107) in (A) and 24% (n = 11/45) in (B)  At 42-56 days in a further 17% (n = 18/107) (A) and 22% (n = 10/45) (B)  Treatment failure in 1% (n = 1/107) in (A) and 9% (n = 4/45) in (B) | | *Cytological evaluation*  All: degenerate neutrophils and phagocytosis  *Culture*  All: one or more pathogen cultured  Of the 425 isolates from the overall study the most prevalent were *S. [pseud]intermedius* (52.5%; n = 223)*,* *E. coli* (10.4%; n = 44)*,* beta-haemolytic *Streptococcus* spp. (7.5%; n = 32), *Enterobacter* spp. (7.1% n = 30) |
|  |  |  |  |  |  |  | Treatment (A) was considered noninferior to treatment (B) | | |  |
| Toma et al., 2008 | RCT | 1 | 40 / 40 | 1. Cefalexin [target dose 30‑40 mg/kg; actual mean dose 33.3±4.34 mg/kg] p.o. once daily (n = 20) 2. Cefalexin [target dose 15‑30 mg/kg; actual mean dose 35.7±5.48 mg/kg] p.o. twice daily (n = 20) | Not mentioned | 4–8 weeks (until up to 14 days after clinical resolution) | At D14:   1. 4/20 cured 2. 6/20 cured | At D28:   1. further 11/20 cured 2. further 10/20 cured   At D42:   1. remaining 5 cured 2. remaining 4 cured | | *Cytological evaluation*  (n = 40): intracellular bacteria in neutrophils in all  *Culture*  (n = 40): positive in all; *S.[pseud]intermedius* in 29 (13 Group A, 16 Group B); other bacteria isolated, alone or in combination, included other staphylococci (n = 6) and various gram-positive and gram-negative organisms (n = 14) |
|  |  |  |  |  |  |  | Response to treatment considered equivalent.  †Downgraded for lack of randomisation | | |  |
| Angarano & MacDonald, 1989 | Prospective case series | 2 | 19 / 19 | (A) Cefadroxil, 22 mg/kg p.o. twice daily | Not permitted during study | 21–30 days | At 3 weeks:  Good to excellent response in all (n = 19) | - | | *Cytological evaluation* (no results presented)  *Culture*  (n = 13/30 overall study): *Staphylococcus* spp. in all |
| Barnabas et al., 2017 | RCT | 2 † | 40 / 40 | 1. Cefpodoxime 5 mg/kg p.o. once daily (n = 20) 2. Cefalexin 30 mg/kg p.o. twice daily (n = 20) | Not permitted during study | 28 days | “Good response” seen for both treatment groups. Time to recovery: 14-22 days for (A) and 20-28 days for (B) – reported as not significant (*p*-value not given).    † Downgraded for lack of detail of clinical assessment after treatment (no numbers presented) | | | *Culture*  All: *S. [pseud]intermedius* |
| Bensignor et al., 2016 | Prospective case series ‡ | 2 | 12 / 12 | 1. Cefalexin 15 mg/kg p.o. twice daily | Essential oil-based vs. placebo spray, each to one half of the body twice daily | 4 weeks | At D21:  6/12 (placebo) and 10/12 (essential oil) treated halves cured | At D28:  10/12 (placebo) and 12/12 (essential oil) treated halves cured | | *Cytological evaluation*  All: Intracellular cocci |
| Bettenay et al., 1998 | Prospective case series | 2 | 30 / 30 | 1. Doxycycline 5  mg/kg load, then 2.5 mg/kg at 12 and 24 h, then 2.5 mg/kg p.o. once daily | Chlorhexidine 2% / miconazole shampoo 2% (n = 2) | 21–42 days | At D21:  16/30 (53%) clinical remission; 12/30 (40%) partial response | At D42 (of those with partial response at D21): 2/12 (17%) clinical remission | | *Cytological evaluation*  All: Intracellular cocci  *Culture*  (n = 65/65 overall study): 8/65 no growth [3 from deep pyoderma], 50/65 *S. [pseud]intermedius* |
|  |  |  |  |  |  |  | 12/30 (40%) poor response after 3-6 weeks of treatment | | |  |
| Bloom & Rosser, 2001 | Prospective case series | 2 | 21 / 21 | (A) Clindamycin 11 mg/kg p.o. once daily | Not mentioned | 28–56 days (continued 14 days past clinical resolution) | At D14:  3/21 excellent response | At D28: further 12/21 had excellent response | | *Cytological evaluation*  All: Intracellular cocci  *Culture*  (n = 21/21): *Staphylococcus* spp. in 20; no growth in 1 |
|  |  |  |  |  |  |  | Owing to poor response: treatment discontinued at 14 days in 2, at 28 days in 3, and at 42 days in 1. | | |  |
| Borio et al., 2015 | Prospective case series ‡ | 2 | 16 / 22 | (A) Amoxicillin-clavulanate 25 mg/kg p.o. twice daily | Not mentioned | 28 days | - | At D28: all (n = 16) had resolution | | *Cytological evaluation*  All: Neutrophils, intracellular bacteria  *Culture*  *S. pseudintermedius* in 19, of which 2 were MRSP (excluded); *Bacillus* spp. in 1 (suspected contaminant); no growth in 2 |
| De Jaham, 2003 | Prospective case series ‡ | 2 | 20 / 20 | 1. Cefalexin [target dose 25‑30 mg/kg; actual mean dose 26.9 mg/kg) p.o. twice daily | 10% ethyl lactate shampoo twice weekly (n = 10) | 14–42 days | At D14:  1/20 (with shampoo adjunctive) resolved | At D28: further 10/20 resolved (7 with shampoo)  At D42: further 9/20 resolved (2 with shampoo) | | *Cytological evaluation*  All: Neutrophils, phagocytosed cocci |
|  |  |  |  |  |  |  | All dogs achieved remission by D42. Mean duration of treatment required: 33.6 days.  Dogs with adjunctive shampoo had shorter average time to resolution than without (29.4 days versus 37.8 days; *p* < 0.02). | | |  |
| Frank & Kunkle, 1993 | RCT | 2 † | 33 | (A) Cefadroxil 25.7 mg/kg p.o. twice daily (n = unknown)  (B) Generic cefalexin 24.9 mg/kg p.o. twice daily (n = unknown)  (C) Proprietary cefalexin 26.2 mg/kg p.o. twice daily (n = unknown) | Antibacterial shampoos continued if commenced prior to study | 3–6 weeks | At 3 weeks: 26/33 had clinical resolution | At 5 weeks: further 6/33 had clinical resolution | | *Culture*  Enrolment: *S. [pseud]intermedius* in all  Follow-up: MRSP in dog with failure to respond |
|  |  |  |  |  |  |  | One dog failed to resolve owing to need for flea control.  Mean duration available for overall study (superficial/deep pyoderma combined):  (A) 3.9±1.7 weeks;  (B) 3.7±1.6 weeks;  (C) 3.9±1.8 weeks  † Downgraded for lack of detail on numbers of treatment groups | | |  |
| Harvey et al., 1993 | Nonrandomised controlled trial | 2 | 30 / 33 | 1. Lincomycin hydrochloride 22 mg/kg p.o. twice daily (n = 14) 2. Clindamycin hydrochloride 11 mg/kg p.o. once daily (n = 16)   (three dogs dropped out, unknown which groups) | Not permitted during study | 21–42 days | At 21 days:   1. 10/14 (71%) had a good response 2. 13/16 (81%) had a good response | At 42 days (of those not resolved at 21 days):   1. further 3/14 had a good response 2. further 2/16 had a good response | | *Cytological evaluation*  Enrolment: cocci and neutrophils in all  *Culture*  Enrolment: *S. [pseud]intermedius* in all  At resolution (n = 25/33): culture of pustule (n = 1/25) isolated *S. [pseud]intermedius* |
|  |  |  |  |  |  |  | One dog in group (A) lost to follow-up at 6 weeks. One dog in group (B) failed to respond to 6 weeks of treatment, but subsequently responded to treatment with cefalexin.  No difference between groups (*p* not presented). | | |  |
| Harvey*,* 1996 | Prospective case series | 2 | 30 / 30 | (A) Tylosin 20 mg/kg p.o. twice daily | Not mentioned | 3–5 weeks | At 3 weeks: 22/30 (73%) had resolution | At 5 weeks: further 2/30 (7%) had resolution  6/30 (20%) had a poor response to 5 weeks of treatment | | *Culture*  (n =  30): 25 coagulase-positive staphylococci; 5 coagulase-negative staphylococci |
| Holm et al., 2004 | Prospective case series | 2 | 40 / 40* | 1. Cefalexin 20 mg/kg p.o. twice daily | Lesions cleaned and treated with astringents (solution of aluminium acetate or boric acid) once or twice daily | 3 weeks | All (n = 40) “improved” within 7–10 days of treatment | - | | *Culture*  (overall study; n = 27/44): *S. [pseud]intermedius* in 25; no growth in 2 |
| Paradis et al., 1990 | Prospective case series | 2 | 26 / 26 | (A) Enrofloxacin [target dose 2.5 mg/kg] p.o. twice daily | Not permitted during study | 1–14 weeks (overall study, n = 30; to 7‑10 days beyond resolution) | Response excellent (i.e. complete resolution) in 25/26 (93%), poor (i.e. static to progressive) in 1. The latter subsequently responded to a 3-week course of amoxicillin-clavulanate.  Mean duration of treatment to clinical resolution: 3.1 weeks (overall study; n = 30) | | | *Culture*  (n = 7/30 overall study): *S. [pseud]intermedius* in 5; 1 each of *S. epidermis* and *Proteus* sp. |
| Paradis et al., 2001 | Prospective case series | 2 | 62 / 62 | (A) Marbofloxacin [target dose 2.75 mg/kg; actual dose mean 2.73 mg/kg, range 1.8‑5.7 mg/kg] p.o. once daily | Not permitted during study | 3 weeks, extended by 7 days if considered necessary | At 3 weeks: 33/62 had resolved and 25 had partial response  4 failed treatment at or before 3 weeks. | At 4 weeks (of those with a partial response at 3 weeks): a further 20 had resolved and 5 had partial response. | | *Culture*  (n = 47/72 overall study): 69 bacterial isolates: 40 coagulase-positive staphylococci (no growth from 5 samples) |
|  |  |  |  |  |  |  | Actual treatment duration 8–28 days | | |  |
| Reddy et al., 2014a | Prospective case series | 2 | 8 / 10 | (A) Cefpodoxime-clavulanate [target dose 5 mg/kg] p.o. once daily | 2.5% benzoyl peroxide shampoo twice weekly advised | 3–4 weeks (to 7 days beyond resolution) | At D14: 7/8 had resolution  At D21: 1/8 had resolution | - | | Not reported |
| Reddy et al., 2014b | Prospective case series | 2 | 8 / 9 | (A) Enrofloxacin [target dose 5 mg/kg] once daily | 2.5% benzoyl peroxide shampoo twice weekly advised | 3–4 weeks (to 7 days beyond resolution) | At D14: 5/8 had resolution  At D21: 3/8 had resolution | - | | Not reported |
| Restrepo et al., 2010 | Prospective case series | 2 | 12 / 12 | (A) Pradofloxacin [target dose 3 mg/kg; overall study actual mean dose 3.7 mg/kg, range 3.0‑4.5 mg/kg] p.o. once daily | Not permitted during study | 28 days | - | At D28: 2/12 had complete resolution, 9 had an excellent response (>75% resolved), and 1 had a good response (50–75% resolved) | | *Cytological evaluation*  Enrolment (n = 12): cocci in all, rods in one  Day 28 (n = 12): cocci in 8, reduction in numbers seen in most  *Culture*  Enrolment (n = 12): *S.[pseud]intermedius* in 9; one each of meticillin-resistant coagulase-negative *Staphylococcus* spp., *Pseudomonas aeruginosa,* no growth |
| Scott et al., 1993 | Prospective case series | 2 | 15 / 15 | (A) Ormetoprim-sulfadimethoxine; D1 [target dose 55 mg/kg]; D2 onwards [target dose 27.5 mg/kg] p.o. once daily | Not permitted during study | 17–64 days | At 17–21 days: 5/15 complete resolution; treatment discontinued in 2 at D14 owing to poor response | At 22–56 days: complete resolution in the remaining | | *Cytological evaluation*  All: suppurative to pyogranulomatous inflammation with intracellular  *Culture*  All: *S. [pseud]intermedius* susceptible to the respective study antimicrobial drug  Follow-up swabs:  2 dogs not responding to tylosin 10 mg/kg twice daily had isolates resistant to tylosin after 14 days  2 dogs not responding to tylosin 20m/mg twice daily had isolates still susceptible to tylosin after 14 days |
|  |  |  |  |  |  |  | Mean time to resolution: 26 days | | |  |
| Scott et al., 1994 | Prospective case series | 2 | 12 / 12 | (A) Tylosin [target dose 20 mg/kg] p.o. twice daily | Not permitted during study | 14–24 days | At 17–21 days: 8/12 had complete resolution  Treatment discontinued in 1 at day 14 owing to poor response | At 23–24 days: remaining 3 had complete resolution | |  |
|  |  |  |  |  |  |  | Treatment period average: 21 days | | |  |
| Scott et al., 1996 | Prospective case series | 2 | 19 / 19 | (A) Tylosin [targe dose 10 mg/kg] p.o. twice daily | No dogs received topical antimicrobials during study | 14–35 days | At 17-21 days: 11/19 had complete resolution;  Treatment discontinued in 3 at 14-17 days owing to poor response | At 22–35 days: remaining 5 had complete resolution | |  |
|  |  |  |  |  |  |  | Treatment period average: 23 days | | |  |
| Sentürk et al., 2005 | Prospective case series | 2 | 18 / 18 | (A) Rifampicin [target dose 5 mg/kg] p.o. twice daily | Not mentioned | 10 days | At 10 days: all (n = 18/18) had clinical resolution | - | | *Culture*  (n = 18): 12 coagulase-positive staphylococci; 6 *S. epidermidis*) |
| Thirunavukkarasu et al., 2011 | Prospective case series | 2 | 10 / 10 | (A) Ceftriaxone-tazobactam [target dose 20 mg/kg] i.v. twice daily | Not mentioned | 7 days | 10/10 “recovered uneventfully” | - | | *Cytological evaluation*  Rods, cocci, neutrophils, and macrophages  *Culture*  *Staphylococcus* spp. and others |
| Varshney & Devi, 2020 | Prospective case series | 2 | 40 / 40 | 1. Linezolid [target dose 10 mg/kg] p.o. twice daily | 4% chlorhexidine gluconate shampoo once weekly; 5% povidone iodine spray once daily | 4 weeks | Most showed improvement within 2 weeks | At 4 weeks: complete recovery in all | | *Cytological evaluation*  Enrolment (n = 40): cocci in all  *Culture*  Enrolment (n = 40): MLSB (macrolide-lincosamide-streptogramin B)-resistant *S. aureus*, susceptible to linezolid in all  Day 28 (n = 40): no growth in all |
| Bassett et al., 2004 | Prospective case series | 3 | 3 / 3 | 1. Cefalexin 22 mg/kg p.o. twice daily (n = 2) 2. Amoxicillin-clavulanate 14 mg/kg twice daily (n = 1) | Not mentioned | 4–6 weeks | - | (A) 2/2 clinical resolution after 4 weeks  (B) 1/1 clinical resolution after 6 weeks | | *Culture*  *S. pseudintermedius* in 1*; S. aureus* and *Streptococcus* spp. in 1; no growth in 1 |
| De Lucia et al., 2017 | Retrospective case series | 3 | 11 / 11 | 1. Rifampicin [actual dose median 5 mg/kg; range 4‑10 mg/kg] p.o. twice daily | Various: 2-4% chlorhexidine formulations (n = 10), two with 2% miconazole; colloidal sulfur shampoo (n = 1) | 1–6 weeks | With 1–3 weeks of treatment:  3 had good and 1 had moderate responses | With 4–6 weeks of treatment:  4 had good and 3 had moderate responses | | *Cytological evaluation*  All: Intracellular cocci  *Culture*  MDR meticillin-resistant coagulase-positive staphylococci sensitive to rifampicin in all |
|  |  |  |  |  |  |  | Median duration of treatment: 5 weeks. | | |  |
| Harbour et al., 2022 | Retrospective case series | 3 | 37 / 37 | 1. Rifampicin [inclusion dose ≤6  mg/kg; actual dose mean 4.48±0.9 mg/kg] p.o. once daily | Various (overall study; n = 47/51): 2-4% chlorhexidine-based products, bleach (1:32 dilution), mupirocin/ gentamicin, other antiseptic shampoos | 3–52 days (whole study) | Treatment (variable duration) achieved complete resolution (based on cytological and physical examination) in 28/37 (75.7%). | | | *Cytological evaluation*  Enrolment / follow-up: described as reviewed, but data not given  *Culture*  Enrolment: MDR meticillin-resistant staphylococci susceptible to rifampicin in all  Follow-up (overall study, n = 4/51): all cultured rifampicin-resistant staphylococci, 2 following relapse and 2 following poor response to treatment |
| Marchegiani et al., 2023 | Prospective case series * | 3 | 8 / 8 | (A) Cefadroxil 20 mg/kg p.o. twice daily | Not mentioned | Up to 6 weeks (to 14 days beyond resolution) | - | Average time to clinical resolution 3.8±1.0 weeks  * Included only for the systemic antimicrobial treatment component of the study | | *Cytological evaluation*  All: “Confirmed bacterial involvement"  *Culture*  All: *S. pseudintermedius,* some in mixed culture; all susceptible to cefadroxil |
| Murayama et al., 2010 | Prospective case series | 3 | 12 / 12 | Following 1 week (n = 9) or 2 (n = 3) of topical therapy alone | | 1–6 weeks of systemic treatment | All were improving prior to starting systemic treatment | | | *Cytological evaluation*  All: consistent with pyoderma  *Culture*  At least 3 with cefalexin-resistant *S. intermedius* group organisms |
|  |  |  |  | 1. Cefalexin [20 mg/kg] p.o. twice daily (n = 6) 2. Minocycline [target dose 5 mg/kg] p.o. twice daily (n = 3, another 3 in RCT) | 2% chlorhexidine acetate twice weekly (n = 9) or every 2 days (n = 3) |  | 1. 1/6 resolved after 1 week; 5/6 resolved after 3 weeks   (B) 5/6 resolved in 3 weeks | (B) 1/6 not resolved at 6 weeks; subsequently improved on 8 weeks ciprofloxacin. | |  |
| Scott et al., 2006 | Prospective case series | 3 | 6 / 7 | (A) Orbifloxacin [target dose 2.5 mg/kg] p.o. once daily | Not permitted during study | 21–40 days (to 7 days beyond resolution) | At 21 days: 4/6 had complete resolution | At 23–33 days: remaining 2 had complete resolution | | *Cytological evaluation*  All: suppurative to pyogranulomatous inflammation with intracellular  *Culture*  All: *S. [pseud]intermedius* susceptible to the antimicrobial drug |
|  |  |  |  |  |  |  | Treatment periods: 21–40 days (average 29 days) | | |  |
| Sofou et al., 2022 | Prospective case series | 3 | 6 / 6 | Unknown [28 dogs in overall study]: amoxicillin-clavulanate [target dose 20‑25 mg/kg; overall study actual dose 16.9‑27.6 mg/kg] p.o. twice daily (n = 19) or clindamycin [target dose 11 mg/kg; overall study actual dose 10‑12.3 mg/kg] p.o. once daily or enrofloxacin [10 mg/kg] p.o. once daily | Not permitted during study | 27–47 days (until up to 7 days after clinical resolution) | 6/6 “resolution confirmed clinically and cytologically”  Mean duration of treatment: 33 days | | | Not reported |
|  | Retrospective case series | 3 | 6 / 6 |  | Unknown [overall study n = 17/19] 2% chlorhexidine gluconate / 2% miconazole nitrate shampoo 2-3 times weekly | 21–36 days (until up to 7 days after clinical resolution) | 6/6 “resolution confirmed clinically and cytologically”  Mean duration of treatment: 27 days | | |  |
| Sunilchandra et al., 2016 | Retrospective case series | 3 | 60 / 60 | 1. Azithromycin 5‑10 mg/kg p.o. once daily (n = 30) 2. Levofloxacin 10 mg/kg p.o. once daily (n = 30) | Not mentioned | 21–30 days | At D14:   1. 8/30 complete resolution 2. 25/30 complete resolution   At D14–D21:   1. 17/30 complete resolution 2. 5/30 complete resolution | | At D21–D30: further 5 had complete resolution in (A) (owner reported) | Not reported |
|  |  |  |  |  |  |  | Different duration of therapy to complete cure between treatment groups (*p*< 0.05): 16.9±0.5 days for (A) and 13.1±0.5 days for (B) | | |  |

RCT, randomised controlled trial

* Number of cases reviewed as compatible with superficial pyoderma based on description in text

†Study level of evidence (LoE) amended for reasons given under “Outcome”

| **Level of Evidence (LoE)** | | **Definition for treatment studies** |
| --- | --- | --- |
| 1 | Good quality, patient-orientated | “High quality” randomised controlled trial (RCT) OR meta-analysis of consistent RCTs with ≥10 dogs per group. |
| 2 | Limited quality patient-orientated | “Low quality” RCT downgraded either owing to <10 dogs per group, lack of separate assessment of groups, lack of specific clinical interpretation OR prospective case series (cohort study) containing ≥10 dogs |
| 3 | Other evidence | Prospective case series containing <10 dogs OR a retrospective case series (any size) |

# References

Angarano DW, MacDonald JM. Efficacy of cefadroxil in the treatment of bacterial dermatitis in dogs. J Am Vet Med Assoc. 1989;194:57–9.

Barnabas CR, Nagarajan B, Bhavani MS. Comparative efficacy, superiority and clinical benefits of cefopodoxime proxetil over cephalexin in canine pyoderma – A clinical study of 60 dogs. Intas Polivet 2017;18:120–3.

Bassett RJ, Burton GG, Robson DC. Antibiotic responsive ulcerative dermatoses in German Shepherd Dogs with mucocutaneous pyoderma. Aust Vet J. 2004;82:485–9.

Bensignor E, Fabriès L, Bailleux L. A split-body, randomized, blinded study to evaluate the efficacy of a topical spray composed of essential oils and essential fatty acids from plant extracts with antimicrobial properties. Vet Dermatol. 2016;27:464-e123.

Bettenay SV, Mueller RS, Dell’Osa D. Doxycycline hydrochloride in the treatment of canine pyoderma. Aust Vet Practit. 1998;28:14.

Bloom PB, Rosser EJ. Efficacy of once-daily clindamycin hydrochloride in the treatment of superficial bacterial pyoderma in dogs. J Am Anim Hosp Assoc. 2001;37:537–42.

Borio S, Colombo S, La Rosa G, De Lucia M, Damborg P, Guardabassi L. Effectiveness of a combined (4% chlorhexidine digluconate shampoo and solution) protocol in MRS and non-MRS canine superficial pyoderma: a randomized, blinded, antibiotic-controlled study. Vet Dermatol. 2015;26:339–44.

de Jaham C. Effects of an ethyl lactate shampoo in conjunction with a systemic antibiotic in the treatment of canine superficial bacterial pyoderma in an open-label, nonplacebo-controlled study. Vet Ther. 2003;4:94–100.

De Lucia M, Bardagi M, Fabbri E, Ferreira D, Ferrer L, Scarampella F, et al. Rifampicin treatment of canine pyoderma due to multidrug-resistant meticillin-resistant staphylococci: a retrospective study of 32 cases. Vet Dermatol. 2017;28:171-e36.

Frank LA, Kunkle GA. Comparison of the efficacy of cefadroxil and generic and proprietary cephalexin in the treatment of pyoderma in dogs. J Am Vet Med Assoc. 1993;203:530–3.

Harbour L, Schick A, Mount R, White A. Rifampicin treatment of canine multidrug-resistant meticillin-resistant staphylococcal pyoderma: A retrospective study of 51 cases. Vet Dermatol. 2022;33:384–91.

Harvey RG, Noble WC, Ferguson EA. A comparison of lincomycin hydrochloride and clindamycin hydrochloride in the treatment of superficial pyoderma in dogs. Vet Rec. 1993;132:351–3.

Harvey RG. Tylosin in the treatment of canine superficial pyoderma. Vet Rec. 1996;139:185–7.

Holm BR, Rest JR, Seewald W. A prospective study of the clinical findings, treatment and histopathology of 44 cases of pyotraumatic dermatitis. Vet Dermatol. 2004;15:369–76.

Littlewood JD, Lakhani KH, Paterson S, Wood JL, Chanter N. Clindamycin hydrochloride and clavulanate-amoxycillin in the treatment of canine superficial pyoderma. Vet Rec. 1999;144:662–5.

Lloyd DH, Carlotti DN, Koch HJ, Van den Broek AH. Treatment of canine pyoderma with co-amoxyclav: a comparison of two dose rates. Vet Rec. 1997;141:439–41.

Marchegiani A, Spaterna A, Fruganti A, Cerquetella M. Exploring fluorescent light energy as management option for canine superficial bacterial folliculitis. Front Vet Sci. 2023;10:1155105.

Messinger LM, Beale KM. A blinded comparison of the efficacy of daily and twice daily trimethoprim-sulfadiazine and daily sulfadimethoxine-ormetoprim therapy in the treatment of canine pyoderma. Vet Dermatol 1993;4:13–8.

Murayama N, Nagata M, Terada Y, Shibata S, Fukata T. Efficacy of a surgical scrub including 2% chlorhexidine acetate for canine superficial pyoderma. Vet Dermatol. 2010;21:586–92.

Paradis M, Lemay S, Scott DW, Miller WH, Wellington J, Panich R. Efficacy of enrofloxacin in the treatment of canine bacterial pyoderma. Vet Dermatol. 1990;1:123–7.

Paradis M, Abbey L, Baker B, Coyne M, Hannigan M, Joffe D, et al. Evaluation of the clinical efficacy of marbofloxacin (Zeniquin) tablets for the treatment of canine pyoderma: an open clinical trial. Vet Dermatol. 2001;12:163–9.

Reddy BS, Nalini Kumari K, Vaikunta Rao V, Rayulu VC. Efficacy of cefpodoxime with clavulanic Acid in the treatment of recurrent pyoderma in dogs. ISRN Vet Sci. 2014:467010.

Reddy BS, Kumari KN, Rao VV, Rayulu VC, Sivajothi S. Efficacy of enrofloxacin in the treatment of recurrent pyoderma in dogs. J Adv Vet Res. 2014;4:108–12.

Restrepo C, Ihrke PJ, White SD, Spiegel IB, Affolter VK. Evaluation of the clinical efficacy of pradofloxacin tablets for the treatment of canine pyoderma. J Am Anim Hosp Assoc. 2010;46:301–11.

Scott DW, Miller WH, Wellington JR. The combination of ormetoprim and sulfadimethoxine in the treatment of pyoderma due to *Staphylococcus intermedius* infection in dogs. Canine Practice 1993:29–33.

Scott DW, Miller WH Jr, Cayatte SM, Bagladi MS. Efficacy of tylosin tablets for the treatment of pyoderma due to *Staphylococcus intermedius* infection in dogs. Can Vet J. 1994;35:617–21.

Scott DW, Miller WH Jr, Rothstein SE, Bagladi MS. Further studies on the efficacy of tylosin tablets for the treatment of pyoderma due to *Staphylococcus intermedius* infection in dogs. Can Vet J. 1996;37:617-8.

Scott DW, Peters J, Miller WH Jr. Efficacy of orbifloxacin tablets for the treatment of superficial and deep pyoderma due to *Staphylococcus intermedius* infection in dogs. Can Vet J. 2006;47:999–1002.

Sentürk S, Özel E, Sen A. Clinical efficacy of rifampicin for treatment of canine pyoderma. Acta Vet/ Brno. 2005;74:117–22.

Six R, Cherni J, Chesebrough R, Cleaver D, Lindeman CJ, Papp G, et al. Efficacy and safety of cefovecin in treating bacterial folliculitis, abscesses, or infected wounds in dogs. J Am Vet Med Assoc. 2008;233:433–9.

Sofou EI, Aleksandrova S, Badulescu E, Chatzis M, Saridomichelakis M. Efficacy of antimicrobial treatment in dogs with atopic dermatitis: an observational study. Vet Sci. 2022;9:385.

Stegemann MR, Coati N, Passmore CA, Sherington J. Clinical efficacy and safety of cefovecin in the treatment of canine pyoderma and wound infections. J Small Anim Pract. 2007;48:378–86.

Sunilchandra U, Santhosh PS, Vijay Kumar M, Ravindra BG, Halmandge S. Comparative efficacy of azithromycin and levofloxacin in pyoderma of dogs. World J Pharmaceutical Res. 2016;5:1338–41.

Thirunavukkarasu PS, Srinivasan SR, Senthilkumar K, Chandrasekar M, Nambi AP, Prathaban S. Effect of ceftriaxone and tazobactam in the management of canine pyoderma. Intas Polivet. 2011;12:76–7.

Toma S, Colombo S, Cornegliani L, Persico P,Galzerano M, Gianino MM, et al. Efficacy and tolerability of once daily cephalexin in canine superficial pyoderma: an open controlled study. J Small Anim Pract 2008;49:384–91.

Varshney JP, Devi S. Refractory canine pyoderma due to macrolide-lincosamide-streptogramin B (MLSB) resistant Staphylococcus aureus and its management. Vet Practit. 2020;21:233–7.
