# Supplementary material for: Antimicrobial use guidelines for canine pyoderma by the International Society for Companion Animal Infectious Diseases (ISCAID)
Source: Vet Dermatol. 2025 May 7;36(3):234–82. doi: 10.1111/vde.13342 (PMC12058580; doi:10.1111/vde.13342)
Supplement: Supplementary file 3 — Table S3. [file VDE-36-234-s001.docx]

| **Systematic review of SYSTEMIC antimicrobial therapy in the treatment of canine deep pyoderma** | | | | | | | | | |
| --- | --- | --- | --- | --- | --- | --- | --- | --- | --- |
| **Citation** | **Study design** | **SORT level of evidence** | **Study characteristics** | | | | **Outcome / clinical resolution** | | **Microbiological information**  **(results as reported at enrolment unless stated otherwise)** |
|  |  |  | **n completed / enrolled** | **Antimicrobial therapy** | **Adjunctive antimicrobial therapy** | **Duration of treatment** | **≤4 weeks** | **>4 weeks** |  |
| Lloyd et al., 1997 | RCT | 1 | 21 / 68 | (A) Amoxicillin–clavulanate 12.5 mg/kg p.o. twice daily (n = 11/20)  (B) Amoxicillin–clavulanate 25 mg/kg p.o. twice daily (n = 10/15) | Not permitted during study | 2–12 weeks | Clinical resolution in 6/21 at 2–4 weeks | Clinical resolution in additional 11/21 at 5–12 weeks | *Culture*  *Staphylococcus* spp. susceptible to amoxicillin–clavulanate in all |
|  |  |  |  |  |  |  | No difference in response rate between treatment groups (Fisher exact test *p =*0.3108) or in mean duration of treatment in dogs with clinical resolution | |  |
| Mueller & Stephan, 2007 | RCT | 1 | 107 / 135 | (A) Pradofloxacin 3 mg/kg p.o. once daily (n = 56/66)  (B) Amoxicillin–clavulanate 12.5 mg/kg p.o. twice daily (n = 51/69) | Not permitted during study | 3–9 weeks | Overall response:  (A) 48/56 (86%) complete; 4/56 (7%) partial; 4/56 (7%) poor  (B) 37/51 (73%) complete; 3/51 (6%) partial; 5/51 (10%) poor  Median time to resolution:  (A) 49 days  (B) 35 days  No difference between treatment used (*p >*0.05) | | *Culture*  Dogs: n = 135: no growth in 5 (excluded); remaining dogs were positive for one or more organism; predominance of *Staphylococcus* spp. isolated (115 isolates from 61 dogs); 251/254 bacterial isolates susceptible to pradofloxacin |
| Stegemann et al., 2007 | RCT | 1 | 85 / 85 | (A) Cefovecin 8 mg/kg s.c. every 14 days (n = 63)  (B) Amoxicillin–clavulanate 12.5 mg/kg p.o. twice daily (n = 22) | Not permitted during study | 14 days; extended by 14-day intervals as required (up to 56 days) | Clinical success (all signs mild to absent) achieved:  At 14 days in 40% (n = 25) of treatment (A) and 36% (n = 8) in (B)  At 28 days in 19% (n = 12) in (A) and 23% in (B) | Clinical success (all signs mild to absent) achieved:  At 42 days in 22% (n = 14) in (A) and 14% (n = 3) in (B)  At 56 days in 10% (n = 6) in (A) and 14% (n = 3) in (B)  Treatment failure in 10% (n = 6) in (A) and 14% (n = 3) in (B) | *Cytological evaluation*  Degenerate neutrophils and phagocytosis, red blood cells, and macrophages in all  *Culture*  One or more pathogen cultured from all  Of the 425 isolates from the overall study the most prevalent were *S. [pseud]intermedius* (52.5%; n = 223)*,* *E. coli* (10.4%; n = 44)*,* beta–haemolytic *Streptococcus* spp. (7.5%; n = 32), *Enterobacter* spp. (7.1% n = 30) |
|  |  |  |  |  |  |  | Treatment (A) was considered noninferior to treatment (B) | |  |
| Angarano & MacDonald, 1989 | Prospective case series | 2 | 11 / 11 | (A) Cefadroxil 22 mg/kg p.o. twice daily | Not permitted during study | 21–30 days | At 3 weeks: 10/11 (91%) good to excellent response | – | *Culture*  Overall study: n = 13/30: *Staphylococcus* spp. |
| Bettenay et al., 1998 | Prospective case series | 2 | 35 / 35 | 1. Doxycycline 5  mg/kg load, then 2.5 mg/kg at 12 and 24 hours, then 2.5 mg/kg p.o. once daily | Chlorhexidine 2% / miconazole shampoo 2% (n = 3) | 3–6 weeks | At 3 weeks: 5/35 (14%) clinical remission, 18/35 (51%) partial response | At 6 weeks (of those 18 dogs with partial response at 3 weeks): 2/18 (11%) clinical remission | *Cytological evaluation*  Cocci and inflammatory cells in all  *Culture*  Overall study: n =  3/65 no growth; 50/65 *S. [pseud]intermedius*; 7/65 other bacteria |
|  |  |  |  |  |  |  | 12/35 (34%) poor response after 3–6 weeks of therapy | |  |
| Chirayath & Rathish, 2015 | Prospective case Series | 2 | 12 / 12 | (A) Clindamycin 11 mg/kg p.o. once daily | Not mentioned | ≥21 days; extended by 7 days until clinical resolution | At 4 weeks: 11/12 (92%) clinical resolution (in 7/12 by 3 weeks) | | 1/12 failed to resolve after 2 months of treatment |
| Frank & Kunkle, 1993 | RCT | 2† | 11 / 11 | (A) Cefadroxil 25.7 mg/kg p.o. twice daily (n = unknown)  (B) Generic cefalexin 24.9 mg/kg p.o. twice daily (n = unknown)  (C) Proprietary cefalexin 26.2 mg/kg p.o. twice daily (n = unknown) | Antibacterial shampoos continued if commenced prior to study | 3–9 weeks | Complete resolution (n = 10/11): within 3 weeks in 5; within 3–9 weeks in 5 (3 needed 9 weeks)  Mean duration available for overall study (superficial/deep pyoderma combined):  (A) 3.9±1.7 weeks;  (B) 3.7±1.6 weeks;  (C) 3.9±1.8 weeks  † Downgraded for lack of detail on numbers of treatment groups | | *Culture*  *S. [pseud]intermedius* in all; one in mixed culture  Follow–up: MRSP in dog with failure to respond |
| Gutierrez et al., 2020 | Prospective case series | 2 | 55 / 55 | (A) Enrofloxacin 10 mg/kg p.o. once daily | 0.5% enrofloxacin gel three times daily | Up to 16 days | Complete resolution in all within 16 days | | Not reported |
| Marchegiani et al., 2021  (Deep pyoderma) | Prospective case series * | 2 | 34 / 35 | 1. Cefalexin 20 mg/kg p.o. twice daily (n = 17/18) 2. Cefalexin 20 mg/kg p.o. twice daily (n = 17/17) | Only for group (B): fluorescence biomodulation applied twice weekly | ≥3 weeks and until 7 days after clinical resolution | Clinical resolution (taken from figure in manuscript):  (A) 11% (n = 2/18) at 4 weeks  (B) 47% (n = 8/17) at 4 weeks | Clinical resolution:  (A) 35% (n = 6/17) resolution at 8 weeks  (B) 88% (n = 15/17) resolution at 8 weeks | *Cytological evaluation*  Intracellular cocci in all; presence of rods was an exclusion criterion  *Culture*  Predominantly *Staphylococcus* spp. (35 isolates), all susceptible to cefalexin; other bacterial species detected infrequently (numbers not given) |
|  |  |  |  |  |  |  | Median time to resolution:  (A) 12 weeks  (B) 5 weeks  * Included only for the systemic antimicrobial treatment component of the study | |  |
| Marchegiani *et al.,* 2022  (Interdigital furunculosis) | Prospective case series | 2 | 12 / 12 | 1. Cefalexin 20 mg/kg p.o.   twice daily (n = 12) | Fluorescence biomodulation applied once weekly | ≥3 weeks and until 7 days after clinical resolution | Clinical resolution:  100%; with a median time to resolution of 4.5 weeks | | *Cytological evaluation*  Intracellular cocci in all  *Culture*  Coagulase-positive staphylococci in all |
| Paradis *et al.,* 2001 | Prospective case series | 2 | 10 / 10 | (A) Marbofloxacin [target dose 2.5–5.5  mg/kg; overall study actual dose mean 2.73  mg/kg, range 1.8–5.7  mg/kg] p.o. once daily | Not permitted during study | 3–4 weeks | At D28  9 had clinical resolution  1 had partial response | | Not reported |
| Scott et al., 1998 | Prospective case series | 2 | 20 / 20 | (A) Clindamycin 11 mg/kg p.o. once daily | Not mentioned | 21–91 days | At D21–D28: 6/20 (30%) complete resolution | At D31–D91: 14/20 complete resolution | *Culture*  All: *S. [pseud]intermedius* susceptible to the respective study antimicrobial drug at enrolment |
|  |  |  |  |  |  |  | Treatment periods: 21–91 days (average 45 days) | |  |
| Scott et al., 2006 | Prospective case series | 2 | 16 / 16 | (A) Orbifloxacin 2.5 mg/kg p.o. once daily | No dogs received topical antimicrobials during study | 25–150 days | At D25: 1/16 complete resolution | At D31–D150: 15/16 complete resolution |  |
|  |  |  |  |  |  |  | Treatment period average 72 days | |  |
| Špruček et al., 2007 | Prospective case series | 2 | 29 / 29 | 1. Cefalexin 30 mg/kg p.o.   once daily  (In addition: inactivated Parapoxvirus–based immunomodulator given i.m. once weekly to 10 dogs) | Not mentioned | 9–11 weeks | – | | Complete resolution in 76% (n = 22/29) at 9–11 weeks (mean 9.6 weeks)  No difference from use of concurrent immunomodulator after 2 months (*p =*0.2451) |
| Cain & Mauldin, 2015 | Retrospective case series  (acute “post–grooming” furunculosis) | 3 | 16 / 20 | (A) Enrofloxacin [actual dose range 10–12.2  mg/kg] or marbofloxacin [actual dose range 3.7–6.3  mg/kg] p.o. once daily (n = 16)  (B) Cefalexin or cefpodoxime, dose / frequency not given (n = 4) | Twice weekly ethyl lactate shampoo and 4% chlorhexidine shampoo (n = 2)  1% silver sulfadiazine cream twice daily to affected area (n = 1) | 5–42 days (median 30 days) | Clinical resolution in 100% (n = 16/16); treatment response unknown for 4  (where response was documented) systemic signs often resolved within 48 h | | *Cytological evaluation*  Overall study: n = 9/22: 9/9 inflammatory cells, 2/9 erythrocytes, 3/9 cocci, 3/9 rods  *Culture*  Overall study: n = 14/22: 10/14 *Pseudomonas aeruginosa*; 1 each of *S. hominis*, *Klebsiella oxytoca*, and *Burkholderia cepacian;* no growth in 1 |
| De Lucia et al., 2017 | Retrospective case series | 3 | 20 / 21 | 1. Rifampicin [actual dose median 5 mg/kg; range 4–10 mg/kg] p.o. twice daily   One each with additional clindamycin or TMPS | Various combinations of chlorhexidine, mupirocin, fusidic acid, sodium hypochlorite, benzoyl peroxide, and amikacin (n = 17) | 2–10 weeks | 11/20 (55%) good (complete resolution) and 2/20 (10%) moderate (partial resolution) response with 2–4 weeks treatment duration | 5/20 (25%) good response with 5–10 weeks treatment duration  Treatment discontinued in 2/20 (10%) following poor (static or progressive) response after 6 and 8 weeks treatment duration | *Cytological evaluation*  All: intracellular cocci  *Culture*  MDR meticillin–resistant staphylococci sensitive to rifampicin in all: 12 *S. pseudintermedius*, 1 *S. aureus*, 7 as “coagulase–positive” staphylococci |
| Harbour et al., 2022 | Retrospective case series | 3 | 14 / 14 | 1. Rifampicin [target dose 2.3–6  mg/kg; actual dose mean 4.48±0.9  mg/kg] p.o. once daily | 47/51 (overall study): chlorhexidine–products, bleach (1:32), mupirocin/ gentamicin, benzoyl peroxide, acetic acid, boric acid, plant extracts | 3–52 days (whole study) | Complete resolution (based on cytological and physical examination) in 11/14 (78.6%) | | *Cytological evaluation*  Enrolment / follow-up: described as reviewed, but data not given  *Culture*  Enrolment: MDR meticillin–resistant staphylococci susceptible to rifampicin in all  Follow–up (overall study, n = 4/51): all cultured rifampicin–resistant staphylococci, 2 following relapse and 2 following poor response to treatment |
| Holm et al., 2004 | Prospective case series | 3 | 4 / 4* | 1. Cefalexin 20 mg/kg p.o. twice daily | Lesions cleaned and treated with astringents (solution of aluminium acetate or boric acid) once or twice daily | 3 weeks | 4/4 (100%) “improved” within 7–10d | – | *Culture*  Overall study: n = 27/44  25 *S. [pseud]intermedius* including one MDR organism; no growth in 2 |
| Krick & Scott, 1989  (German Shepherd deep pyoderma) | Retrospective case series | 3 | 17 / 17 | Initial treatment course; various: amoxicillin–clavulanate (n = 2); erythromycin (n = 2); lincomycin (n = 2); oxacillin (n = 11) | Chlorhexidine (unknown concentration) soak once daily (n = 9) | 3–6 weeks | Clinical resolution in 14/17 (82%) at 3–6 weeks; of which 7/14 relapsed 10–20 days after stopping treatment | | *Cytological evaluation*  Intracellular cocci in all  *Culture*  Dogs: n = 11/17: 8 *Staphylococcus* spp.; no growth in 3 |
| Marchegiani et al., 2019  (Interdigital pyoderma) | Prospective case series * | 3† | 32 / 36 | 1. Various [n = 14/17]: cefalexin 20 mg/kg p.o. twice daily (n = 12); amoxicillin–clavulanate 20 mg/kg p.o. twice daily (n = 1); enrofloxacin 5 mg/kg p.o. once daily (n = 1) 2. Various [n = 18/19]: cefalexin 20 mg/kg p.o. twice daily (n = 17); enrofloxacin 5 mg/kg p.o. once daily (n = 1) | Only for group (B): fluorescence biomodulation applied twice weekly (n = 18/19) | ≥3 weeks and until 7 days after clinical resolution | Clinical resolution:  (A) 14% (n = 2/14) at 4 weeks  (B) 63% (n = 12/19) at 4 weeks | Clinical resolution:  (A) 26.5% (no n given) at 6 weeks  (B) 84.6% (no n given) at 6 weeks  †Downgraded for lack of detail on numbers | *Cytological evaluation*  Intracellular cocci in all  *Culture*  *Staphylococcus* spp. in all; 3 with MDR organisms (excluded) |
|  |  |  |  |  |  |  | Median time to resolution:  (A) 10 weeks  (B) 3.5 weeks  * Included only for the systemic antimicrobial treatment component of the study | |  |
| Paradis *et al.,* 1990 | Prospective case series | 3 | 4 / 4 | (A) Enrofloxacin 2.5 mg/kg p.o. twice daily | Not permitted during study | 1–12 weeks | Clinical resolution in 3/4, poor response in 1/4.  Lack of data on separate pyoderma groups. Duration of therapy across in those that achieved clinical remission (n = 28/30) was 1–12 weeks (mean 3.1 weeks) with deep pyoderma requiring a longer duration of treatment. | | *Culture*  Overall study: n = 7/30: 5 *S. [pseud]intermedius*; 1 each of *S. epidermidis* and *Proteus* sp*.* |
| Parida *et al.,* 2013 | Non–randomised controlled trial | 3 † | 24 / 24 | (A) Amoxicillin–clavulanate acid 20 mg/kg i.v. twice daily (n = 12)  (B) Ceftriaxone–tazobactam 20 mg/kg i.v. twice daily (n = 12) | Not mentioned | 10 days | At D10:  (A) “significant recovery” in 10/12 (83.3%)  (B) resolution in all  Mean time to resolution or “significant recovery”:  (A) 6.67 ± 0.17 days; excluding the 2 dogs that required therapy >10 days  (B) 4.56 ± 0.24 days | – | “Microbial examination”, no results presented |
|  |  |  |  |  |  |  | † Downgraded for lack of clinical detail on diagnosis and outcome assessment | |  |
| Reddy *et al.,* 2014a | Prospective case series | 3 | 2 / 3 | (A) Cefpodoxime–clavulanate 5 mg/kg p.o. once daily | 2.5% benzoyl peroxide shampoo twice weekly advised | 5 weeks | Furunculosis lesions healed after 14 days. Full resolution after 21 or 28 days (1 each)(underlying diseases treated) | – | Not reported |
| Reddy *et al.,* 2014b | Prospective case series | 3 | 2 / 3 | (A) Enrofloxacin 5 mg/kg p.o. once daily | 2.5% benzoyl peroxide shampoo twice weekly advised | 5–6 weeks | 1 achieved resolution by D21; 1 achieved resolution by D28  (underlying diseases treated) | – | Not reported |
| Reddy & Sivajothi, 2015 | Prospective case series | 3 | 9 / 9 | 1. Cefpodoxime proxetil   10 mg/kg p.o. once daily | 2% chlorhexidine gluconate / 2% miconazole shampoo advised (frequency not given) | ≥3 weeks and until up to 14 days after clinical resolution | Results discordant: Resolution of clinical signs, coat change, and pruritus in all *and* “significant recovery” in 8/9 (remaining one had methicillin resistant staphylococci)  Mean time to “significant recovery” (n = 8) was 22 ± 4 days | – | *Cytological evaluation*  Rods and cocci with neutrophils |
| Restrepo *et al.,* 2010 | Prospective case series | 3 | 8 / 8 | (A) Pradofloxacin 3 mg/kg [actual mean 3.7 mg/kg, range 3.0–4.6  mg/kg] p.o. once daily | Not permitted during study | 42 days | At 21 days: excellent response in 1; good response in 7 | At 42 days: complete resolution in 1; excellent response in 3; good response in 3; relapse in 1 | *Cytological evaluation* (n = 8)  Cocci in all, rods in one  D21: cocci in all, rods in one; reduction in numbers seen in most  D42: cocci in 6; reduction in numbers seen in all  *Culture*  Dogs n = 8: *S.[pseud]intermedius* in 6; no growth in 2 |
| Rosser, 1997 | Prospective case series | 3 | 12 / 12 | Various: cefalexin 22 mg/kg p.o. three times daily (n = 8/12); oxacillin 22 mg/kg three times daily (n = 2/12); erythromycin 11 mg/kg p.o. three times daily (n = 1/12); clindamycin 5 mg/kg p.o. twice daily (n = 1/12) | Benzoyl peroxide or povidone iodine shampoo once or twice weekly | 3–6 weeks | At 3 weeks: 1/12 clinical resolution(cefalexin) | At 5 weeks: 1/12 clinical remission (cefalexin)  At 6 weeks: 10/12 clinical remission (n = 6 cefalexin; n = 2 oxacillin; n = 1 erythromycin; n = 1 clindamycin) | *Culture*  One or more isolate from each dog (n = 12): 11 *S.[pseud] intermedius*; 4 beta–haemolytic streptococci; 3 *Proteus mirabilis*; 1 *Corynebacterium* spp. |
| Scott et al., 1993 | Prospective case series | 3 | 5 / 5 | (A) Ormetoprim–sulfadimethoxine 55  mg/kg day 1, 27.5  mg/kg p.o. once daily | Not permitted during study | 17–64 days | At D23–D24: 2/5 excellent response | At D39–D64: 3/5 excellent response | *Culture*  All: *S. [pseud]intermedius* susceptible to the respective study antimicrobial drug at enrolment  Follow–up swabs:  2 dogs not responding to tylosin 10 mg/kg twice daily had isolates resistant to tylosin after 14 days  2 dogs not responding to tylosin 20 mg/kg twice daily had isolates still susceptible to tylosin after 14 days |
|  |  |  |  |  |  |  | Treatment periods average: 29 days | |  |
| Scott et al., 1994 | Prospective case series | 3 | 9 / 9 | (A) Tylosin 20 mg/kg p.o. twice daily | Not permitted during study | 17–91 days | At days 24–27: 2/9 excellent response  Treatment discontinued in 1 at day 14 due to poor response | At days 36–91: 6/9 excellent response |  |
|  |  |  |  |  |  |  | Treatment period average 47 days | |  |
| Scott et al., 1996 | Prospective case series | 3 | 4 / 4 | (A) Tylosin 10 mg/kg p.o. twice daily | Not permitted during study | 20–29 days | At D21: 2/4 excellent response  Treatment discontinued in 1 at D20 due to poor response | At D29: 1/4 complete resolution |  |
|  |  |  |  |  |  |  | Treatment period average: 23 days | |  |
| Sentürk et al., 2005 | Prospective case series | 3 | 2 / 2 | (A) Rifampicin 5 mg/kg p.o. twice daily | Not described | 10 days | Poor response to treatment in both dogs at D10 | – | *Culture*  *Proteus* spp. in both (sensitivity testing not performed) |
| Sofou et al., 2022 | Prospective case series | 3 | 1 / 1 | Unknown [28 dogs in overall study]: amoxicillin–clavulanate [target dose 20–25  mg/kg; overall study actual dose 16.9–27.6 mg/kg] p.o. twice daily (n = 19) or clindamycin [target dose 11  mg/kg; overall study actual dose 10–12.3 mg/kg] p.o. once daily or enrofloxacin [10 mg/kg] p.o. once daily | –Not permitted during study | 28 days (until up to 14 days after clinical resolution) | 1/1 ”resolution confirmed clinically and ”cytologically” | – | Not reported |
|  | Retrospective case series | 3 | 1 / 1 |  | Unknown [overall study n = 17/19] 2% chlorhexidine gluconate / 2% miconazole nitrate shampoo 2–3 times weekly | 28 days (until up to 14 days after clinical resolution) | 1/1 “resolution confirmed clinically and cytologically” | – |  |
| Wisselink et al., 1985 | Prospective case series | 3 † | 1. 23 | 1. TMPS 5 mg/kg p.o. twice daily (n = unknown) 2. Lincomycin 40 mg/kg p.o. twice daily (n = unknown) | Povidone–iodine shampoo (frequency not presented) | 3–6 weeks | Partial resolution after 3–6 weeks of therapy (combined treatment groups  † Downgraded for lack of detail of clinical assessment after treatment (no numbers presented) | | *Culture* (n = 23/23)  100% coagulase-positive staphylococci; 8/23 in mixed culture |

RCT, randomised controlled trial; s.c.TMPS, trimethoprim–sulfonamide; MDR, multidrug-resistant

* Number of cases compatible with deep pyoderma based on description in text

† Study level of evidence (LoE) amended for reasons given under “””Outcome”

| **Level of evidence (LoE)** | | **Definition for treatment studies** |
| --- | --- | --- |
| 1 | Good quality, patient-orientated | “High quality” randomised controlled trial (RCT) OR meta–analysis of consistent RCTs with ≥10 dogs per group. |
| 2 | Limited quality patient–orientated | “Low quality” RCT downgraded either owing to <10 dogs per group, lack of separate assessment of groups, lack of specific clinical interpretation OR prospective case series (cohort study) containing ≥10 dogs |
| 3 | Other evidence | Prospective case series containing <10 dogs OR a retrospective case series (any size) |

# References

Angarano DW, MacDonald JM. Efficacy of cefadroxil in the treatment of bacterial dermatitis in dogs. J Am Vet Med Assoc. 1989;194:57–9.

Bettenay SV, Mueller RS, Dell”Osa D. Doxycycline hydrochloride in the treatment of canine pyoderma. Aust Vet Practit. 1998;28:14.

Cain CL, Mauldin EA. Clinical and histopathologic features of dorsally located furunculosis in dogs following water immersion or exposure to grooming products: 22 cases (2005–2013). J Am Vet Med Assoc. 2015;246:522–9.

Chirayath D, Rathish RL. Clindamycin for management of staphylococcal deep pyoderma – A clinical study of 12 dogs. Intas Polivet. 2015;16:321–2.

De Lucia M, Bardagi M, Fabbri E, Ferreira D, Ferrer L, Scarampella F, et al. Rifampicin treatment of canine pyoderma due to multidrug–resistant meticillin–resistant staphylococci: a retrospective study of 32 cases. Vet Dermatol. 2017;28:171-e36.

Frank LA, Kunkle GA. Comparison of the efficacy of cefadroxil and generic and proprietary cephalexin in the treatment of pyoderma in dogs. J Am Vet Med Assoc. 1993;203:530–3.

Gutierrez L, Tapia G, Ocampo L, Monroy–Barreto M, Sumano H. Oral plus topical administration of enrofloxacin–hydrochloride–dihydrate for the treatment of unresponsive canine pyoderma. a clinical trial. Animals (Basel). 2020;10:943.

Harbour L, Schick A, Mount R, White A. Rifampicin treatment of canine multidrug-resistant meticillin-resistant staphylococcal pyoderma: A retrospective study of 51 cases. Vet Dermatol. 2022;33:384–91.

Holm BR, Rest JR, Seewald W. A prospective study of the clinical findings, treatment and histopathology of 44 cases of pyotraumatic dermatitis. Vet Dermatol. 2004;15:369–76.

Krick SA, Scott DW. Bacterial folliculitis, furunculosis, and cellulitis in the German Shepherd dog: A retrospective analysis of 17 cases. J Am Anim Hosp Assoc. 1989;25:23–30.

Lloyd DH, Carlotti DN, Koch HJ, Van den Broek AH. Treatment of canine pyoderma with co–amoxyclav: a comparison of two dose rates. Vet Rec. 1997;141:439–41.

Marchegiani A, Spaterna A, Cerquetella M, Tambella AM, Fruganti A, Paterson S. Fluorescence biomodulation in the management of canine interdigital pyoderma cases: a prospective, single-blinded, randomized and controlled clinical study. Vet Dermatol. 2019;30:371–e109.

Marchegiani A, Fruganti A, Spaterna A, Cerquetella M, Tambella AM, Paterson S. The effectiveness of fluorescent light energy as adjunct therapy in canine deep pyoderma: a randomized clinical trial. Vet Med Int. 2021;2021:6643416.

Marchegiani A, Fruganti A, Gavazza A, Spaterna A, Cerquetella M. Fluorescence Biomodulation for canine interdigital furunculosis: updates for once-weekly schedule. Front Vet Sci. 2022;9:880349.

Mueller RS, Stephan B. Pradofloxacin in the treatment of canine deep pyoderma: a multicentred, blinded, randomized parallel trial. Vet Dermatol. 2007;18:144–51.

Paradis M, Lemay S, Scott DW, Miller WH, Wellington J, Panich R. Efficacy of enrofloxacin in the treatment of canine bacterial pyoderma. Vet Dermatol. 1990;1:123–7.

Paradis M, Abbey L, Baker B, Coyne M, Hannigan M, Joffe D, et al. Evaluation of the clinical efficacy of marbofloxacin (Zeniquin) tablets for the treatment of canine pyoderma: an open clinical trial. Vet Dermatol. 2001;12:163–9.

Parida GS, Das MR, Biswal S. Sahoo N. Therapeutic management of deep pyoderma in dogs. Intas Polivet. 2013;14:385–7.

Reddy BS, Nalini Kumari K, Vaikunta Rao V, Rayulu VC. Efficacy of cefpodoxime with clavulanic Acid in the treatment of recurrent pyoderma in dogs. ISRN Vet Sci. 2014:467010.

Reddy BS, Kumari KN, Rao VV, Rayulu VC, Sivajothi S. Efficacy of enrofloxacin in the treatment of recurrent pyoderma in dogs. J Adv Vet Res. 2014;4:108–12.

Reddy BS, Sivajothi S. Therapeutic management of deep pyoderma––a clinical study of nine dogs. Intas Polivet 2015;16:316–7.

Restrepo C, Ihrke PJ, White SD, Spiegel IB, Affolter VK. Evaluation of the clinical efficacy of pradofloxacin tablets for the treatment of canine pyoderma. J Am Anim Hosp Assoc. 2010;46:301–11.

Rosser EJ Jr. German shepherd dog pyoderma: a prospective study of 12 dogs. J Am Anim Hosp Assoc. 1997;33:355–63.

Scott DW, Miller WH, Wellington JR. The combination of ormetoprim and sulfadimethoxine in the treatment of pyoderma due to *Staphylococcus intermedius* infection in dogs. Canine Practice 1993:29–33.

Scott DW, Miller WH Jr, Cayatte SM, Bagladi MS. Efficacy of tylosin tablets for the treatment of pyoderma due to *Staphylococcus intermedius* infection in dogs. Can Vet J. 1994;35:617–21.

Scott DW, Miller WH Jr, Rothstein SE, Bagladi MS. Further studies on the efficacy of tylosin tablets for the treatment of pyoderma due to *Staphylococcus intermedius* infection in dogs. Can Vet J. 1996;37:617–8.

Scott DW, Beningo KE, Miller WH, Rothstein E. Efficacy of clindamycin hydrochloride capsules for the treatment of deep pyoderma due to *Staphylococcus intermedius* infection in dogs. Can Vet J. 1998;39:753–6.

Scott DW, Peters J, Miller WH Jr. Efficacy of orbifloxacin tablets for the treatment of superficial and deep pyoderma due to *Staphylococcus intermedius* infection in dogs. Can Vet J. 2006;47:999–1002.

Sentürk S, Özel E, Sen A. Clinical efficacy of rifampicin for treatment of canine pyoderma. Acta Vet. Brno. 2005;74:117–22.

Sofou EI, Aleksandrova S, Badulescu E, Chatzis M, Saridomichelakis M. Efficacy of antimicrobial treatment in dogs with atopic dermatitis: an observational study. Vet Sci. 2022;9:385.

Špruček F, Svoboda M, Toman M, Faldyna M, Špruček F Jr. Therapy of canine deep pyoderma with cephalexins and immunomodulators. Acta Vet. Brno.2007;76:469–74.

Stegemann MR, Coati N, Passmore CA, Sherington J. Clinical efficacy and safety of cefovecin in the treatment of canine pyoderma and wound infections. J Small Anim Pract. 2007;48:378–86.

Wisselink MA, Willemse A, Koeman JP. Deep pyoderma in the German Shepherd Dog. J Am Anim Hosp Assoc. 1985;21:773–6.
