# Supplementary material for: Antimicrobial use guidelines for canine pyoderma by the International Society for Companion Animal Infectious Diseases (ISCAID)
Source: Vet Dermatol. 2025 May 7;36(3):234–82. doi: 10.1111/vde.13342 (PMC12058580; doi:10.1111/vde.13342)
Supplement: Supplementary file 4 — Table S4. [file VDE-36-234-s003.docx]

| **Systematic review of systemic antimicrobial therapy in the treatment of SUPERFICIAL & DEEP PYODERMA** | | | | | | | | | |
| --- | --- | --- | --- | --- | --- | --- | --- | --- | --- |
| **Citation** | **Study design** | **SORT level of evidence** | **Study characteristics** | | | | | **Clinical outcomes** | **Microbiology information available**  **(results as reported at enrolment)** |
|  |  |  | **n completed / enrolled** | **Depth of pyoderma** | **Antimicrobial therapy** | **Adjunctive topical antimicrobial therapy** | **Treatment duration** |  |  |
| Bywater et al., 1985 | RCT | 1 | 57 / 57 | Generalised, superficial, interdigital | 1. Amoxicillin 10 or 20  mg/kg p.o. twice daily (n = 32) 2. Amoxicillin-clavulanate 12.5  mg/kg or 25  mg/kg p.o. twice daily (n = 25) | Not mentioned | Up to 30 days | Duration of treatment shorter for group (B) than group (A): 13.3±0.85 days versus 16.1±0.92 days (P<0.05)  Lack of difference between results of randomised controlled trial and case series, and between dose rates for the same agent, resulted in combination of data. | *Culture*  (n = 75): staphylococci in all |
|  | Prospective case series | 2 | 18 / 18 |  | (B) Amoxicillin-clavulanate 12.5 mg/kg or 25 mg/kg p.o. twice daily |  |  |  |  |
| Horspool *et al.,* 2004 | RCT | 1 | 228 / 245 | Superficial, deep (approx. 40%) | 1. Ibafloxacin 15 mg/kg p.o. once daily (n = 113) 2. Marbofloxacin 2 mg/kg p.o. once daily (n = 115) | Only nonantimicrobial shampoos permitted (and if started before study) | 3+ weeks (until 10–14 days after clinical resolution) | 1. Mean duration of treatment 40.5±25.2 days (mode 3 weeks). No response in 18/113 (16%). 2. Mean duration of treatment 37.6±21.2 days (mode 3 weeks). No response in 17/115 (15%).   No difference between treatments for percentage of dogs that maintained clinical resolution (*p*> 0.05) | *Culture*  (n = 245)   1. 85/113 (94%) *S. [pseud]intermedius*; 9 other staphylococci 2. 74/115 (92%) *S. [pseud]intermedius*; 17 other staphylococci |
| Horspool *et al.,* 2006 | RCT | 1 | 185 / 185 | Superficial (n = 107), deep (n = 78) | 1. Ibafloxacin tablets 15 mg/kg p.o. once daily (n = 93) 2. Ibafloxacin gel 15 mg/kg p.o. once daily (n = 92) | Only nonantimicrobial shampoos permitted (and if started before study) | Up to 13 weeks | 1. Resolved in 61%, improved in 19%, unchanged in 12%, worsened in 8%. Mean duration of treatment 44±26 days 2. Resolved in 77%, improved in 9%, unchanged in 12%, worsened in 2%. Mean duration of treatment 38±22 days. | *Culture*  (n = 166)   1. 17/80 (89%) *S. [pseud]intermedius*; other staphylococci in 4; Gram-negative bacteria in 5; no growth in 10 2. 79/86 (93%) *S. [pseud]intermedius* in 79; other staphylococci in 5; Gram-negative bacteria in 2; no growth in 2 |
| Carlotti *et al.,* 1999 | Prospective case series | 2 | 39 / 39 | Superficial (n = 9), deep (n = 30) | 1. Marbofloxacin 2  mg/kg [actual mean 2.12 mg/kg] p.o. once daily | Antiseptic shampoo (n = 27) | 10–213 days | Mean duration of treatment 55 days; with 33/39 (85%) having excellent response (resolution or very clear improvement), 1 having clear improvement, and 1 having improvement.  4 (10%) had no response (after 11–60 days treatment). | *Cytological evaluation*  All: degenerate neutrophils and phagocytosis of cocci in all  *Culture*  (n = 36): 50 isolates comprising: 21 *S. [pseud]intermedius*; 2 *S. aureus*; 8 Coagulase negative staphylococci; 8 Enterobacteriaceae; 5 *Pseudomonas* spp.; and 6 others |
| Cherni *et al.,* 2006 | RCT | 2† | 129 / 157 | Superficial pyoderma (n = 112); deep and surface pyoderma (n = 17) | 1. Cefpodoxime 5 mg/kg p.o. once daily (n = 63; 56 with superficial pyoderma) 2. Cefalexin 26 mg/kg p.o. twice daily (n = 66; 56 with superficial pyoderma) | Not permitted during study | 28–42 days | Treatment success (clinical signs mild to absent)   1. 61/63 (97%) overall; 48 (76%) by D28, additional 13 (21%) by D42 2. 62/62 (94%) overall; 55 (83%) by D28, additional 9 (14%) by D42   Treatment (A) considered noninferior to treatment (B)  † Downgraded for lack of differentiation in outcomes between superficial and deep pyoderma | *Culture*  (n = 129): 113 had *S. [pseud]intermedius*; 80 had mixed infection; no growth in 2 |
| Keefe & Christie*,* 1973 | Prospective case series | 2 | 14 / 14 | Undescribed | 1. Dicloxacillin 11–55  mg/kg p.o. twice or three‑times daily | Not mentioned | 10+ days | 3/14 (21%) resolved, 9/14 (64%) improvement obvious, 1/14 (7%) some improvement, 1/14 (7%) no improvement / deterioration  Culture of two unsatisfactory responses showed Gram-negative organisms alongside staphylococci | *Culture*  (n = 14): penicillin-resistant *Staphylococcus* spp. in all; mixed growth (with Gram-negative organisms) in 2 |
| Mahto & Bismas, 2013 | Prospective case series | 2 | 10 / 10 | Superficial (n = 7), deep (n = 3) | Lincomycin 22 mg/kg p.o. twice daily | Chlorhexidine shampoo | 14 days | All animal recovered after 2 weeks | *Cytological evaluation*  (n = 10): cocci in all |
| Meena et al., 2019 | Prospective case series | 2 | 17 / 17 | Undescribed | 1. Amikacin   at 15 mg/kg i.m. once daily for 3 weeks (n = 11) *or*  for 1 week, then enrofloxacin 10  mg/kg p.o. once daily for 3 weeks (n = 5) *or* then azithromycin 10 mg/kg p.o. once daily for 3 weeks (n = 1) | Not mentioned | 21 28 days | Complete resolution by 14 days | *Culture*  (n = 30; overall study): *Staphylococcus* spp. in all |
| Seena et al., 2005 | Prospective case series | 2 | 18 / 18 | Surface (n = 2), superficial (n = 11), and deep (n = 5) | 1. TMPS 30 mg/kg twice daily (n = 6) 2. Lincomycin 22 mg/kg p.o. twice daily (n = 6) 3. Cefalexin 25 mg/kg p.o. twice daily (n = 6) | Not mentioned | 14 28 days (reviewed weekly for total of 4 weeks) | 1. 3 recovered by 2 weeks,1 more by 3 weeks. Treatment failed in 2 (subsequent response to cefalexin). 2. 4 recovered by 2 weeks, 1 more by 4 weeks. Treatment failed in 1 (subsequent response to cefalexin).   (C) 2 recovered by 2 weeks, 2 more by 3 weeks, and remaining 2 by 4 weeks. | *Culture*  (n = 18): *S. [pseud]intermedius* in 11; *S. aureus* in 6; 1 each of atypical coagulase-positive staphylococci, *Pseudomonas* spp., and *Klebsiella* spp. |
| Beigh et al., 2013 | Prospective case series | 3† | 12 / 23 | Superficial (n = 17); deep (n = 6)  NB: unknown which 12 were given treatment | 1. Cefalexin 22 mg/kg p.o. twice daily (n = 6) 2. Cefalexin 22 mg/kg p.o. twice daily (n = 6) plus zinc sulfate | Not mentioned | 1. 14 days 2. 10 days | ”The animals respond well to the antibiotic” (no further details given, including timing of follow-up)  † Downgraded for lack of detail in clinical assessment | *Culture*  (n = not presented): 47% *S. [pseud]intermedius*; 31% *S. aureus*; 7% *Streptococcus* spp.; 14% mixed culture |
| Bryan et al., 2012 | Retrospective case series | 3 | 179 / 216 | Not described | *MSSP (n = 114)*  Cefalexin (n = 54): 20.4–30.9 mg/kg twice daily  Cefpodoxime (n = 43): 5.2–16.7 mg/kg once daily  Clindamycin (n = 8): 10.7–14.5 mg/kg twice daily  Amoxicillin-clavulanate (n = 6): 13.6–26.5 mg/kg twice daily  Doxycycline (n = 3): 4.1–6.7 mg/kg twice daily  Trimethoprim-sulfamethoxazole (n = 1): 32.8 mg/kg once daily  *MRSP (n = 65)*  Chloramphenicol (n = 51): 30.6–61.9 mg/kg three times daily  Doxycycline (n = 15): 4.1–11.1 mg/kg twice daily  Clindamycin (n = 6): 7.1–11.5 mg/kg twice daily  Minocycline (n = 3): 7.1–9.8 mg/kg twice daily  Amikacin (n = 2): 15.0-15.8 mg/kg once daily  Amoxicillin-clavulanate (n = 2): 23.4–-24.4 mg/kg twice daily  Marbofloxacin (n = 2): 4.5–5.5 mg/kg twice daily  Enrofloxacin (n = 1): 2.6 mg/kg once daily  TMPS (n = 1): 25 mg/kg twice daily  NB: these include dogs that received more than one antimicrobial during the treatment period | Data not given (only dogs given topical treatment alone recorded, n = 37) | 21+ days  (reviewed every 3-4 weeks) | Follow-up data (1 or more visit) for 164/216 cases (unclear which were topical therapy alone)  *MSSP (n = 88)*  At 3–4 weeks (first visit): 43 (49%) had resolved  Final outcome (median 1 visit; range 1–4 visits): 64 (73%) had resolved, 21 (24%) had improved, 3 (3%) had no improvement  *MRSP (n = 76)*  At 3–4 weeks (first visit): 29 (38%) had resolved  Final outcome (median 1 visit; range 1–8 visits): 47 (62%) had resolved, 22 (29%) had improved, and 7 (9%) had no improvement | *Culture*  (n = 216): *S. pseudintermedius* in all [MSSP in 123; MRSP in 93] |
| Davis *et al.,*1972 | Prospective case series | 3† | 25 / 25 | Dermatitis | 1. Ampicillin 11–22  mg/kg p.o. 1–4 times daily | ”Concurrent therapy” mentioned but no details given | Unknown | No data on exact dose or frequency; frequency of administration ”had little effect on response”.  Excellent response 8/25 (32%), good response 8/25 (32%), poor response 9/25 (36%).  † Downgraded for lack in detail on dosing | *Culture*  (n = 15): staphylococci, streptococci, *Proteus* spp., and *Pseudomonas* spp. isolated most frequently |
| Farca *et al.,* 1997 | Prospective case series | 3 | 5 / 5 | Chronic dermatitis that had not resolved with systemic therapy alone | Cephaloridine (10 mg/kg),  kanendomycin (2.5 mg/kg), or enrofloxacin (5 mg/kg) p.o. twice daily | Local application of EDTA-tromethamine three times daily for 14 days | 10 days | Clinical resolution within 7–10 days in all | *Culture*  (n = 5): *S. aureus* in 4; *Proteus mirabilis* in 3; *E. coli* in 1; mixed growth in 3 |
| Hillier *et al.,* 2006 | Retrospective case series | 3 | 18 / 20 | Superficial (n = 4), deep (n = 16) | Systemic treatment (n = 18)  Enrofloxacin 6–13 mg/kg p.o. once daily (n = 6) or 5-12 mg/kg p.o. twice daily (n = 2); or  Norfloxacin 18-23 mg/kg p.o. once daily (n = 2) or 11-22 mg/kg p.o. twice daily (n = 3); or M  arbofloxacin 3-5 mg/kg p.o. once daily (n = 3); or  Cefalexin 20-25 mg/kg p.o. twice daily (n = 2) | Once weekly shampoo with benzoyl peroxide (n = 5), chlorhexidine (n = 2), ethyl lactate (n = 2)  1 dog had topical treatment alone (1% silver sulfadiazine cream twice daily) | 3-12 weeks | Complete resolution in 16/19 (including one with topical alone) and partial improvement in 1  Mean duration of treatment (combined results): 4.8 weeks  Two lost to follow-up | *Cytological evaluation*  (n = 12): neutrophils present in all; bacteria present in 6 (rods in 5, cocci in 2)  *Culture*  (n = 20): *Pseudomonas aeruginosa* in all |
| Jun *et al.,* 2008 | Prospective case series* | 3 | 4 / 4 | Undescribed | 1. Amikacin 20 mg/kg IM once daily | Twice weekly bathing with shampoo (n = 4; unknown active ingredients) | 14 days | Clinical signs”much improved” | *Culture*  (n = 4): *Staphylococcus* spp. in all |

RCT, randomised controlled trial; MRSP, meticillin-resistant *S. pseudintermedius*; MSSP, meticillin-susceptible *S. pseudintermedius;* TMPS, Trimethoprim-sufamethoxazole

*This was a nonrandomised clinical trial that assesses another metric, but as an assessment of systemic antimicrobial therapy of superficial / deep pyoderma it is considered a case series.

‡This study was a randomised controlled trial that assesses another metric, but to assess systemic therapy of pyoderma, it has been downgraded to case series.

† Study level of evidence (LoE) amended for reasons given under ”Outcome”

| **Level of evidence (LoE)** | | **Definition for treatment studies** |
| --- | --- | --- |
| 1 | Good quality, patient-orientated | “High quality” randomised controlled trial OR meta-analysis of consistent RCTs with ≥10 dogs per group. |
| 2 | Limited quality patient-orientated | “Low quality” RCT downgraded either owing to <10 dogs per group, lack of separate assessment of groups, lack of specific clinical interpretation OR prospective case series (cohort study) containing ≥10 dogs |
| 3 | Other evidence | Prospective case series containing <10 dogs OR a retrospective case series (any size) |

# References

Beigh SA, Soodan JS, Tantary H, Tikoo A. Comparative evaluation of antibacterial alone and antibacterial along with zinc in management of pyoderma in canines. Intas polivet. 2013;14:388–90.

Bryan J, Frank LA, Rohrbach BW, Burgette LJ, Cain CL, Bemis DA. Treatment outcome of dogs with meticillin-resistant and meticillin-susceptible *Staphylococcus pseudintermedius* pyoderma. Vet Dermatol. 2012;23:361–8, e65.

Bywater RJ, Hewett GR, Marshall AB, West B. Efficacy of clavulanate-potentiated amoxycillin in experimental and clinical skin infections. Vet Rec. 1985;116:177–9.

Carlotti DN, Guaguere E, Pin D, Jasmin P, Thomas E, Guiral V. Therapy of difficult cases of canine pyoderma with marbofloxacin: a report of 39 dogs. J Small Anim Pract.1999;40:265–70.

Cherni JA, Boucher JF, Skogerboe TL, Tarnacki S, Gajewski KD, Lindeman CJ. Comparison of the efficacy of cefpodoxime proxetil and cephalexin in treating bacterial pyoderma in dogs. Intern J Appl Res Vet Med. 2006;4:85–93.

Davis WT, Reynolds WA, Maplesden DC. Clinical efficacy of ampicillin capsules in dogs & cats. Vet Med Small Anim Clin. 1972,550–5.

Farca AM, Piromalli G, Maffei F, Re G. Potentiating effect of EDTA-Tris on the activity of antibiotics against resistant bacteria associated with otitis, dermatitis and cystitis. J Small Anim Pract. 1997;38:243–5.

Hillier A, Alcorn JR, Cole LK, Kowalski JJ. Pyoderma caused by *Pseudomonas aeruginosa* infection in dogs: 20 cases. Vet Dermatol. 2006;17:432–9.

Horspool LJ, van Laar P, van den Bos R, Mawhinney I. Treatment of canine pyoderma with ibafloxacin and marbofloxacin--fluoroquinolones with different pharmacokinetic profiles. J Vet Pharmacol Ther. 2004;27:147–53.

Horspool LJ, van Laar P, van den Bos R, Mawhinney IC. Clinical efficacy of two ibafloxacin formulations in the treatment of canine pyoderma. Vet Rec. 2006;158:236–7.

Jun HK, Kim SH, Kim CMH, Cho SW, Jun MH, You MJ, et al.  Therapeutic effect of aquapuncture with bee-venom for canine pyoderma.  J Vet Clin 2008;25:471–5.

Keefe TJ, Christie GJ. Sodium dicloxacillin monohydrate: an anti-staphylococcus antibiotic. Vet Med Small Anim Clin. 1973;905–8.

Mahto RP, Biswas S. Therapeutic management of canine pyoderma – a study of 10 patients. Intas Polivet. 2013;14:391–2.

Meena A, Thirunavukkarasu P, Santhanakumar K, Murugesan AC. Efficacy of amikacin in the management of recurrent staphylococcal pyoderma in dogs. Indian Vet J. 2019;96:49–50.

Seena VB, Kumari KN, Singari NA, Sreedevi B. Clinico-diagnostic and therapeutic studies of canine pyoderma. Indian J Vet Med. 2005;2:121-2.
